# Supplementary figures and images for: CDC42 supports HBV entry by NTCP translocation to the plasma membrane and macropinocytosis (part 1 of 2)
Source: EMBO Rep. 2025 Sep 15;26(21):5239–69. doi: 10.1038/s44319-025-00581-8 (PMC12592336; doi:10.1038/s44319-025-00581-8)

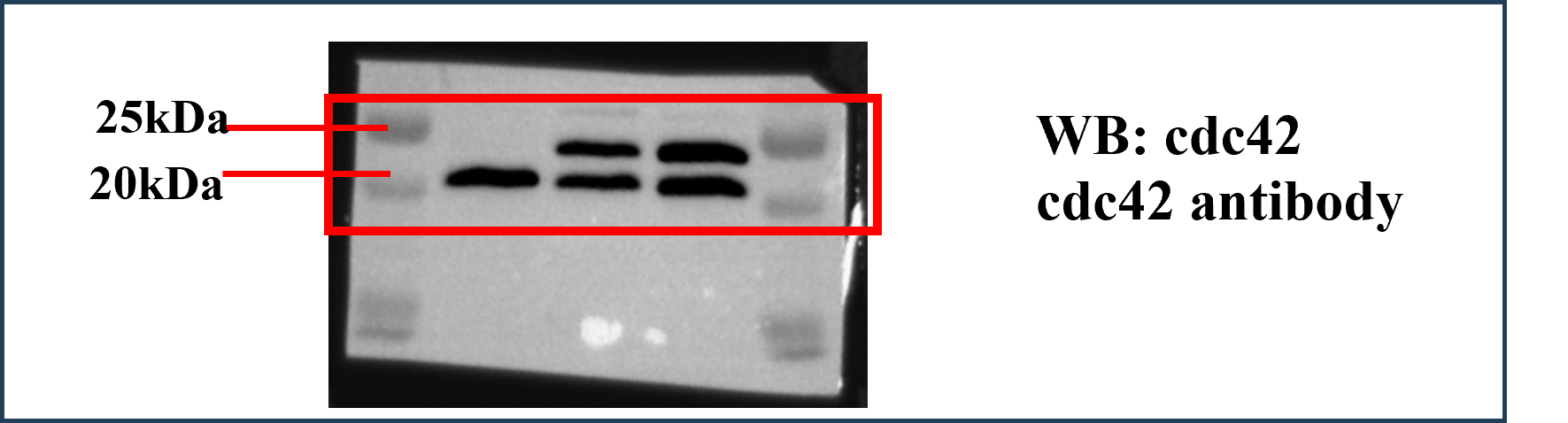

Supplement: Supplementary file 2 — Source data Fig. 1 [file 44319_2025_581_MOESM2_ESM.zip › 1 A/CDC42 input.tif]

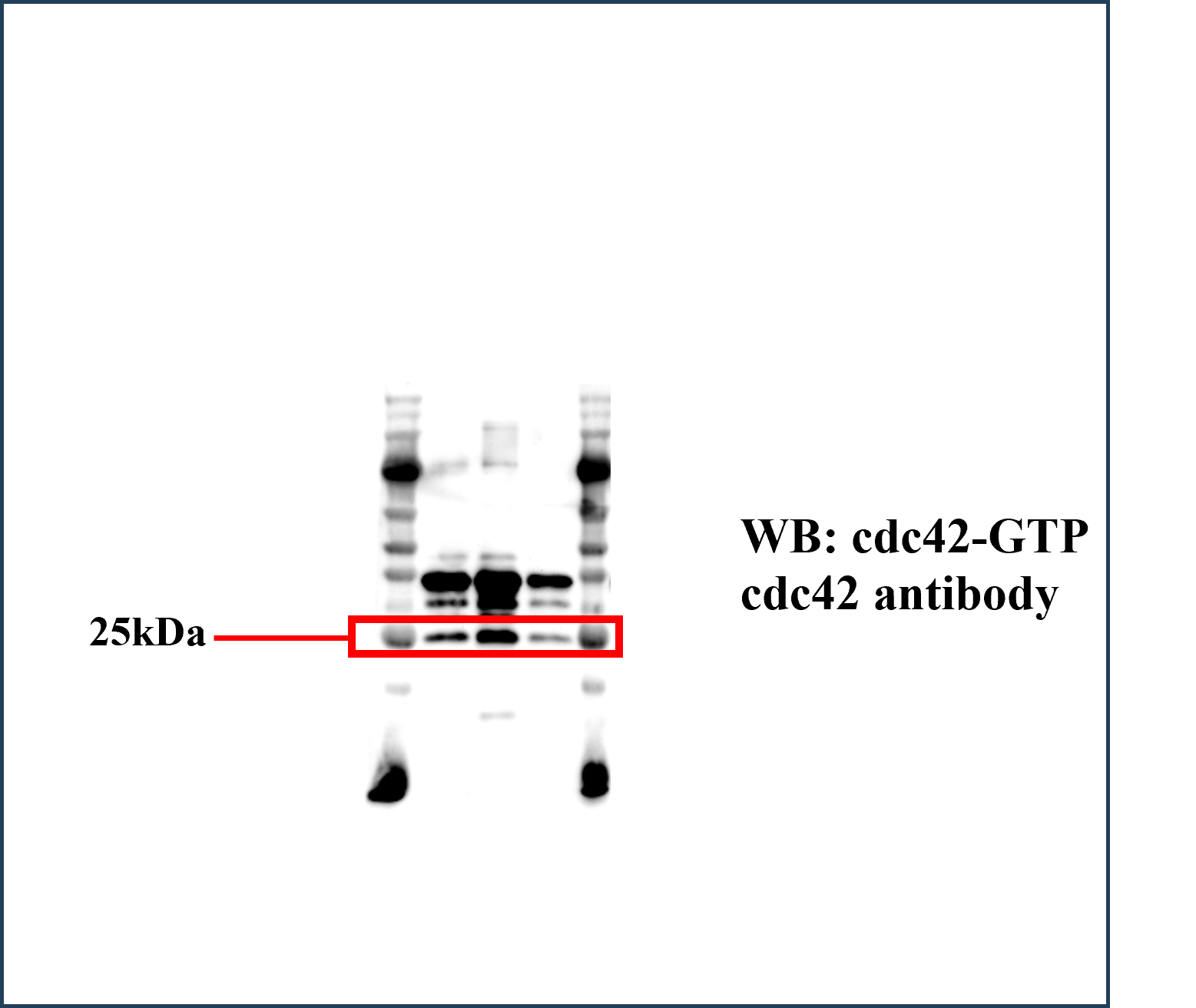

Supplement: Supplementary file 2 — Source data Fig. 1 [file 44319_2025_581_MOESM2_ESM.zip › 1 A/CDC42-GTP.tif]

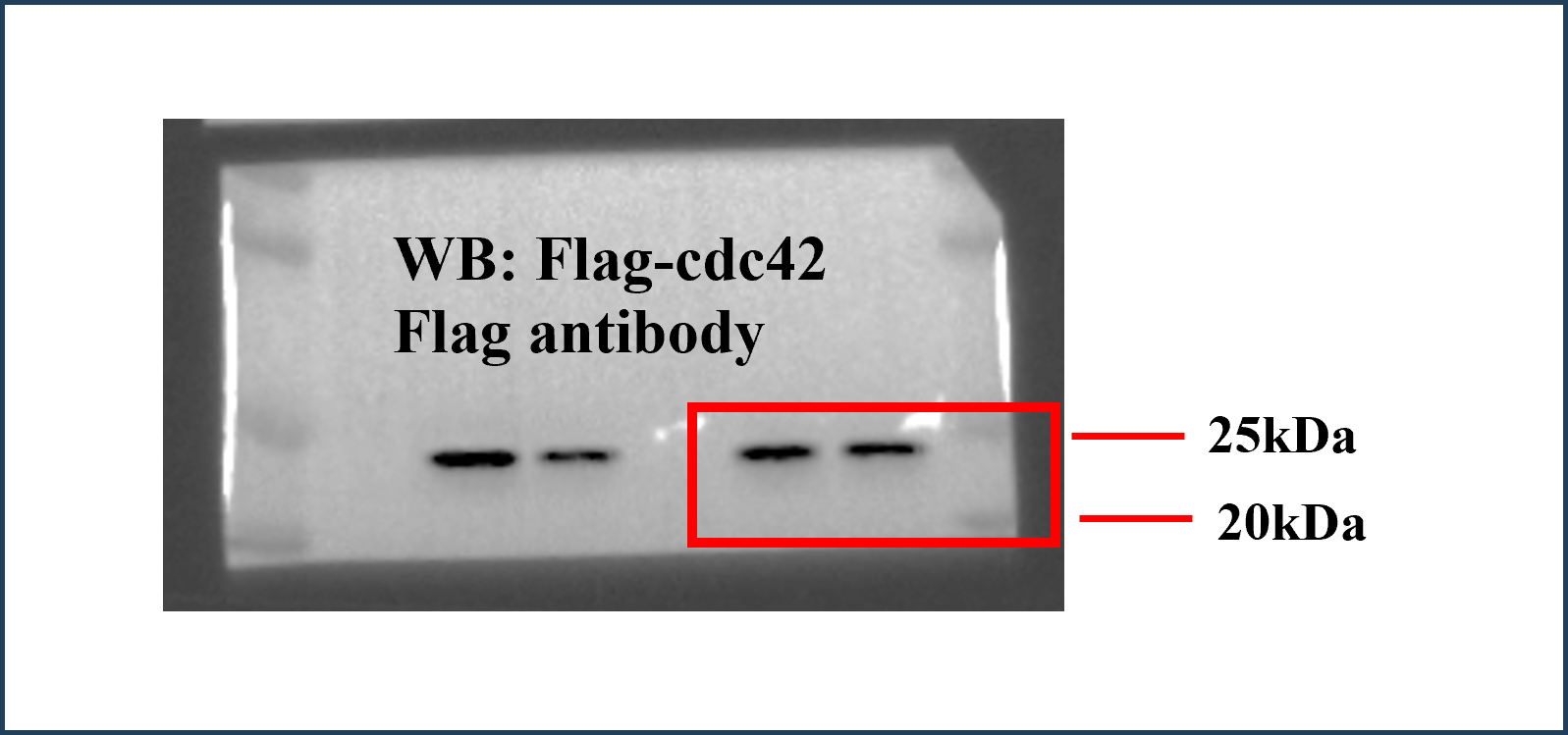

Supplement: Supplementary file 2 — Source data Fig. 1 [file 44319_2025_581_MOESM2_ESM.zip › 1 A/Flag.tif]

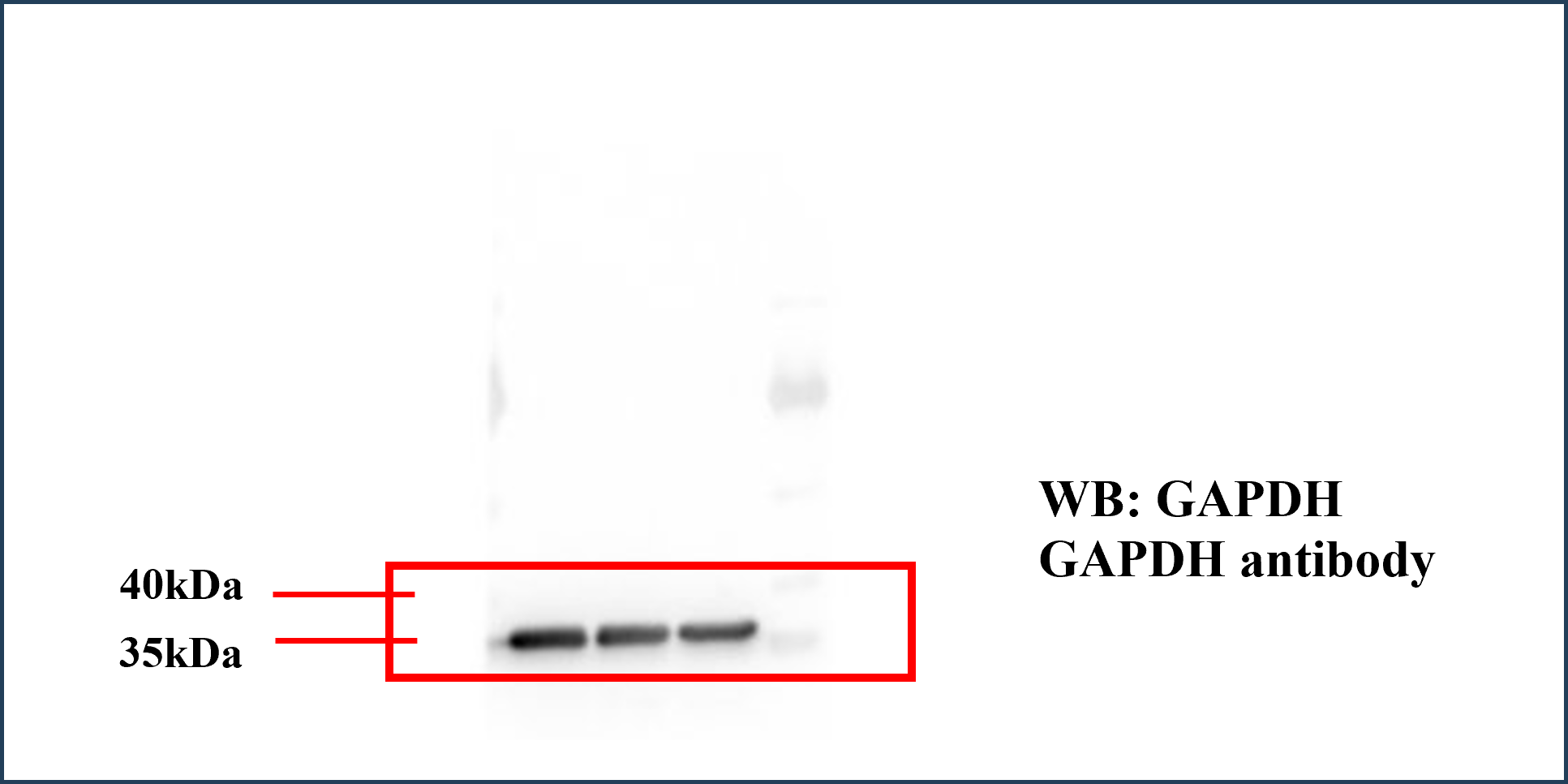

Supplement: Supplementary file 2 — Source data Fig. 1 [file 44319_2025_581_MOESM2_ESM.zip › 1 A/GAPDH.tif]

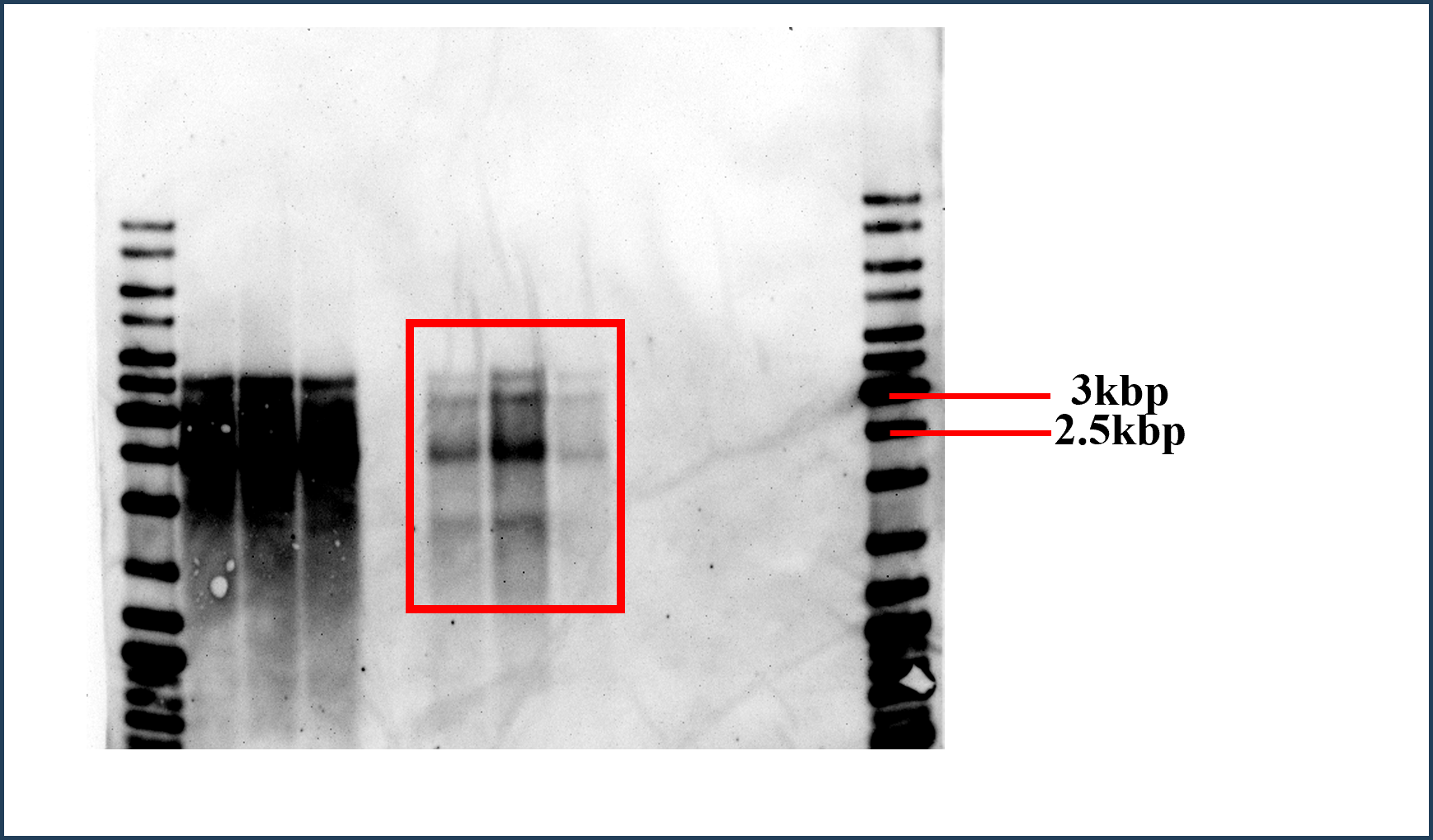

Supplement: Supplementary file 2 — Source data Fig. 1 [file 44319_2025_581_MOESM2_ESM.zip › 1 B/HBV DNA southern.tif]

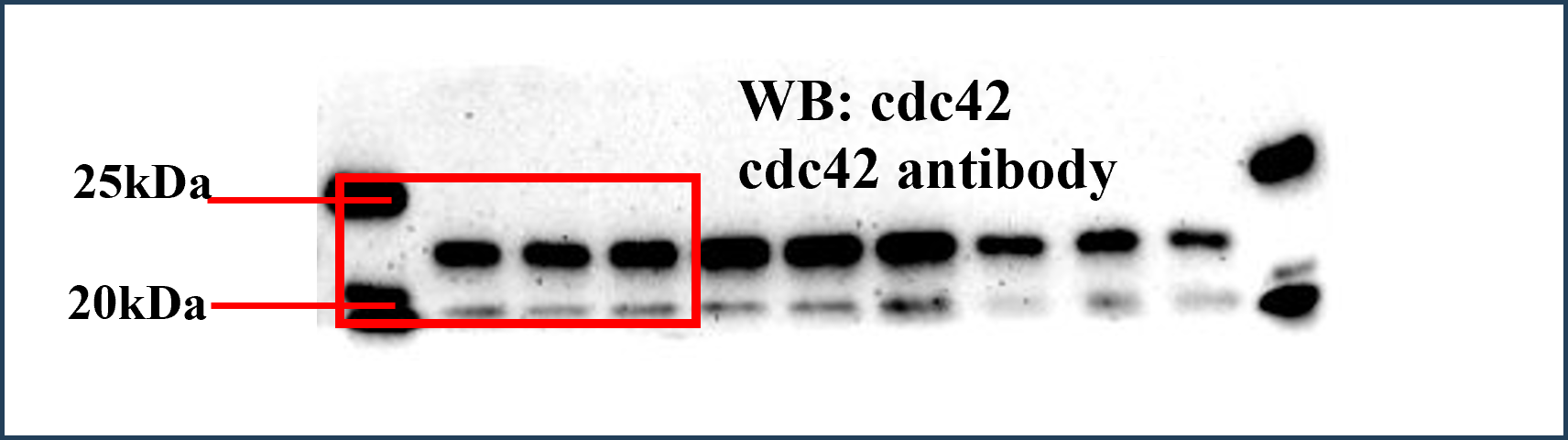

Supplement: Supplementary file 2 — Source data Fig. 1 [file 44319_2025_581_MOESM2_ESM.zip › 1 F/CDC42 input.tif]

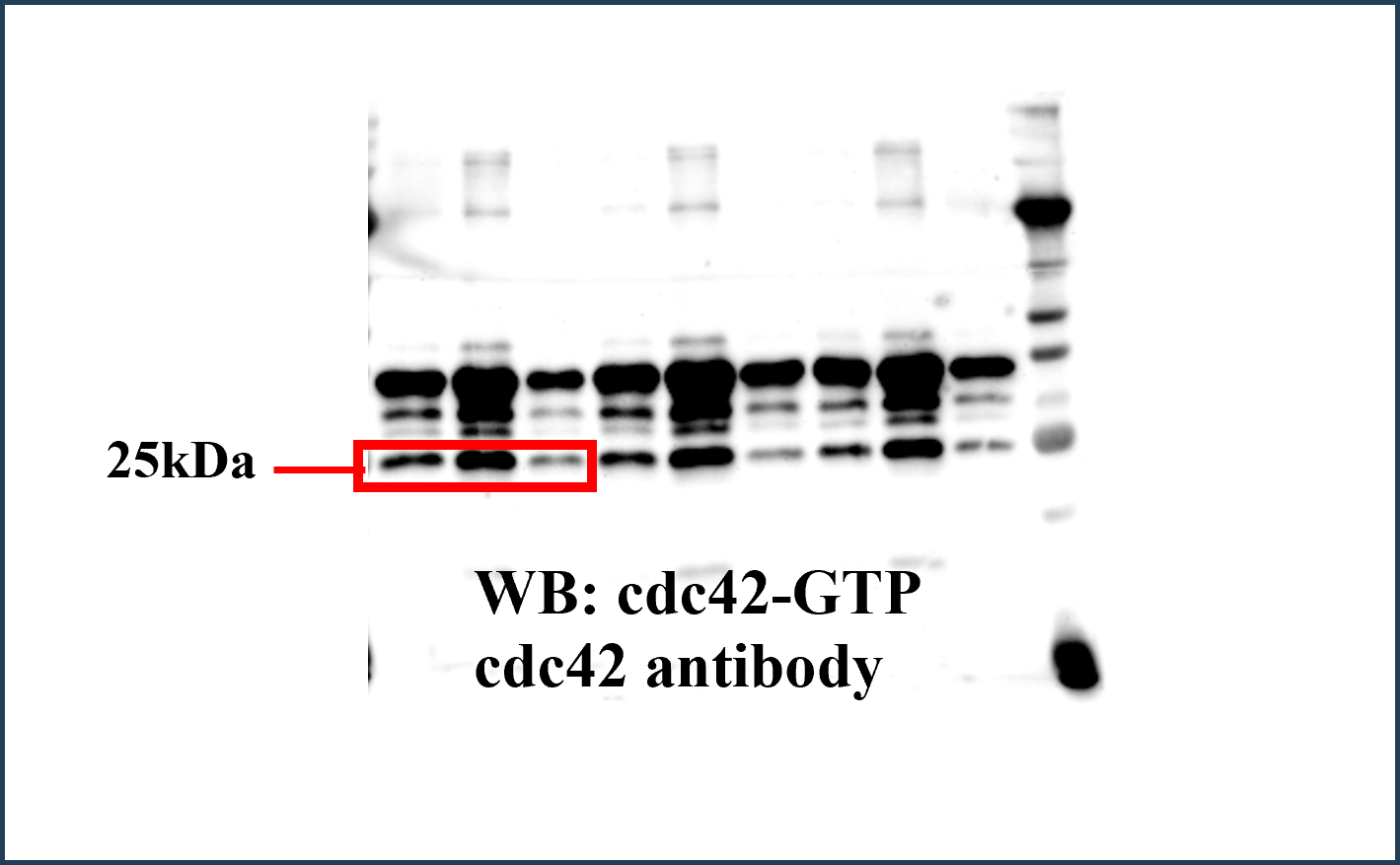

Supplement: Supplementary file 2 — Source data Fig. 1 [file 44319_2025_581_MOESM2_ESM.zip › 1 F/CDC42-GTP.tif]

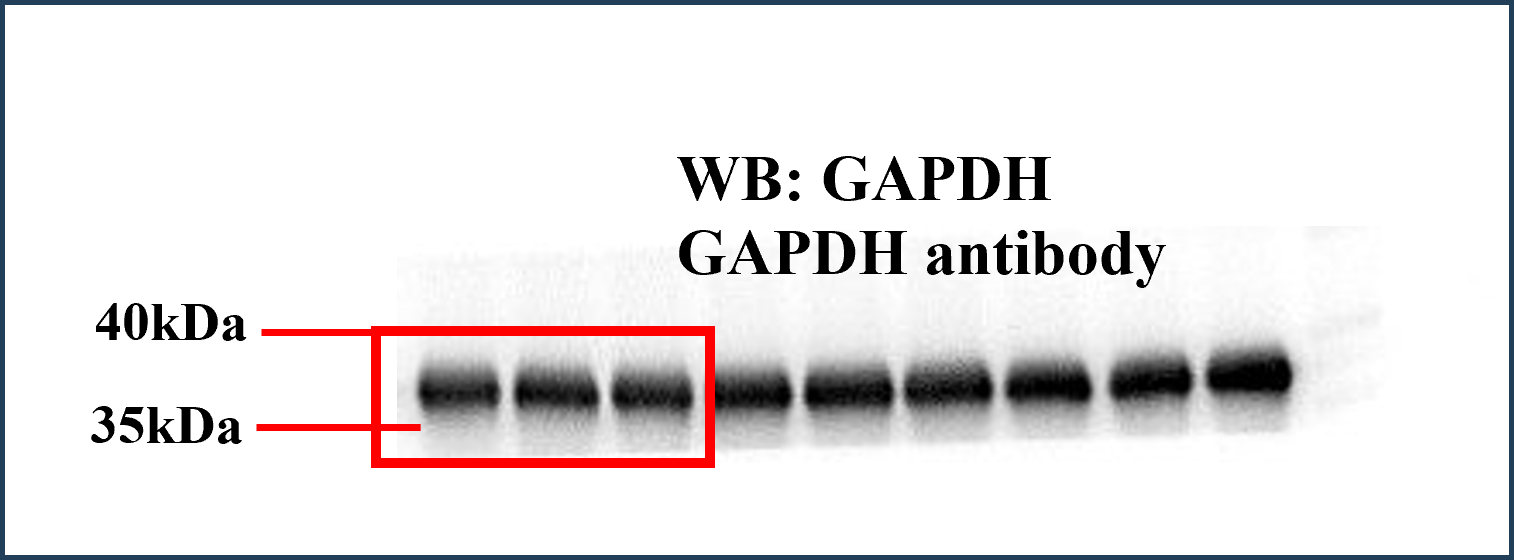

Supplement: Supplementary file 2 — Source data Fig. 1 [file 44319_2025_581_MOESM2_ESM.zip › 1 F/GAPDH.tif]

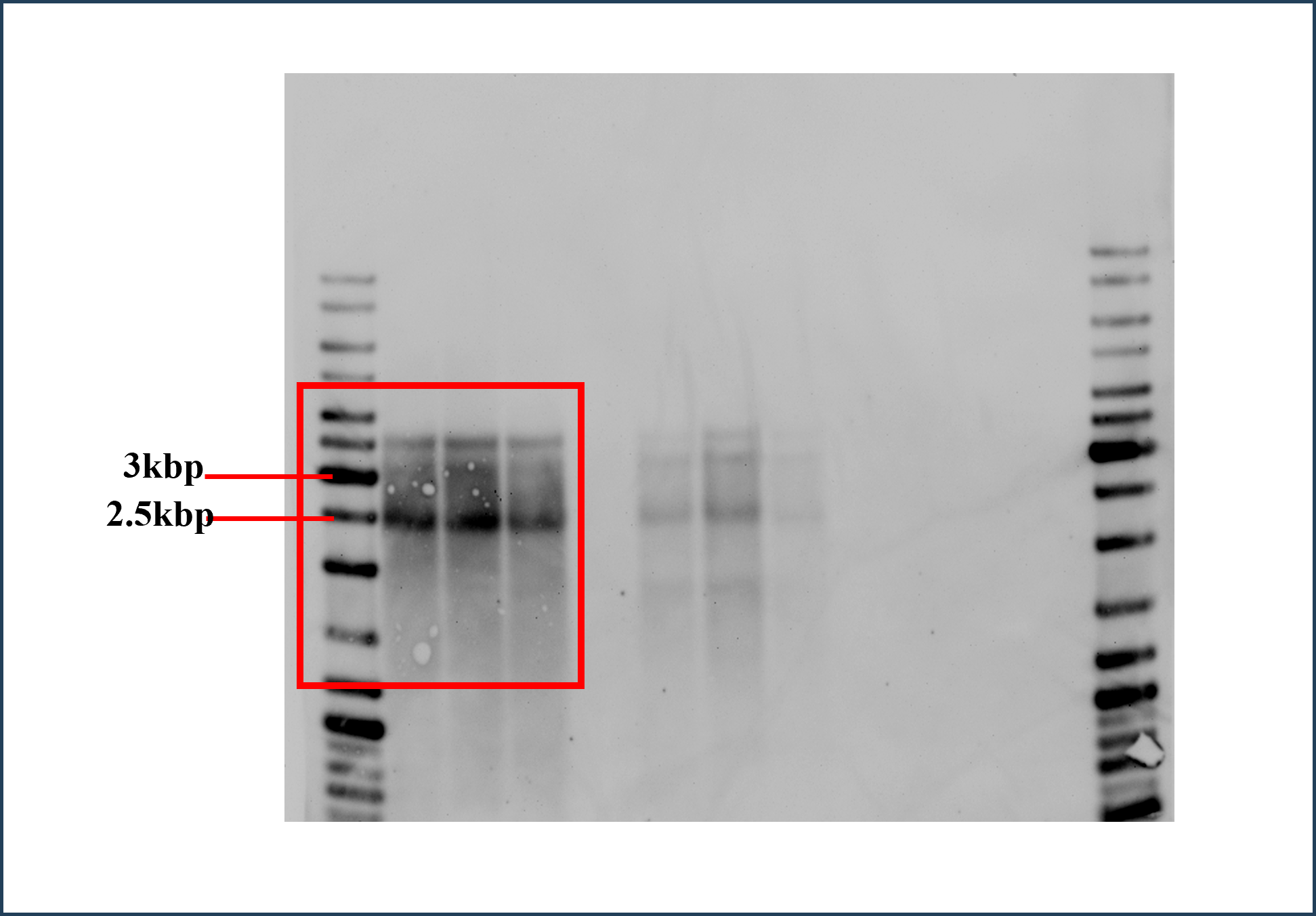

Supplement: Supplementary file 2 — Source data Fig. 1 [file 44319_2025_581_MOESM2_ESM.zip › 1 G/HBV DNA southern.tif]

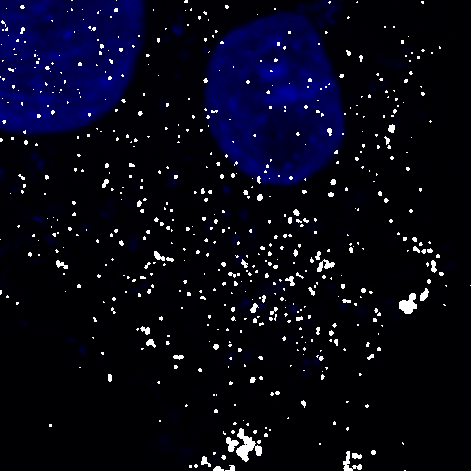

Supplement: Supplementary file 3 — Source data Fig. 2 [file 44319_2025_581_MOESM3_ESM.zip › 2 F/Cdc42 CA Bottom.tif]

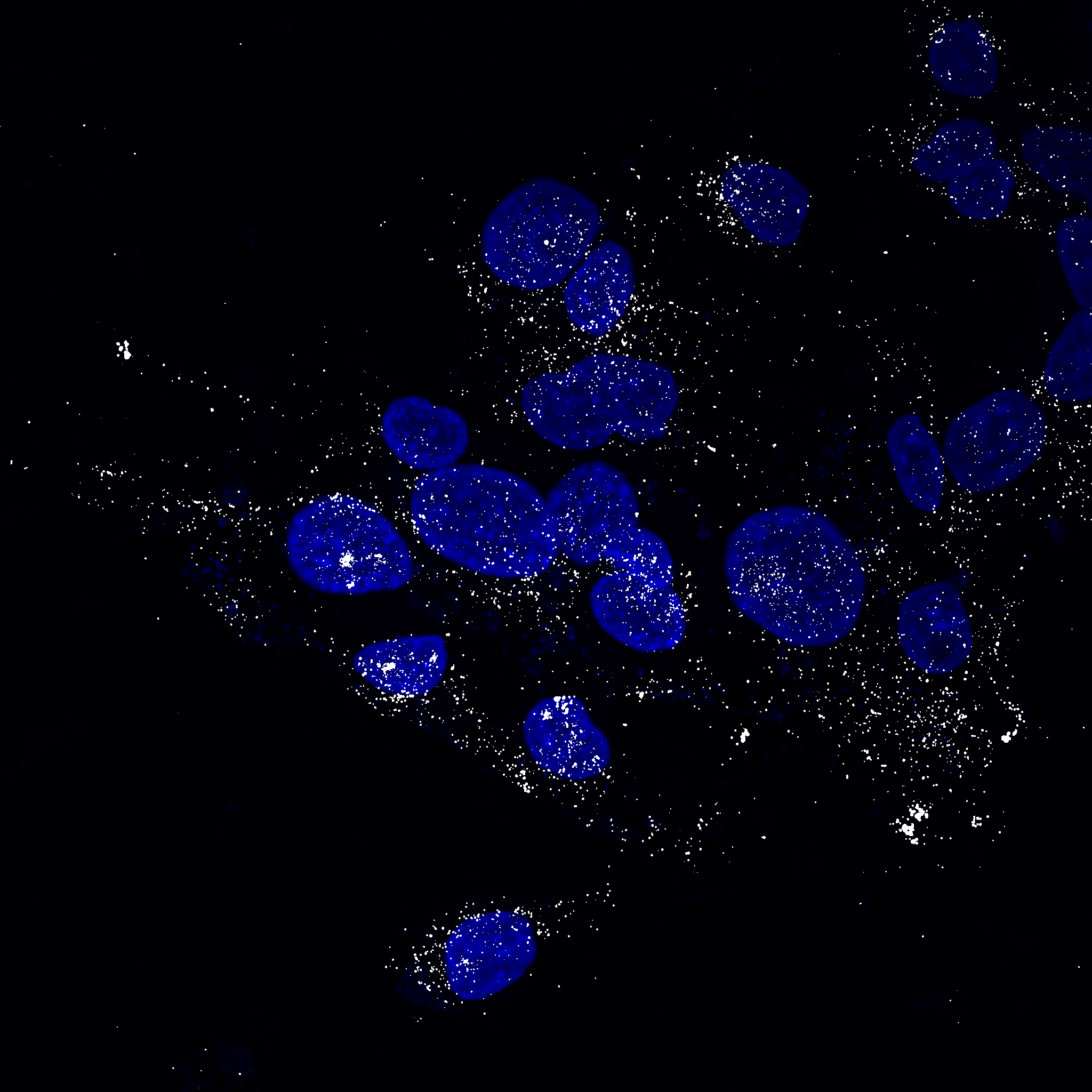

Supplement: Supplementary file 3 — Source data Fig. 2 [file 44319_2025_581_MOESM3_ESM.zip › 2 F/Cdc42 CA Top.tif]

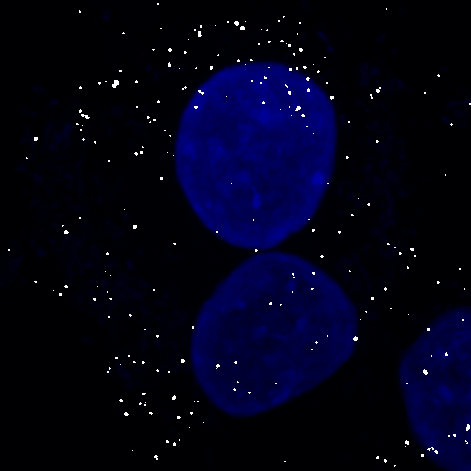

Supplement: Supplementary file 3 — Source data Fig. 2 [file 44319_2025_581_MOESM3_ESM.zip › 2 F/Cdc42 DN Bottom.tif]

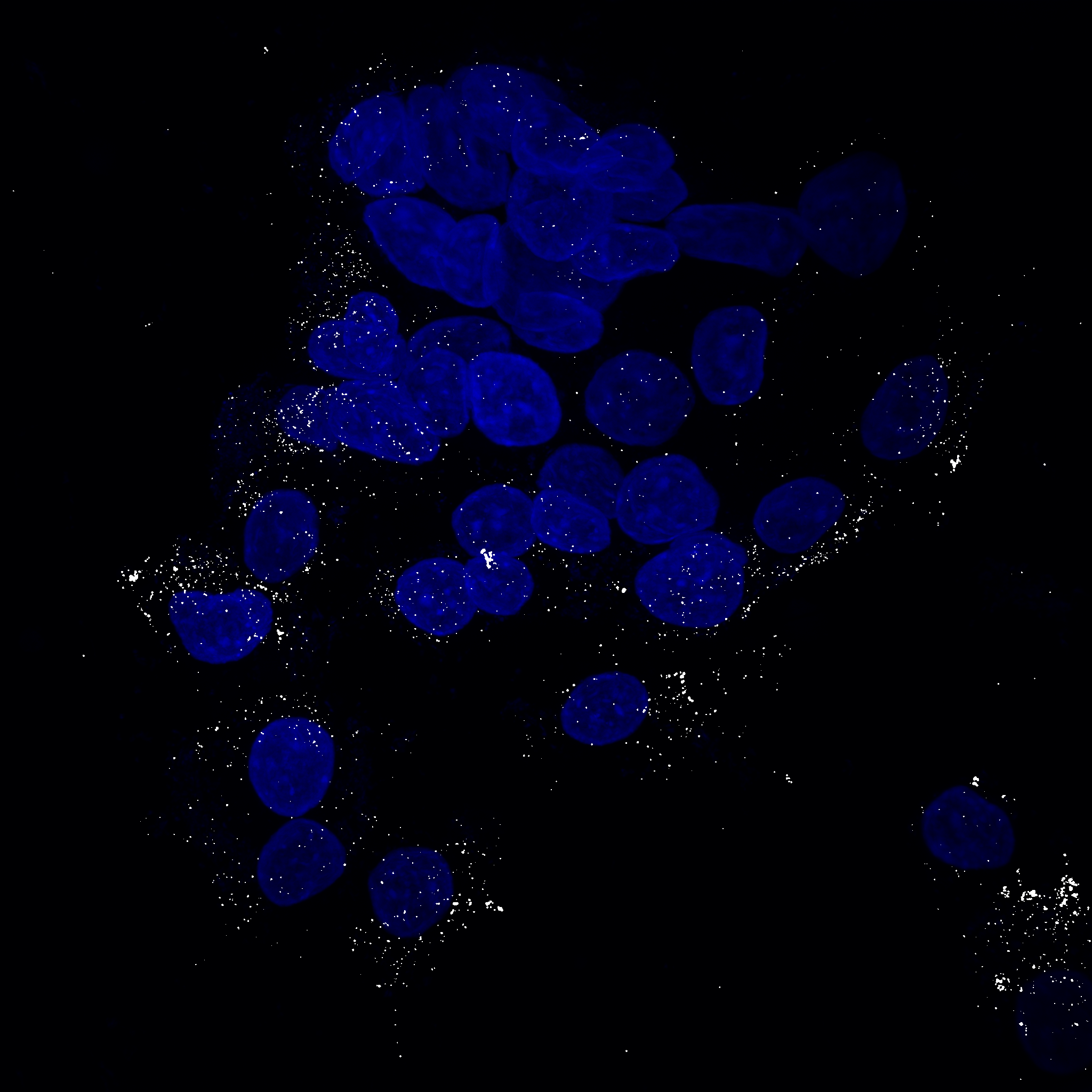

Supplement: Supplementary file 3 — Source data Fig. 2 [file 44319_2025_581_MOESM3_ESM.zip › 2 F/Cdc42 DN Top.tif]

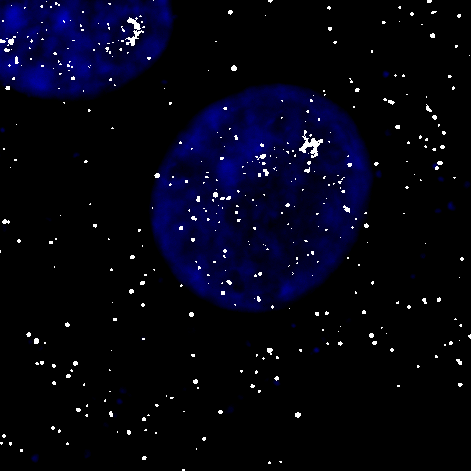

Supplement: Supplementary file 3 — Source data Fig. 2 [file 44319_2025_581_MOESM3_ESM.zip › 2 F/Cdc42 WT Bottom.tif]

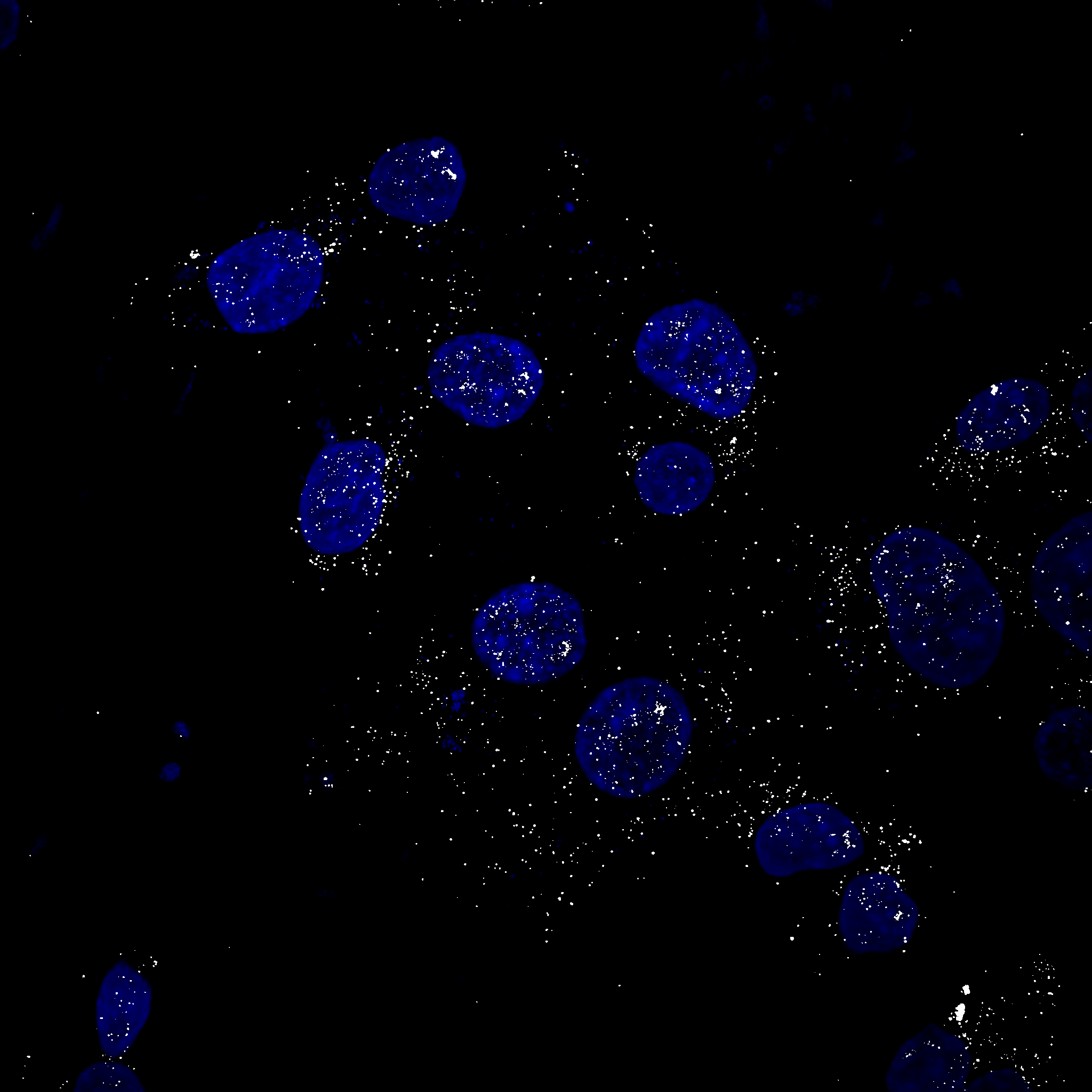

Supplement: Supplementary file 3 — Source data Fig. 2 [file 44319_2025_581_MOESM3_ESM.zip › 2 F/Cdc42 WT Top.tif]

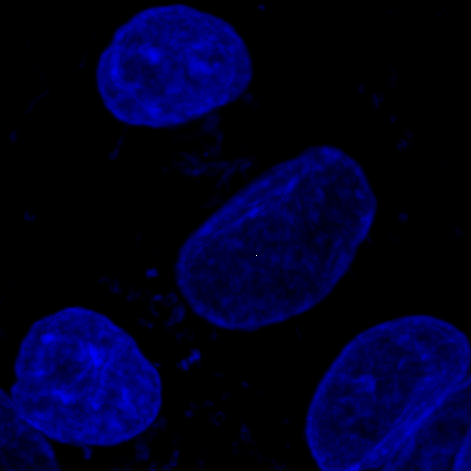

Supplement: Supplementary file 3 — Source data Fig. 2 [file 44319_2025_581_MOESM3_ESM.zip › 2 F/Noninfected Bottom.tif]

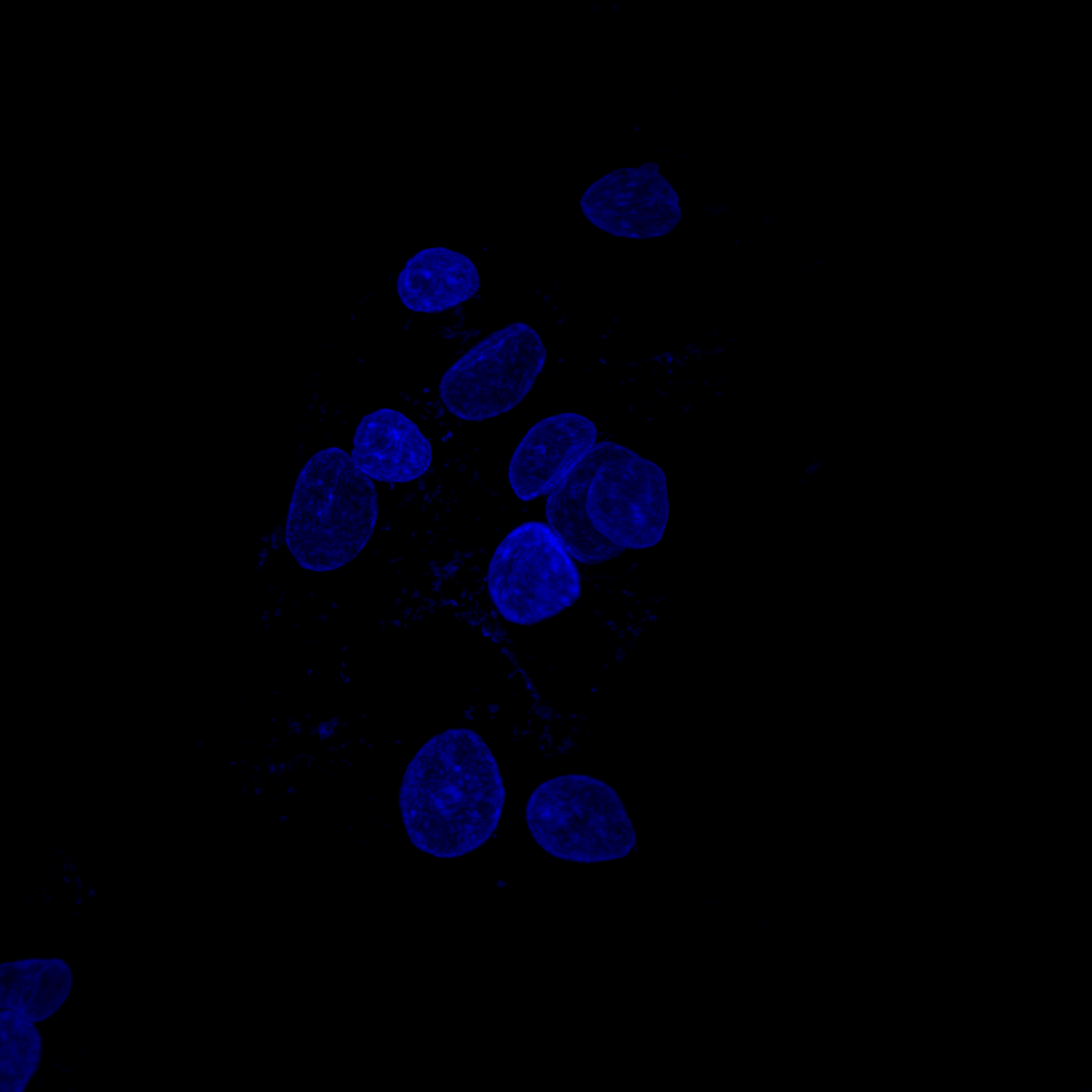

Supplement: Supplementary file 3 — Source data Fig. 2 [file 44319_2025_581_MOESM3_ESM.zip › 2 F/Noninfected Top.tif]

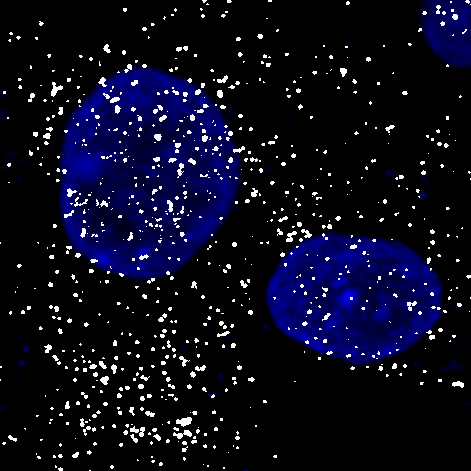

Supplement: Supplementary file 3 — Source data Fig. 2 [file 44319_2025_581_MOESM3_ESM.zip › 2 H/Bradykinin Bottom.tif]

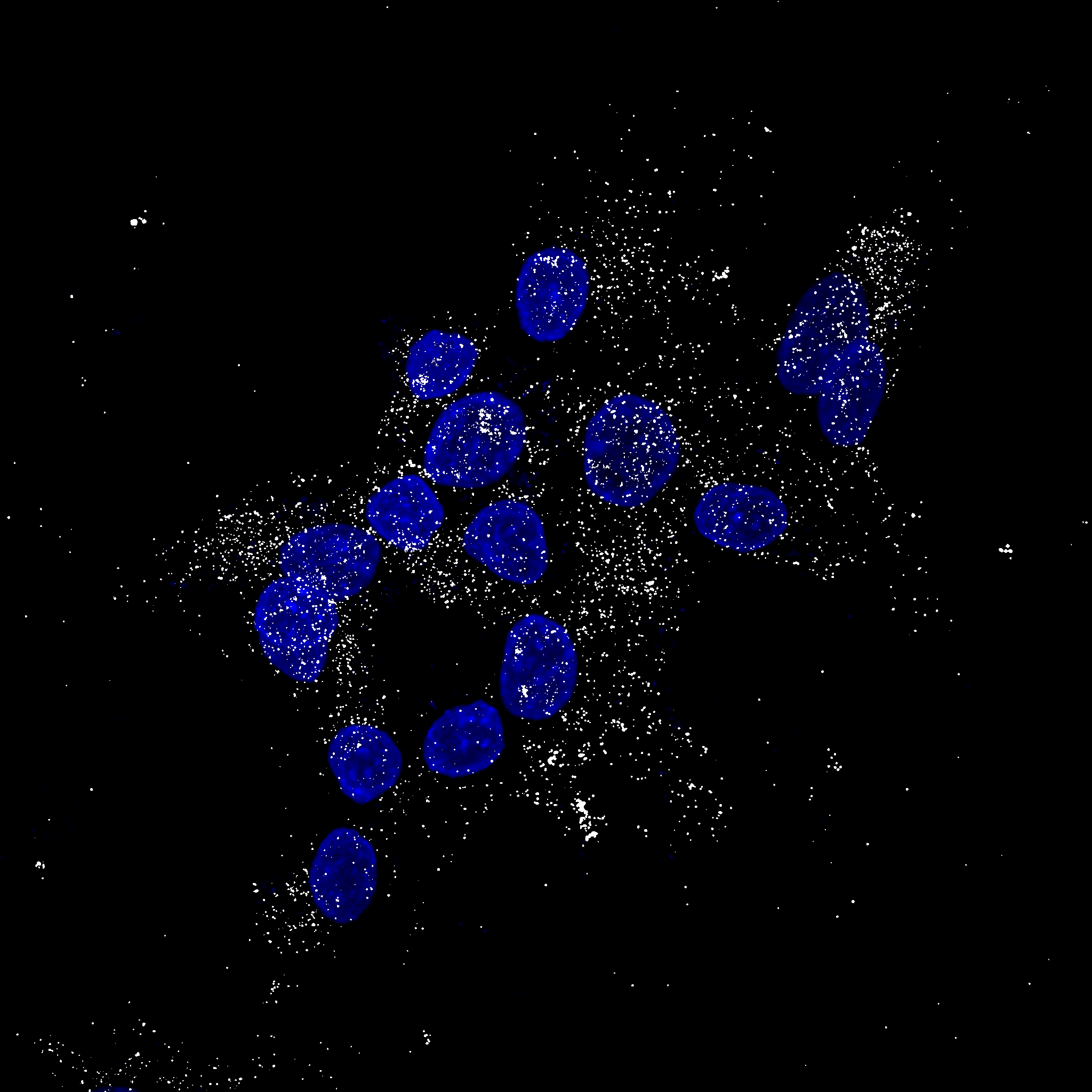

Supplement: Supplementary file 3 — Source data Fig. 2 [file 44319_2025_581_MOESM3_ESM.zip › 2 H/Bradykinin Top.tif]

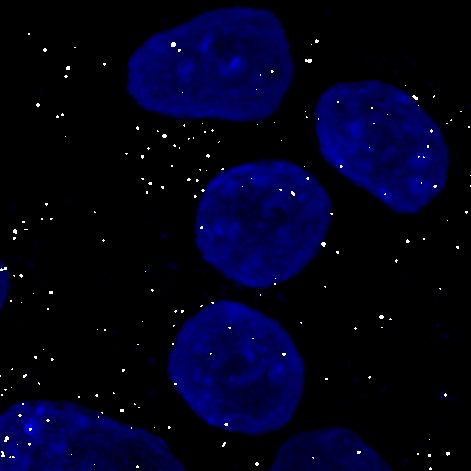

Supplement: Supplementary file 3 — Source data Fig. 2 [file 44319_2025_581_MOESM3_ESM.zip › 2 H/ML141 Bottom.tif]

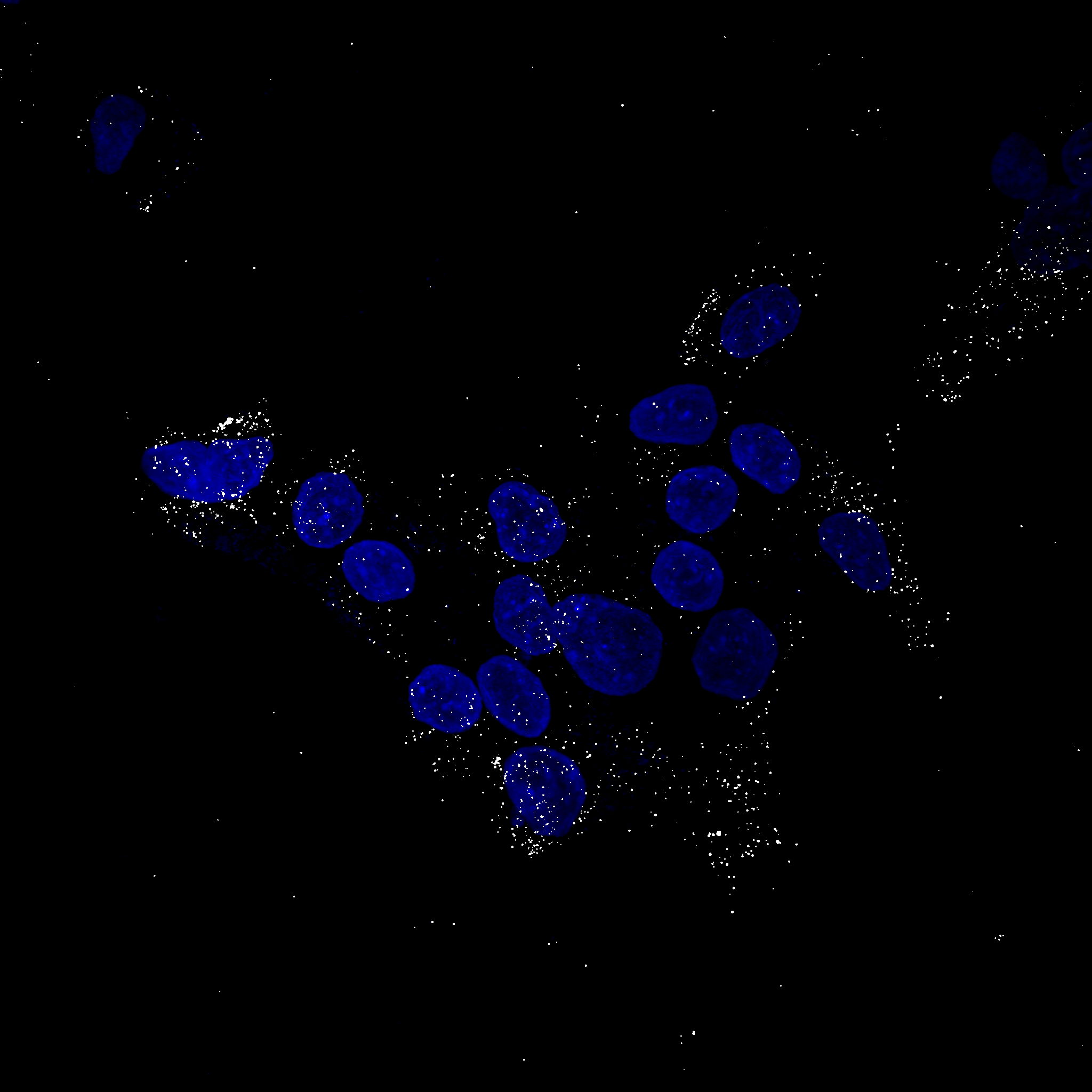

Supplement: Supplementary file 3 — Source data Fig. 2 [file 44319_2025_581_MOESM3_ESM.zip › 2 H/ML141 Top.tif]

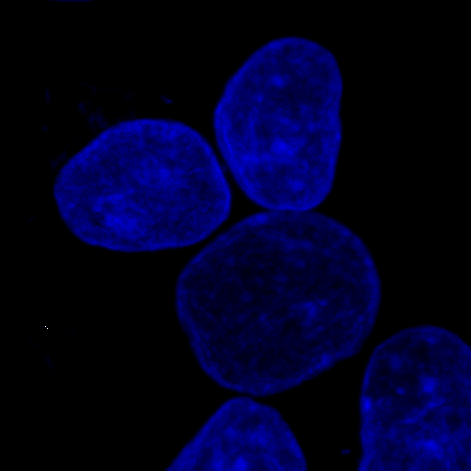

Supplement: Supplementary file 3 — Source data Fig. 2 [file 44319_2025_581_MOESM3_ESM.zip › 2 H/Noninfected Bottom.tif]

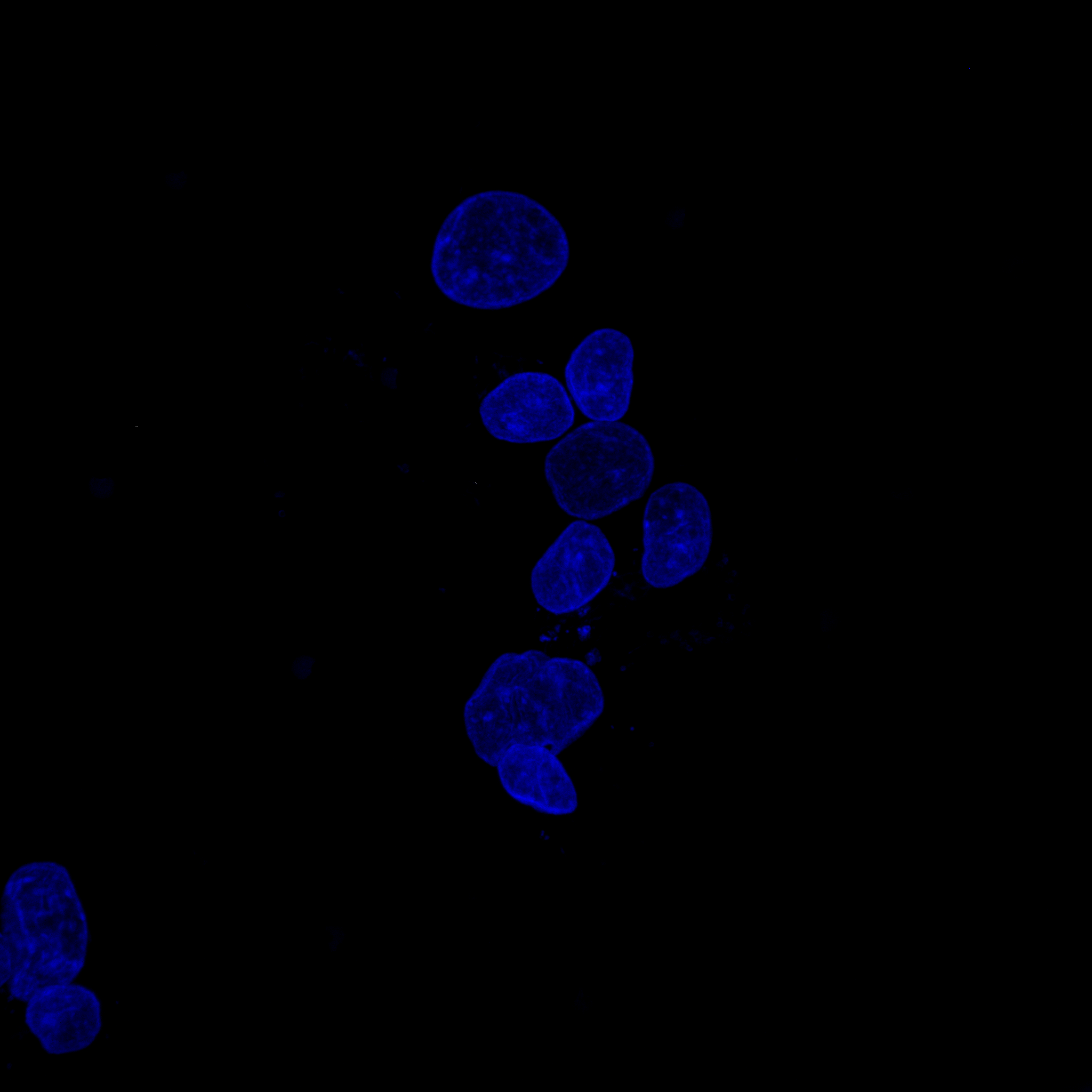

Supplement: Supplementary file 3 — Source data Fig. 2 [file 44319_2025_581_MOESM3_ESM.zip › 2 H/Noninfected Top.tif]

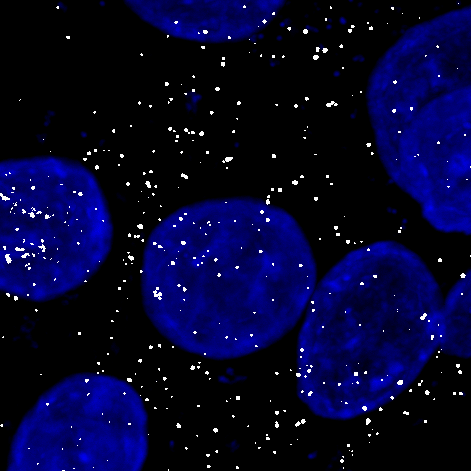

Supplement: Supplementary file 3 — Source data Fig. 2 [file 44319_2025_581_MOESM3_ESM.zip › 2 H/Untreated Bottom.tif]

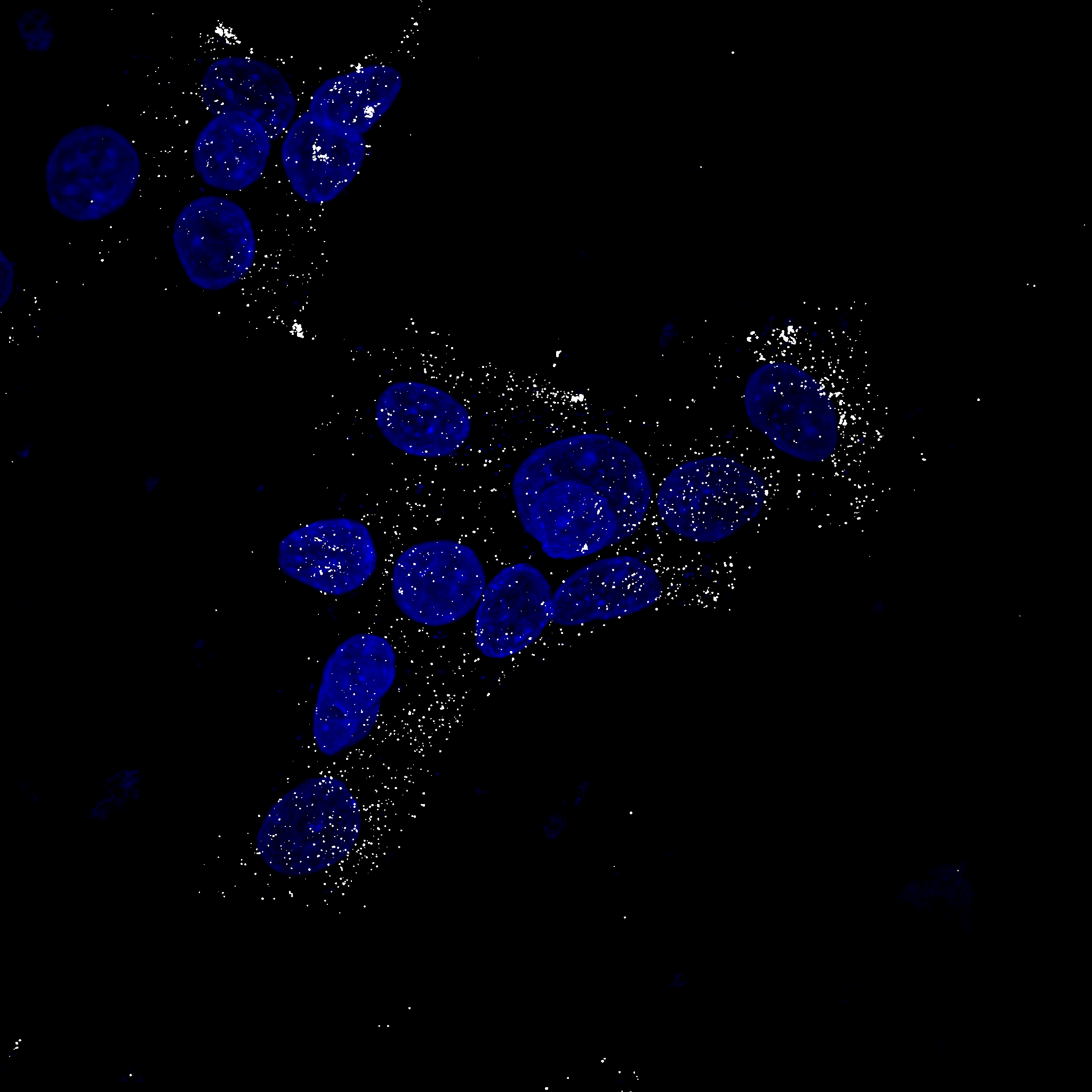

Supplement: Supplementary file 3 — Source data Fig. 2 [file 44319_2025_581_MOESM3_ESM.zip › 2 H/Untreated Top.tif]

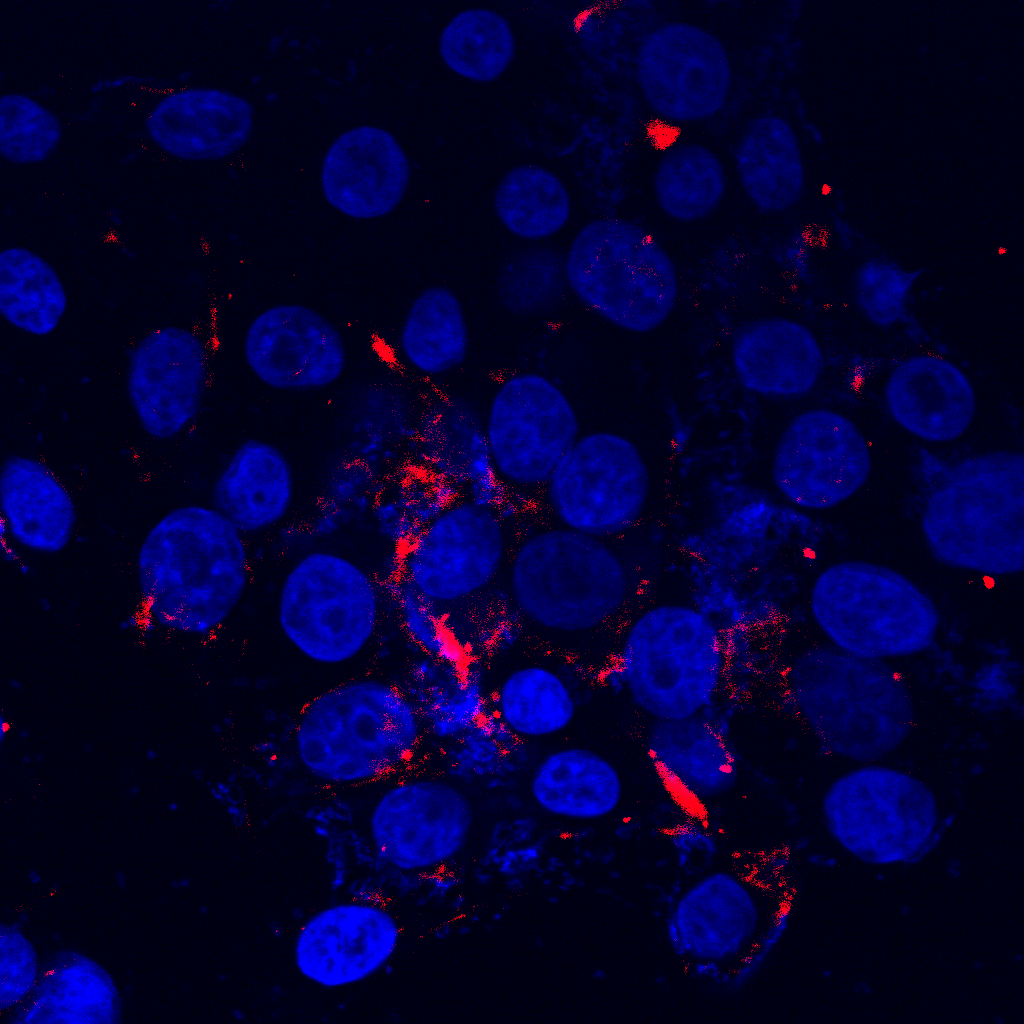

Supplement: Supplementary file 4 — Source data Fig. 3 [file 44319_2025_581_MOESM4_ESM.zip › 3 E/CA-1.tif]

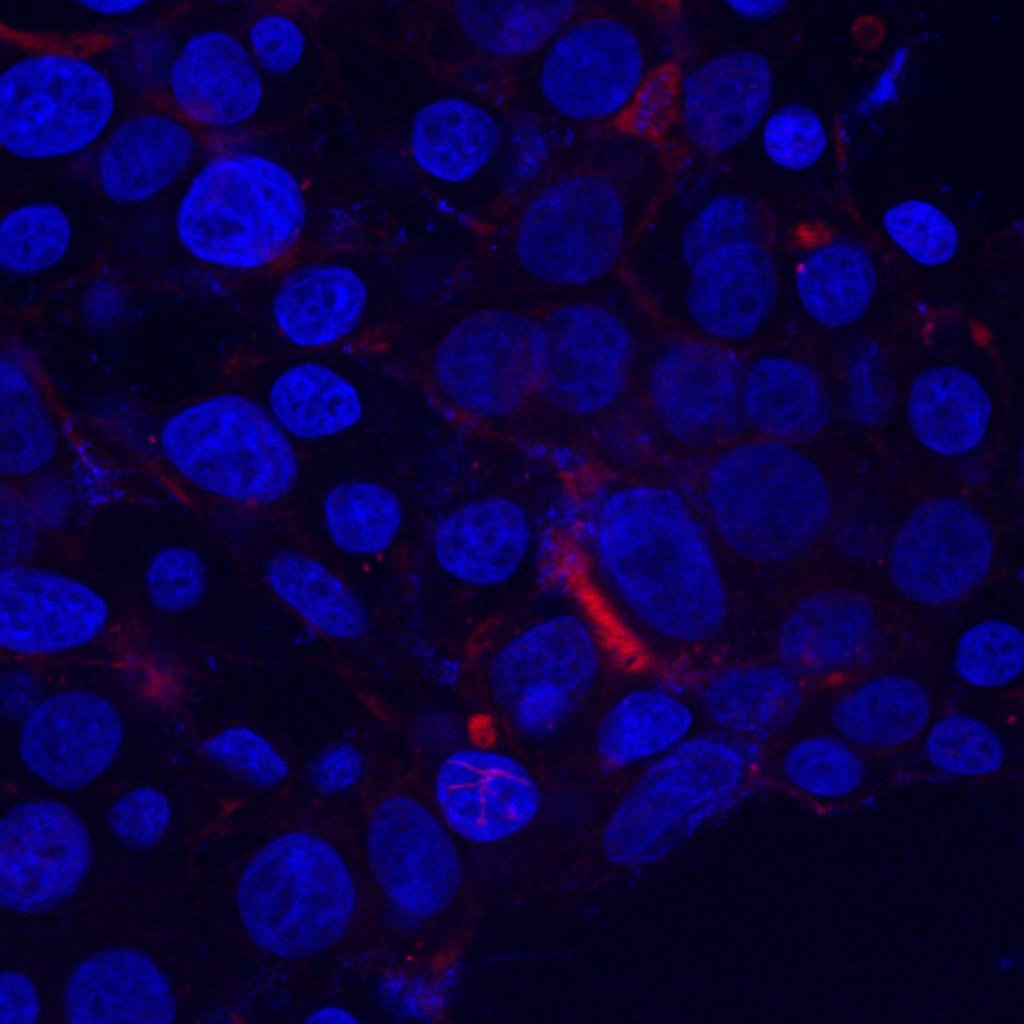

Supplement: Supplementary file 4 — Source data Fig. 3 [file 44319_2025_581_MOESM4_ESM.zip › 3 E/DN-1.jpg]

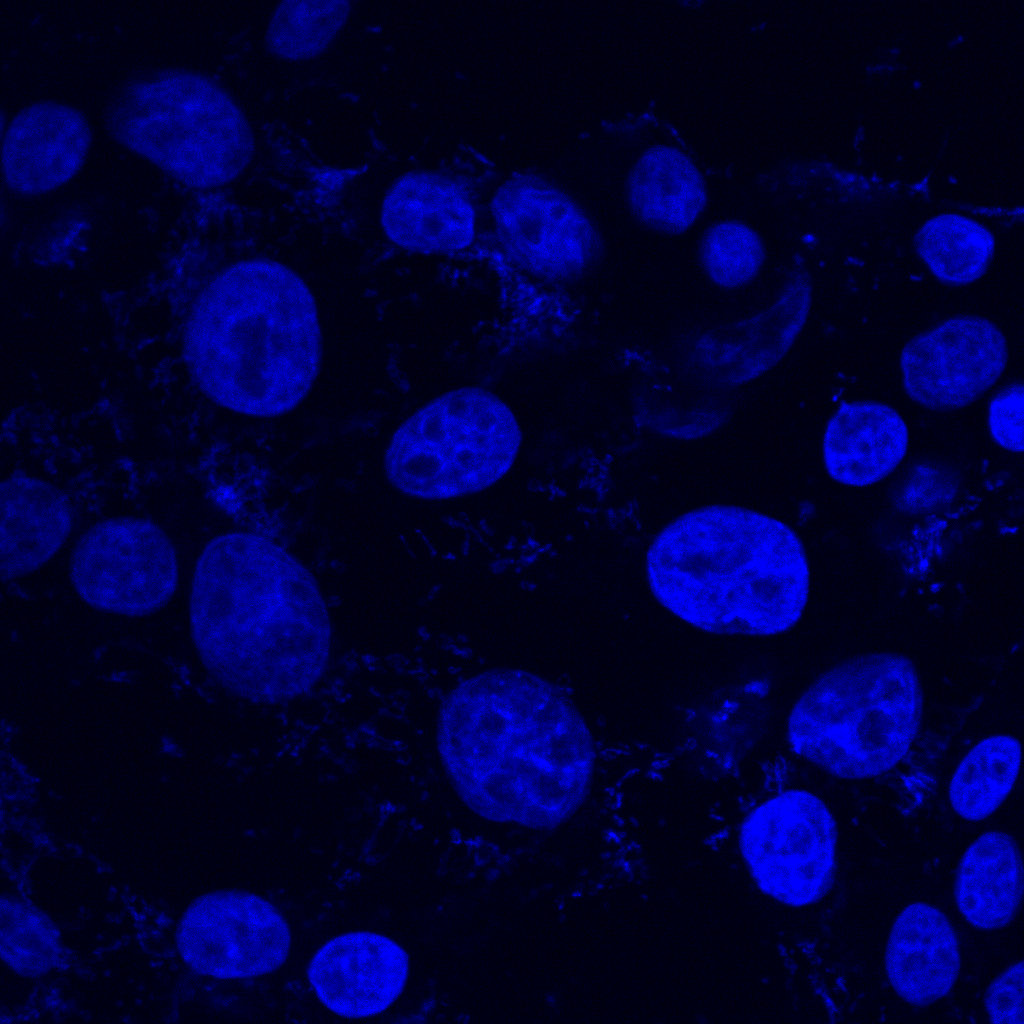

Supplement: Supplementary file 4 — Source data Fig. 3 [file 44319_2025_581_MOESM4_ESM.zip › 3 E/MOCK-1.tif]

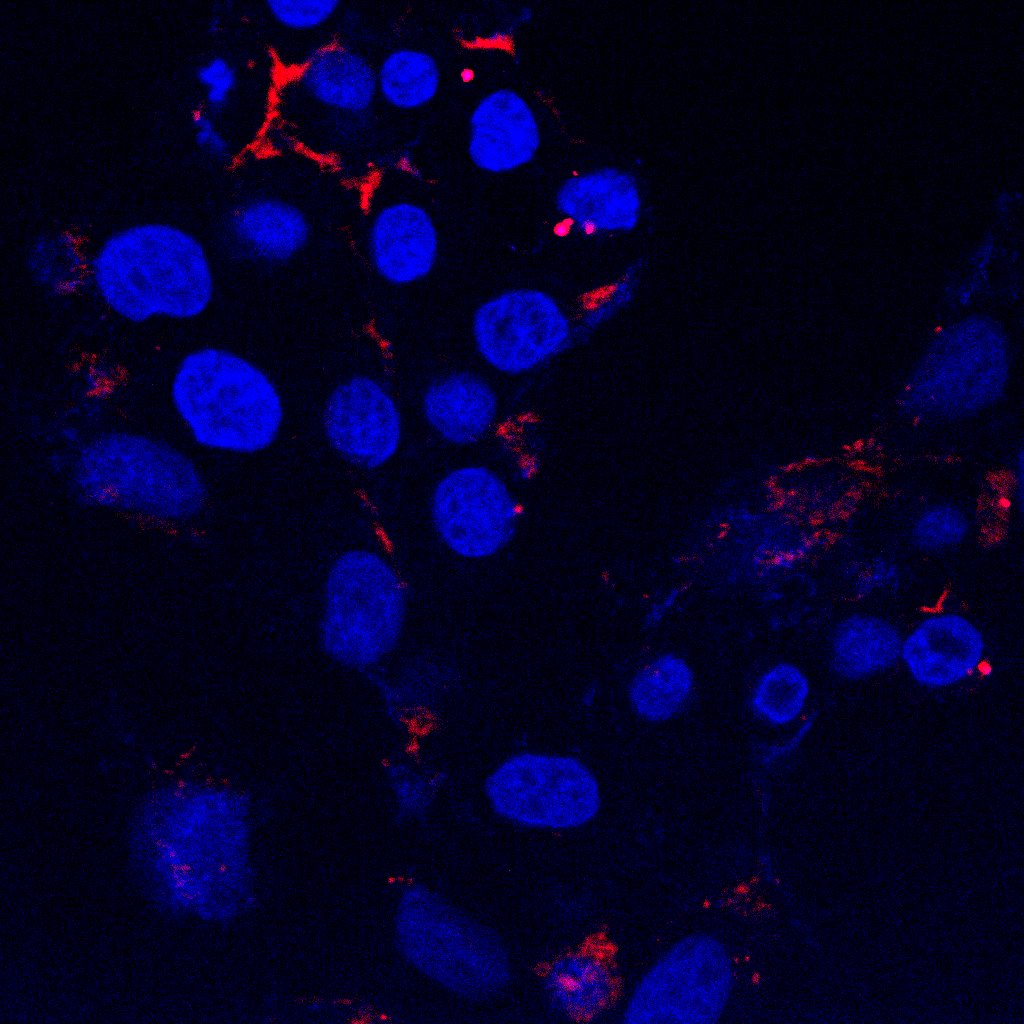

Supplement: Supplementary file 4 — Source data Fig. 3 [file 44319_2025_581_MOESM4_ESM.zip › 3 E/Vector-1.jpg]

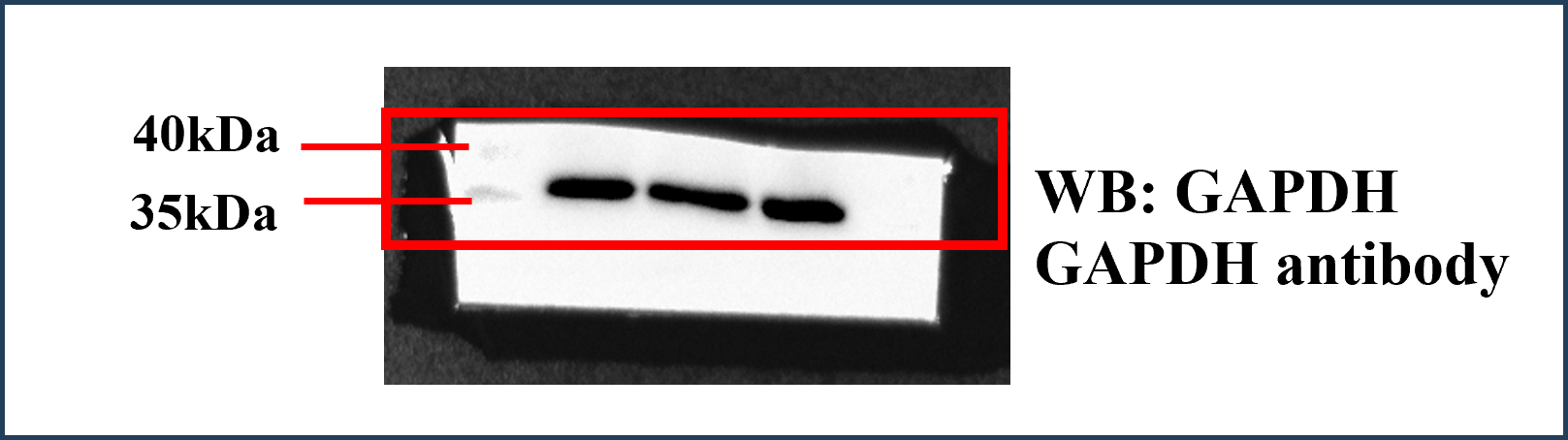

Supplement: Supplementary file 5 — Source data Fig. 4 [file 44319_2025_581_MOESM5_ESM.zip › 4 A/GAPDH.tif]

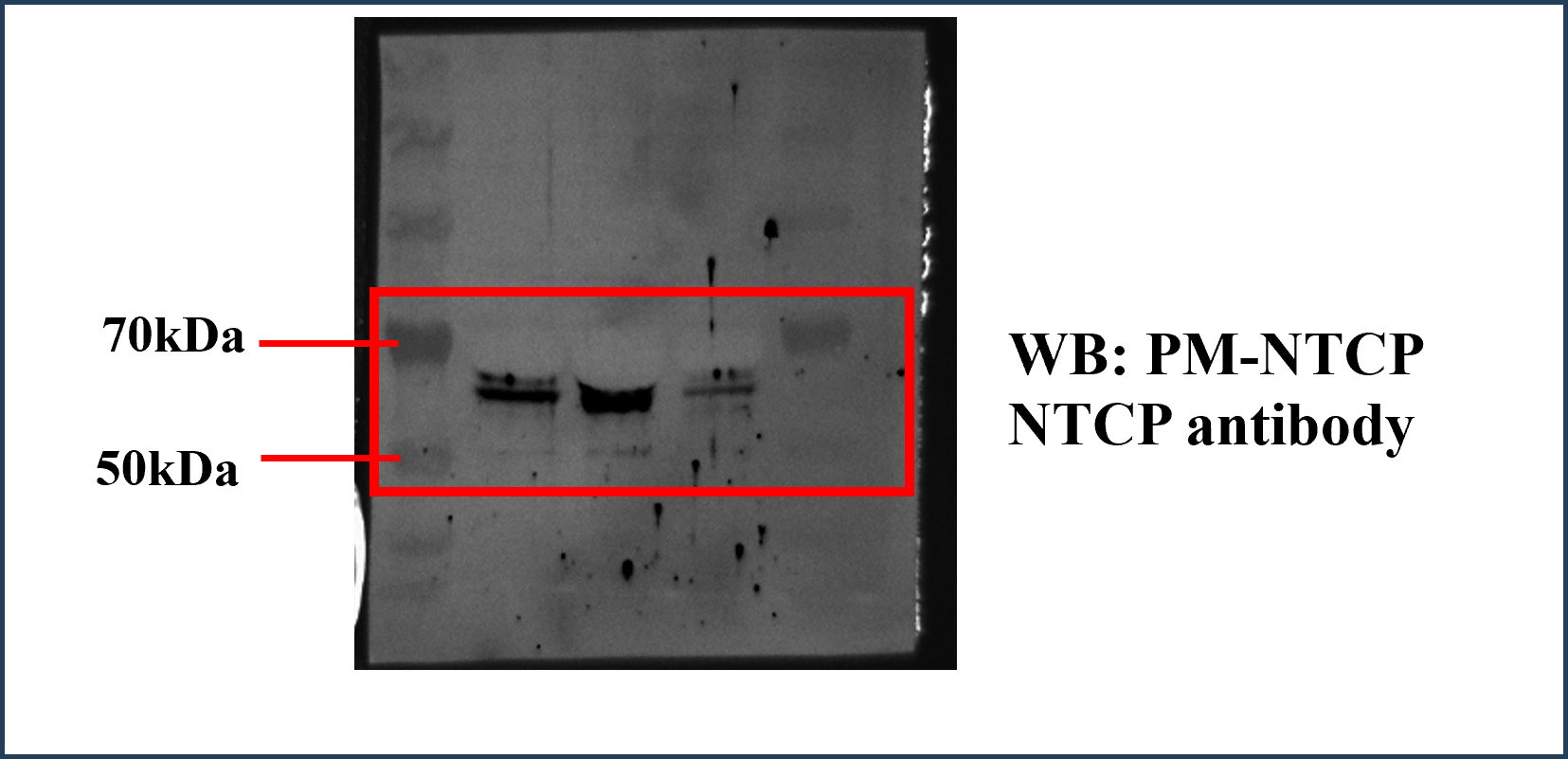

Supplement: Supplementary file 5 — Source data Fig. 4 [file 44319_2025_581_MOESM5_ESM.zip › 4 A/PM-NTCP.tif]

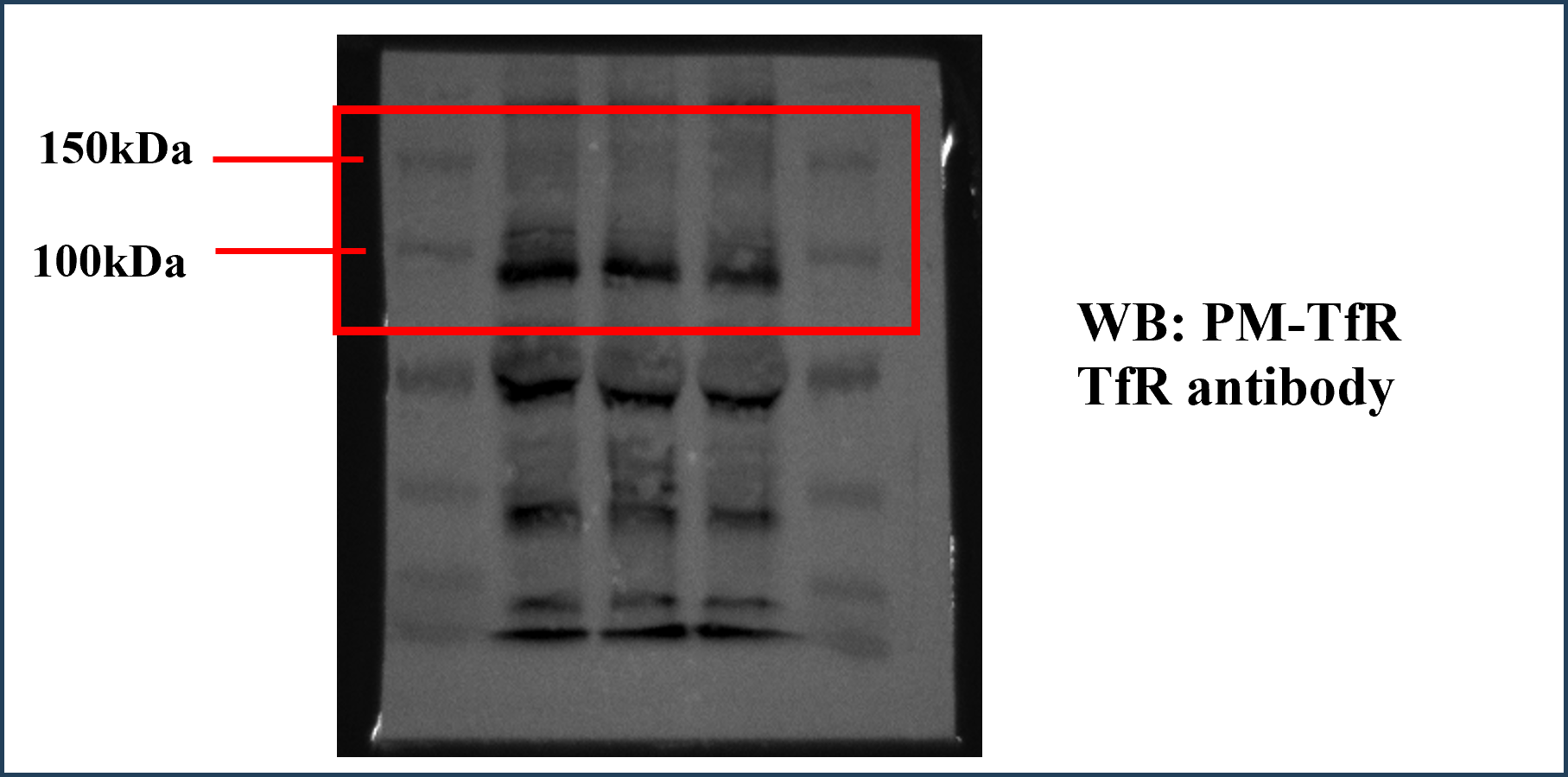

Supplement: Supplementary file 5 — Source data Fig. 4 [file 44319_2025_581_MOESM5_ESM.zip › 4 A/PM-TfR.tif]

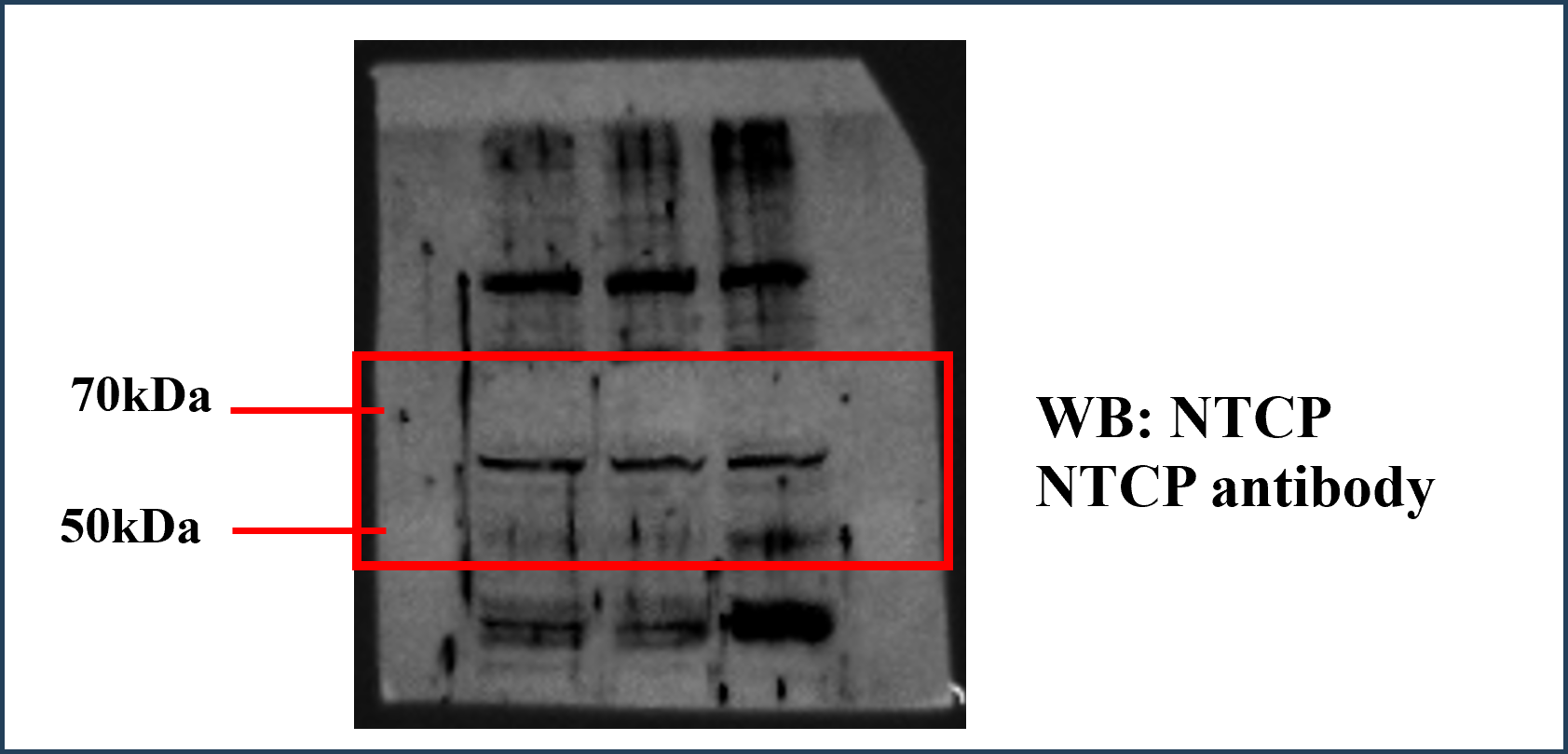

Supplement: Supplementary file 5 — Source data Fig. 4 [file 44319_2025_581_MOESM5_ESM.zip › 4 A/total NTCP.tif]

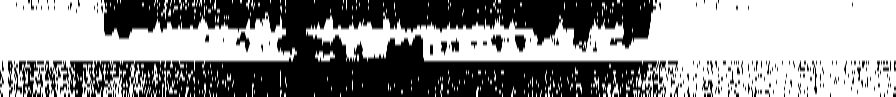

Supplement: Supplementary file 5 — Source data Fig. 4 [file 44319_2025_581_MOESM5_ESM.zip › 4 B/Cdc42 DN Bottom.tif]

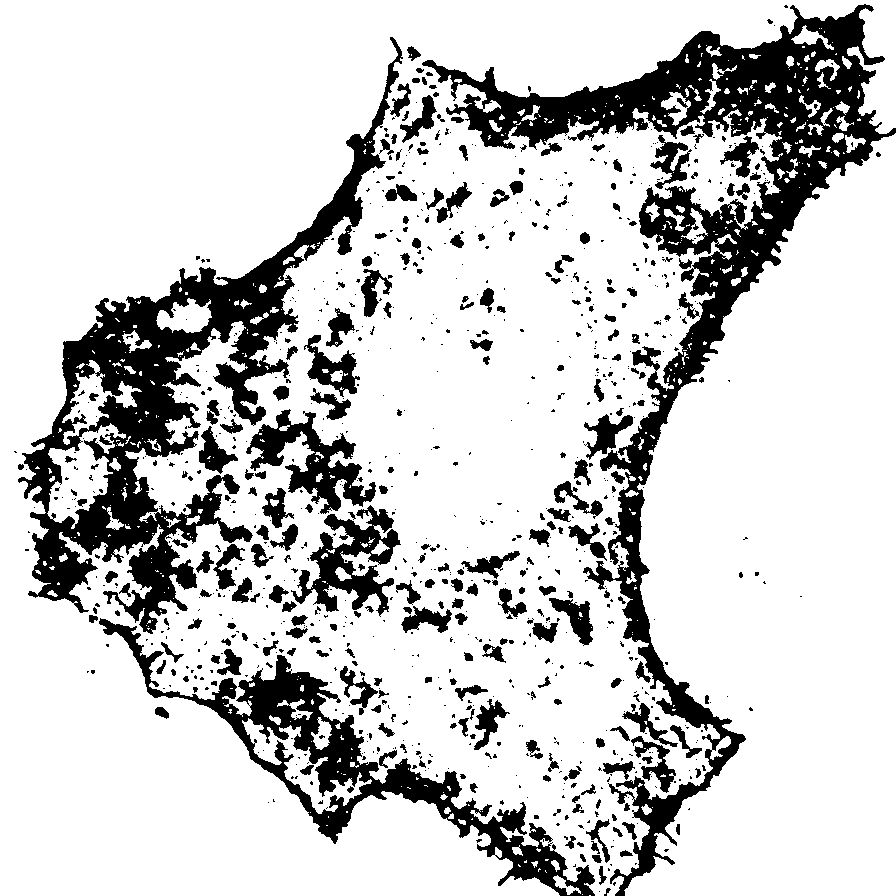

Supplement: Supplementary file 5 — Source data Fig. 4 [file 44319_2025_581_MOESM5_ESM.zip › 4 B/Cdc42 DN Top.tif]

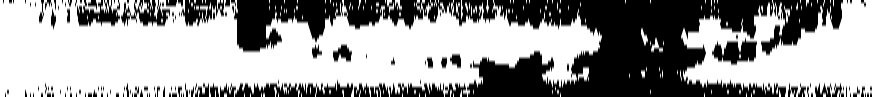

Supplement: Supplementary file 5 — Source data Fig. 4 [file 44319_2025_581_MOESM5_ESM.zip › 4 B/Cdc42 WT Bottom.tif]

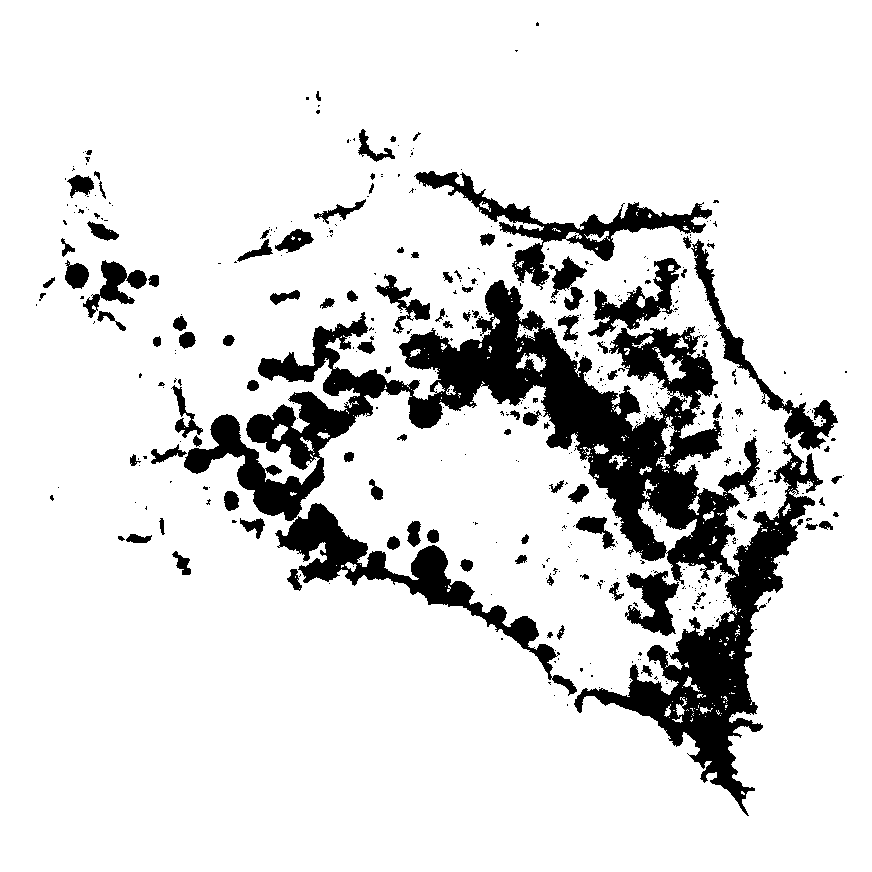

Supplement: Supplementary file 5 — Source data Fig. 4 [file 44319_2025_581_MOESM5_ESM.zip › 4 B/Cdc42 WT Top.tif]

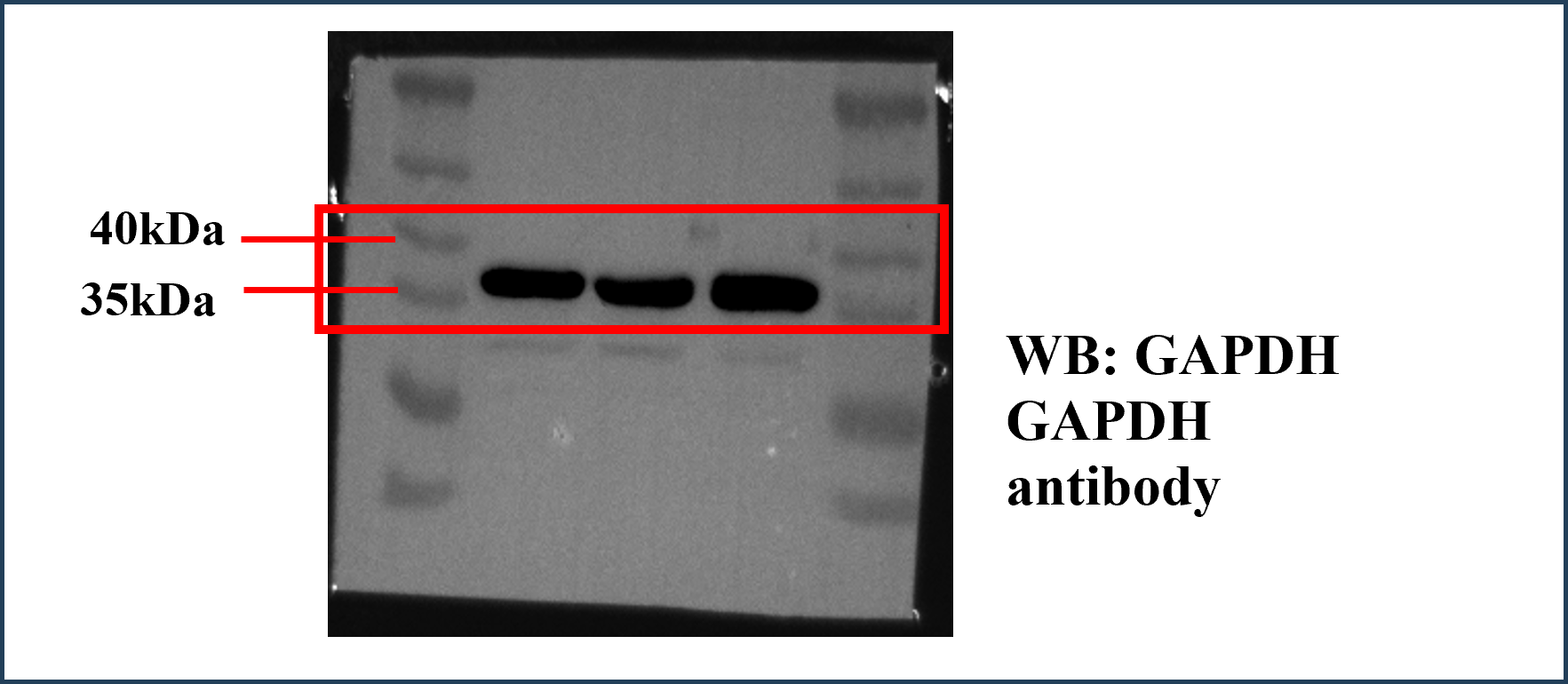

Supplement: Supplementary file 5 — Source data Fig. 4 [file 44319_2025_581_MOESM5_ESM.zip › 4 C/GAPDH.tif]

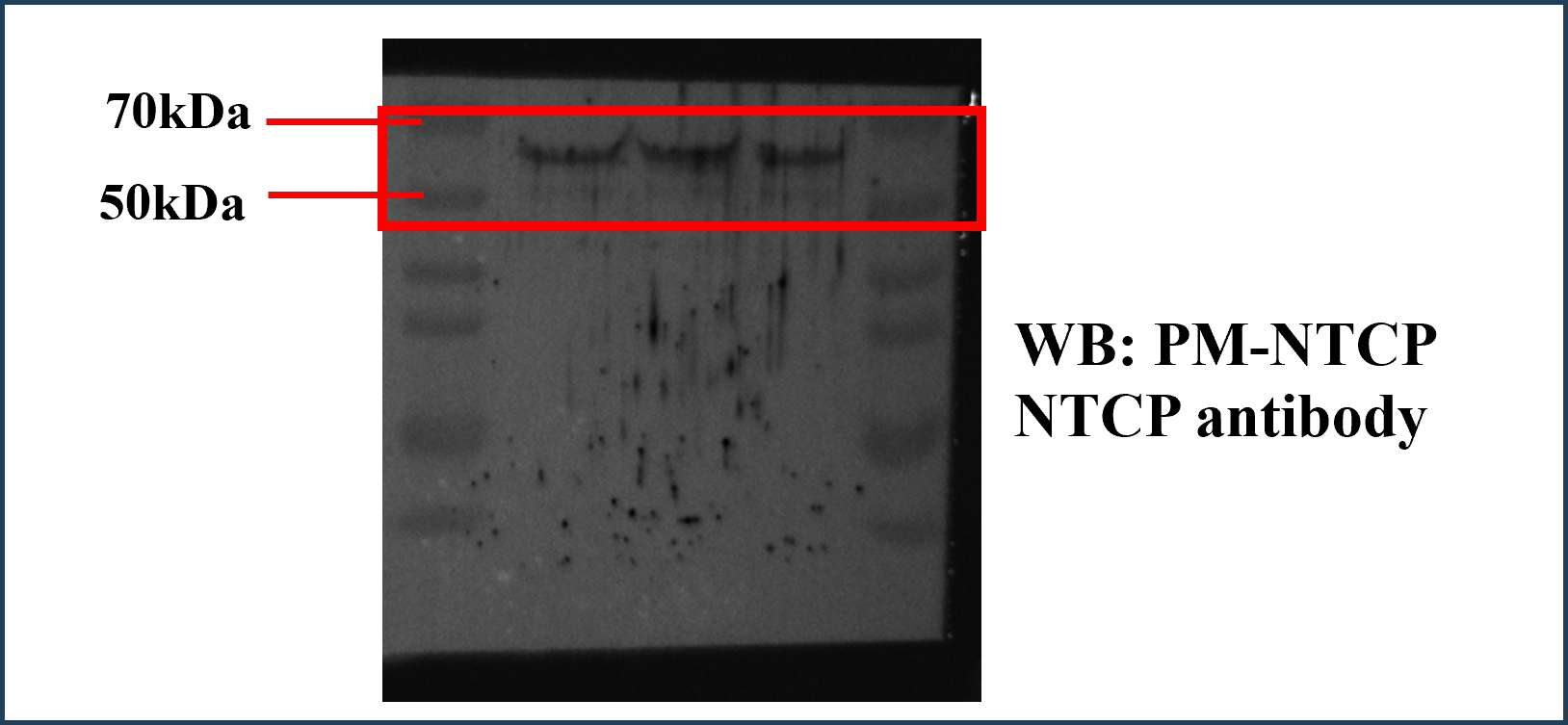

Supplement: Supplementary file 5 — Source data Fig. 4 [file 44319_2025_581_MOESM5_ESM.zip › 4 C/PM-NTCP.tif]

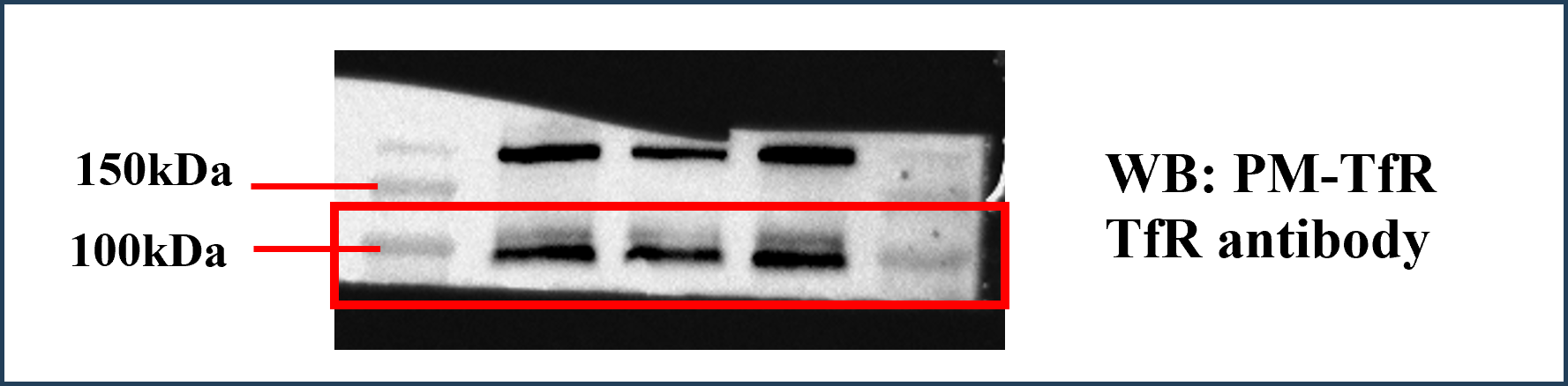

Supplement: Supplementary file 5 — Source data Fig. 4 [file 44319_2025_581_MOESM5_ESM.zip › 4 C/PM-TfR.tif]

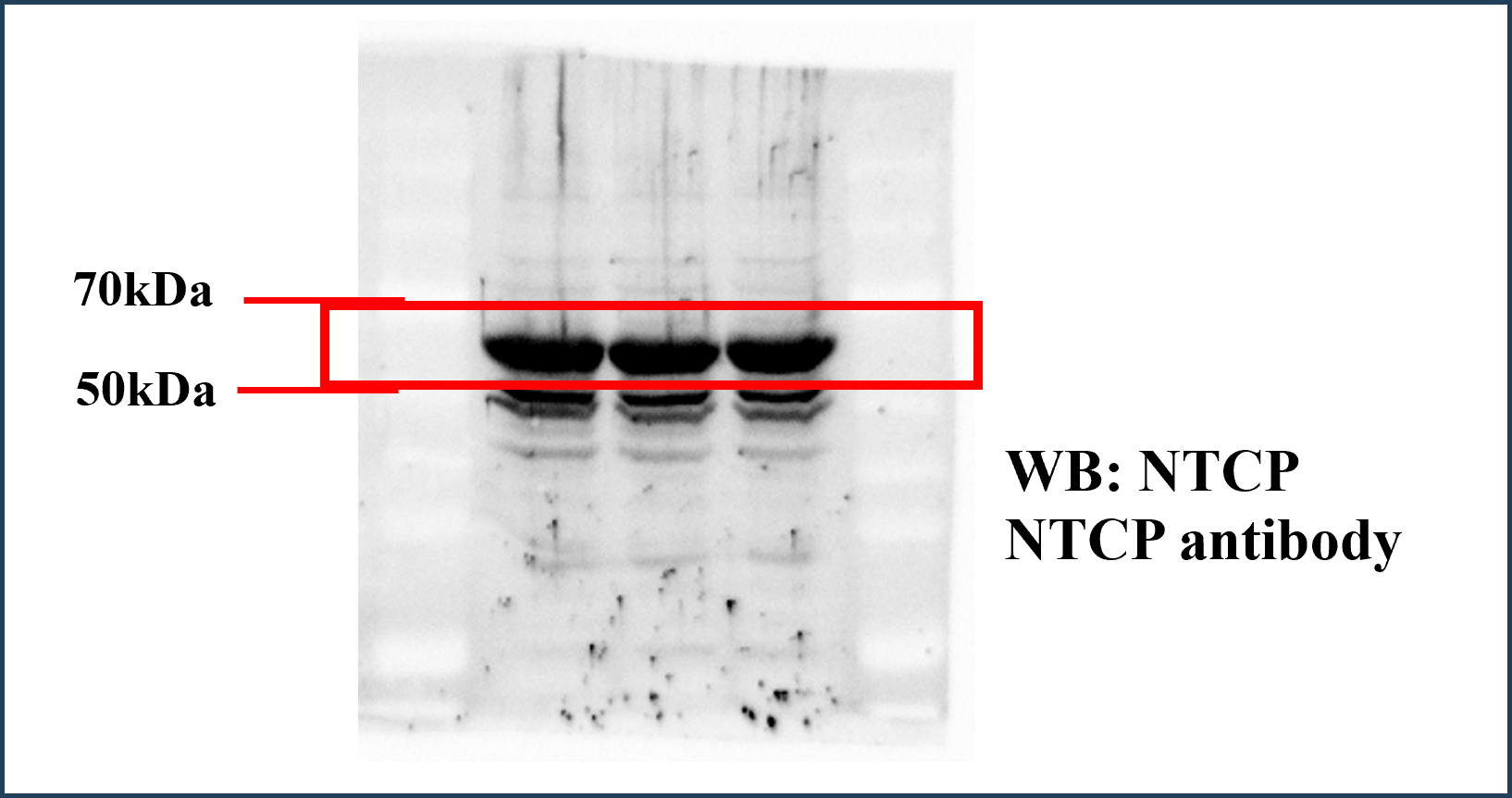

Supplement: Supplementary file 5 — Source data Fig. 4 [file 44319_2025_581_MOESM5_ESM.zip › 4 C/total NTCP.tif]

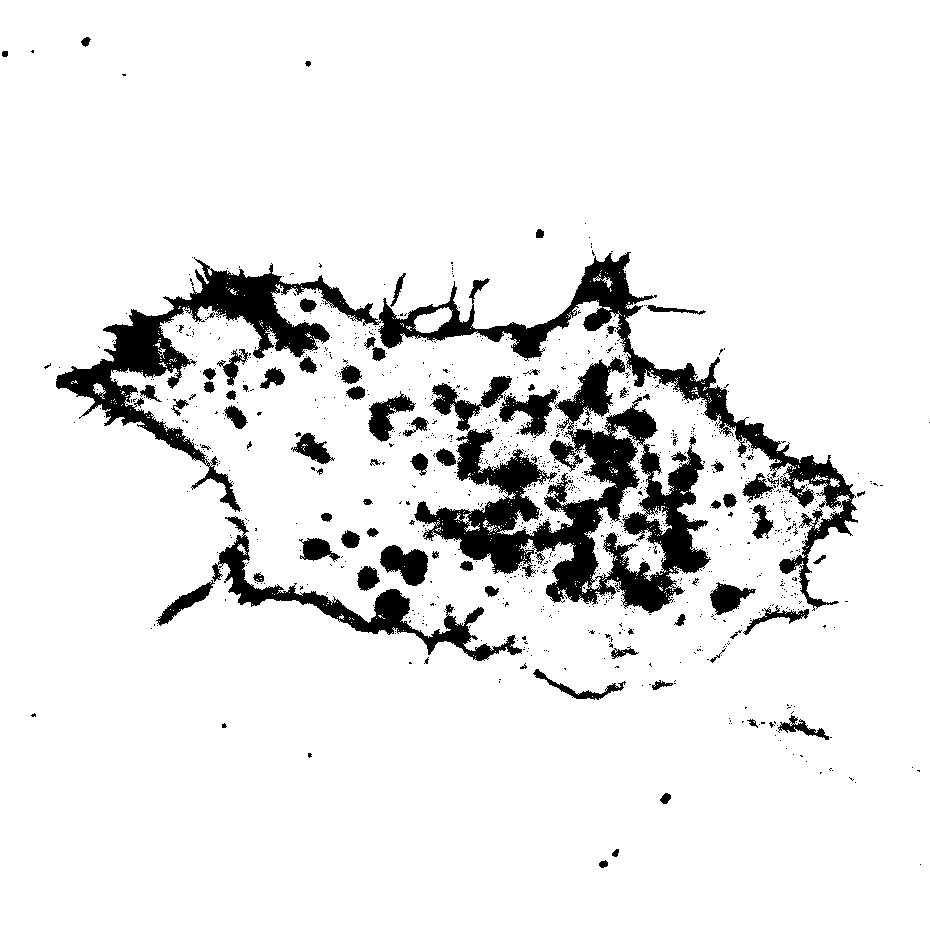

Supplement: Supplementary file 5 — Source data Fig. 4 [file 44319_2025_581_MOESM5_ESM.zip › 4 D/Untreated Top.tif]

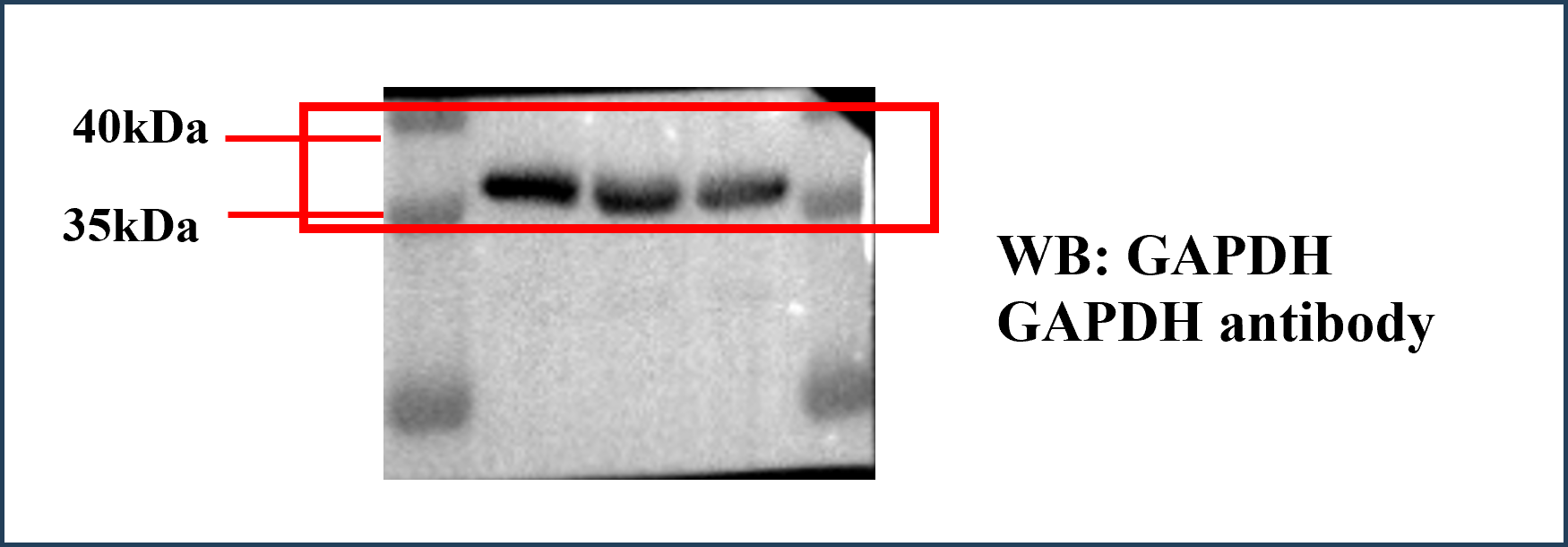

Supplement: Supplementary file 5 — Source data Fig. 4 [file 44319_2025_581_MOESM5_ESM.zip › 4 F/GAPDH.tif]

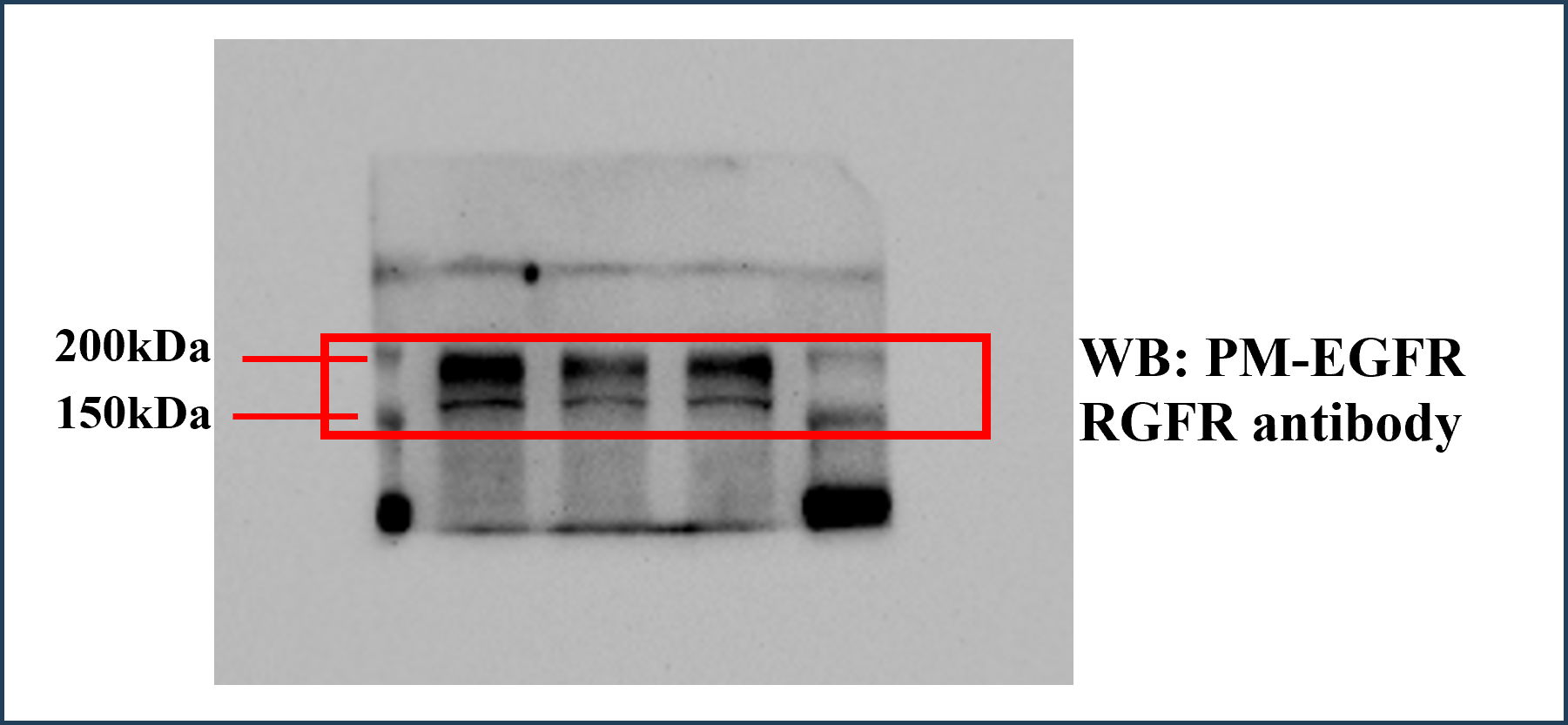

Supplement: Supplementary file 5 — Source data Fig. 4 [file 44319_2025_581_MOESM5_ESM.zip › 4 F/PM-EGFR.tif]

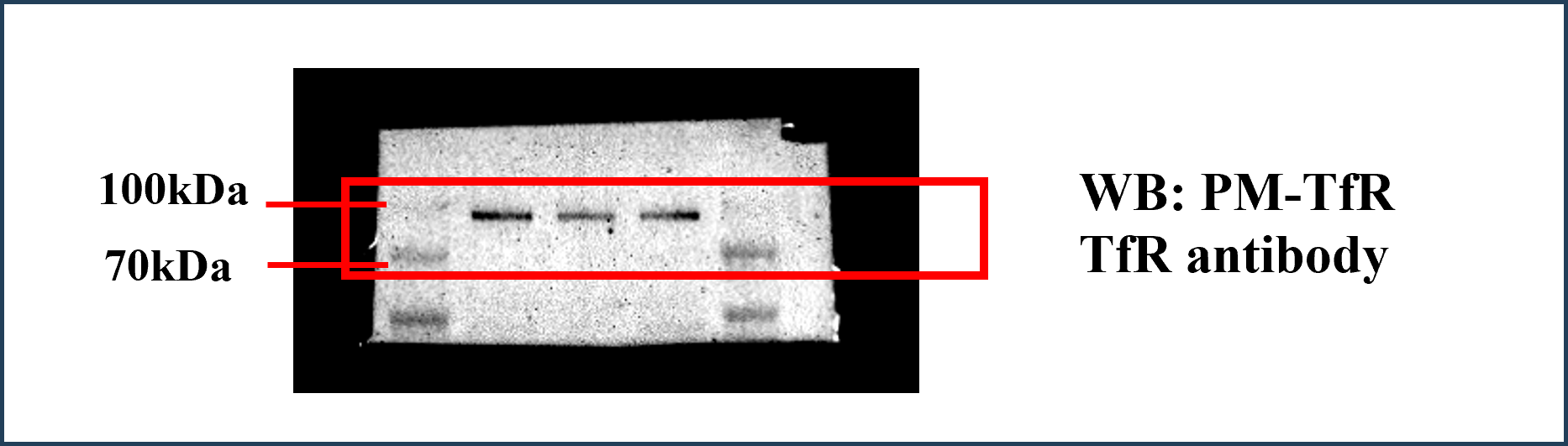

Supplement: Supplementary file 5 — Source data Fig. 4 [file 44319_2025_581_MOESM5_ESM.zip › 4 F/PM-TfR.tif]

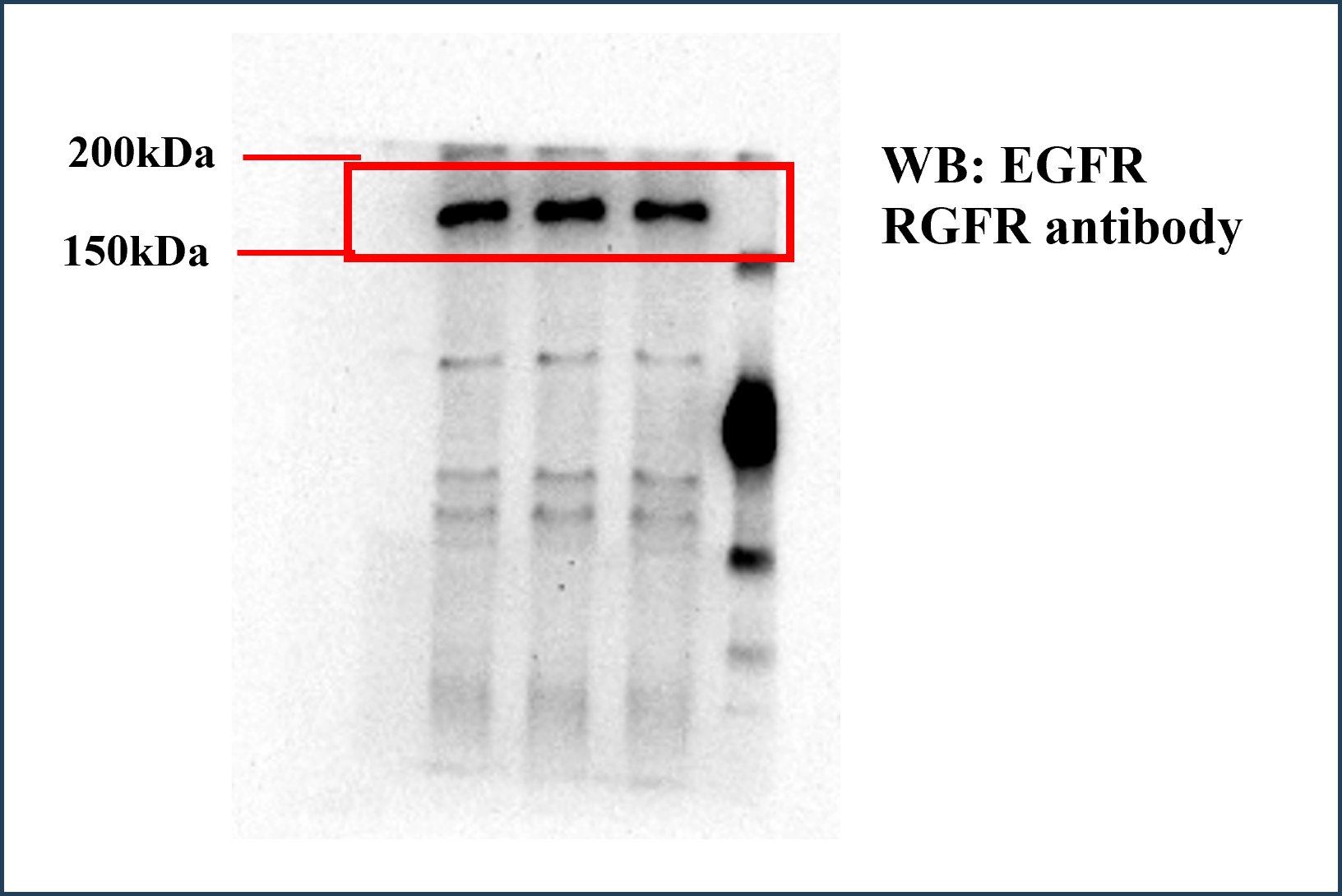

Supplement: Supplementary file 5 — Source data Fig. 4 [file 44319_2025_581_MOESM5_ESM.zip › 4 F/total EGFR.tif]

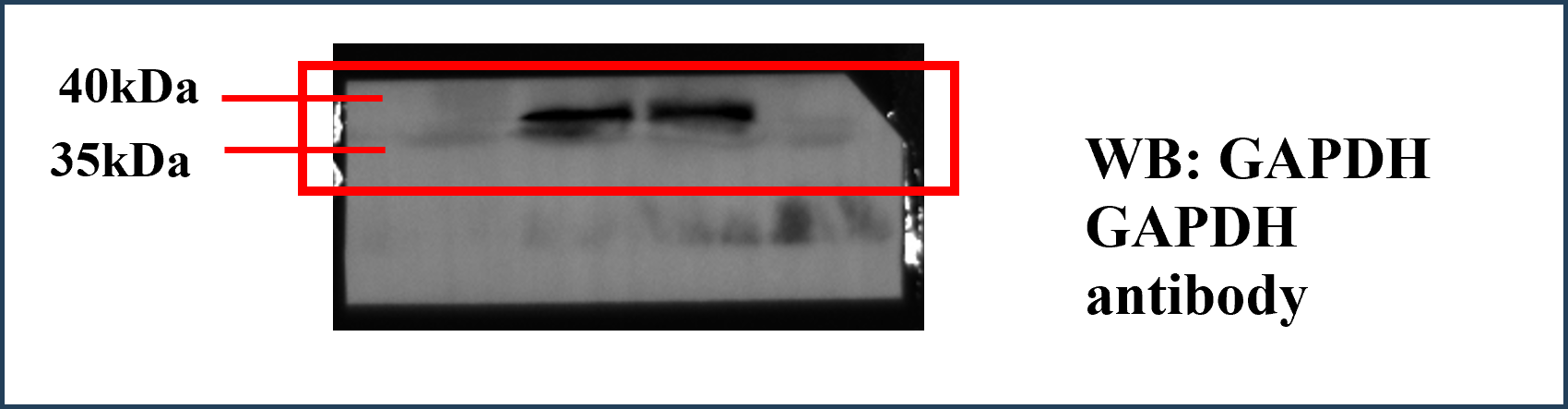

Supplement: Supplementary file 6 — Source data Fig. 5 [file 44319_2025_581_MOESM6_ESM.zip › 5 A/GAPDH.tif]

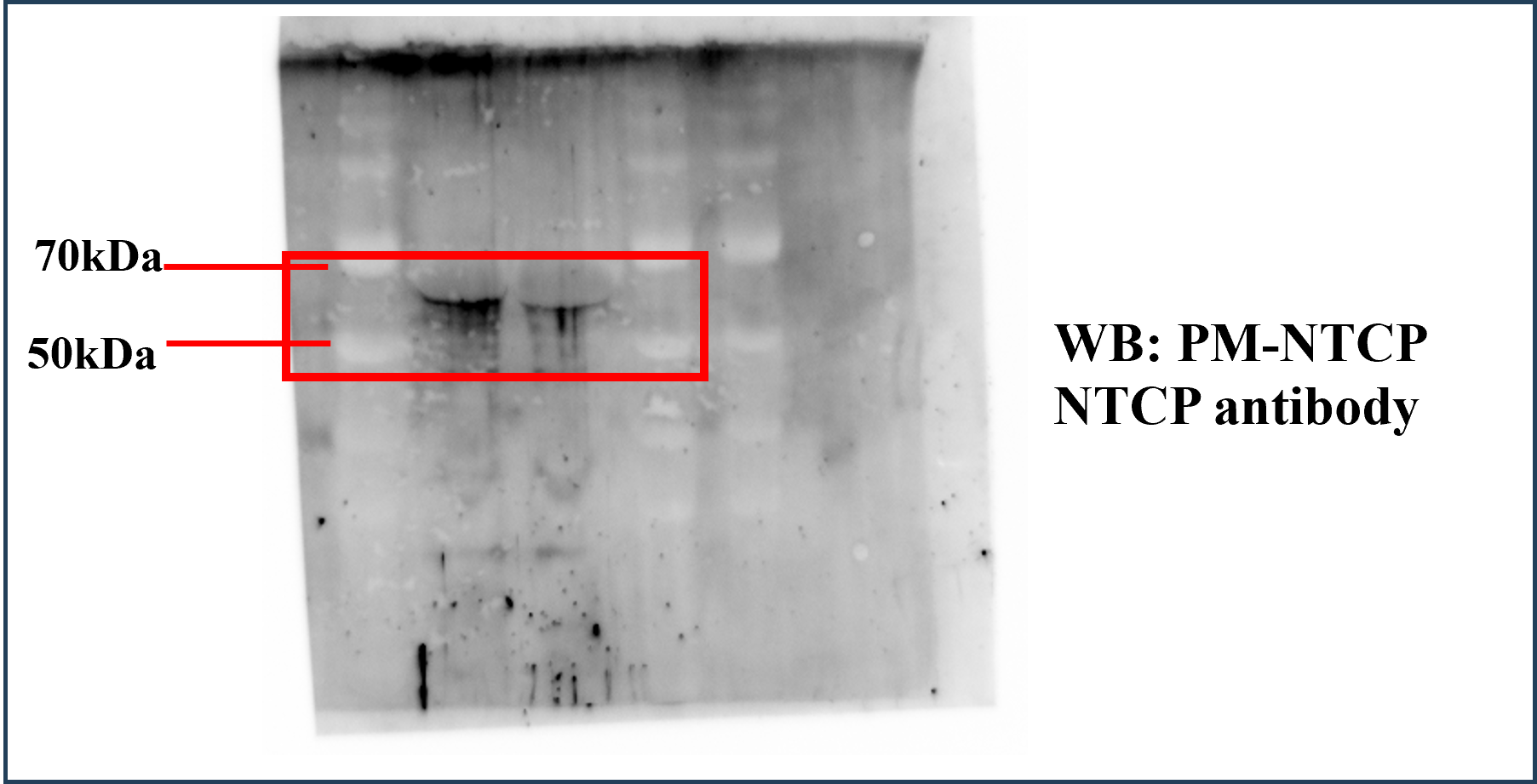

Supplement: Supplementary file 6 — Source data Fig. 5 [file 44319_2025_581_MOESM6_ESM.zip › 5 A/PM-NTCP.tif]

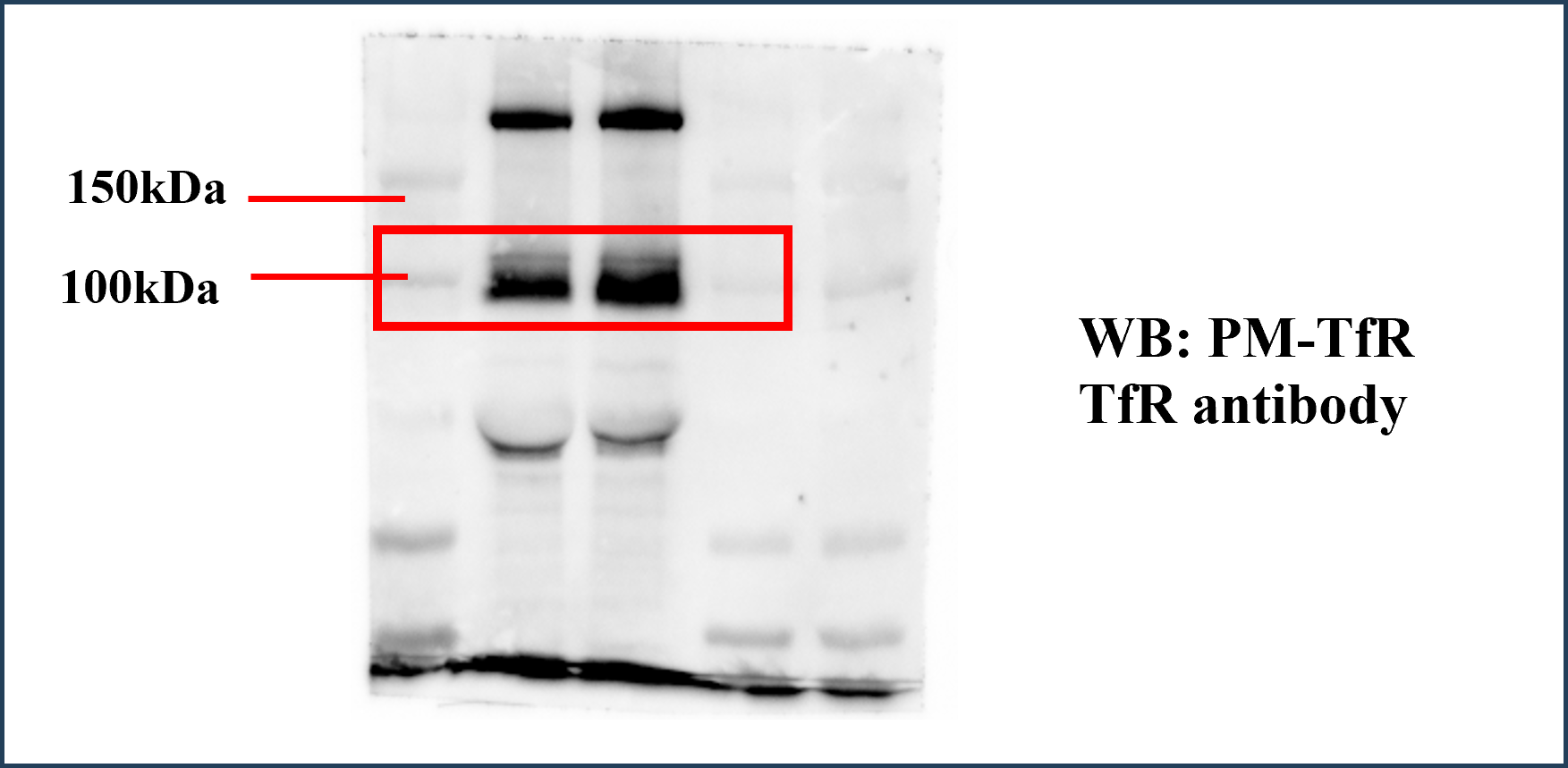

Supplement: Supplementary file 6 — Source data Fig. 5 [file 44319_2025_581_MOESM6_ESM.zip › 5 A/PM-TfR.tif]

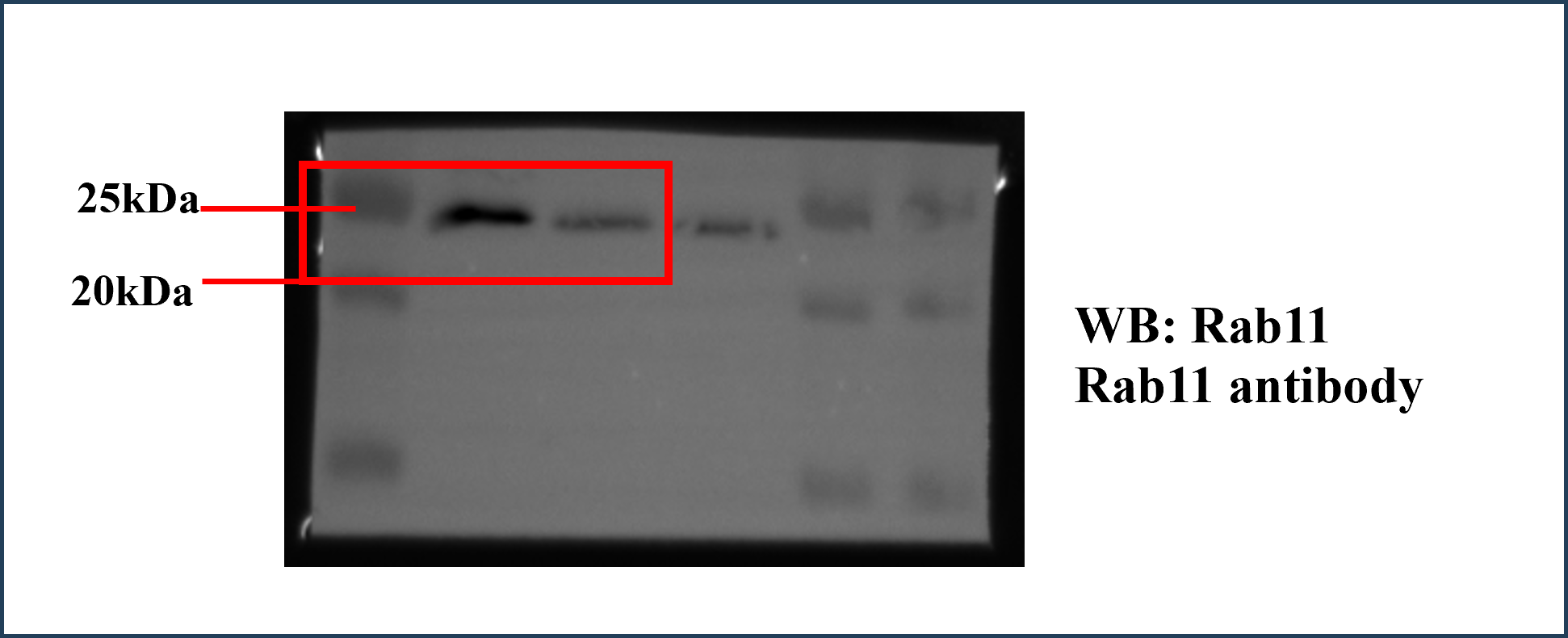

Supplement: Supplementary file 6 — Source data Fig. 5 [file 44319_2025_581_MOESM6_ESM.zip › 5 A/Rab11.tif]

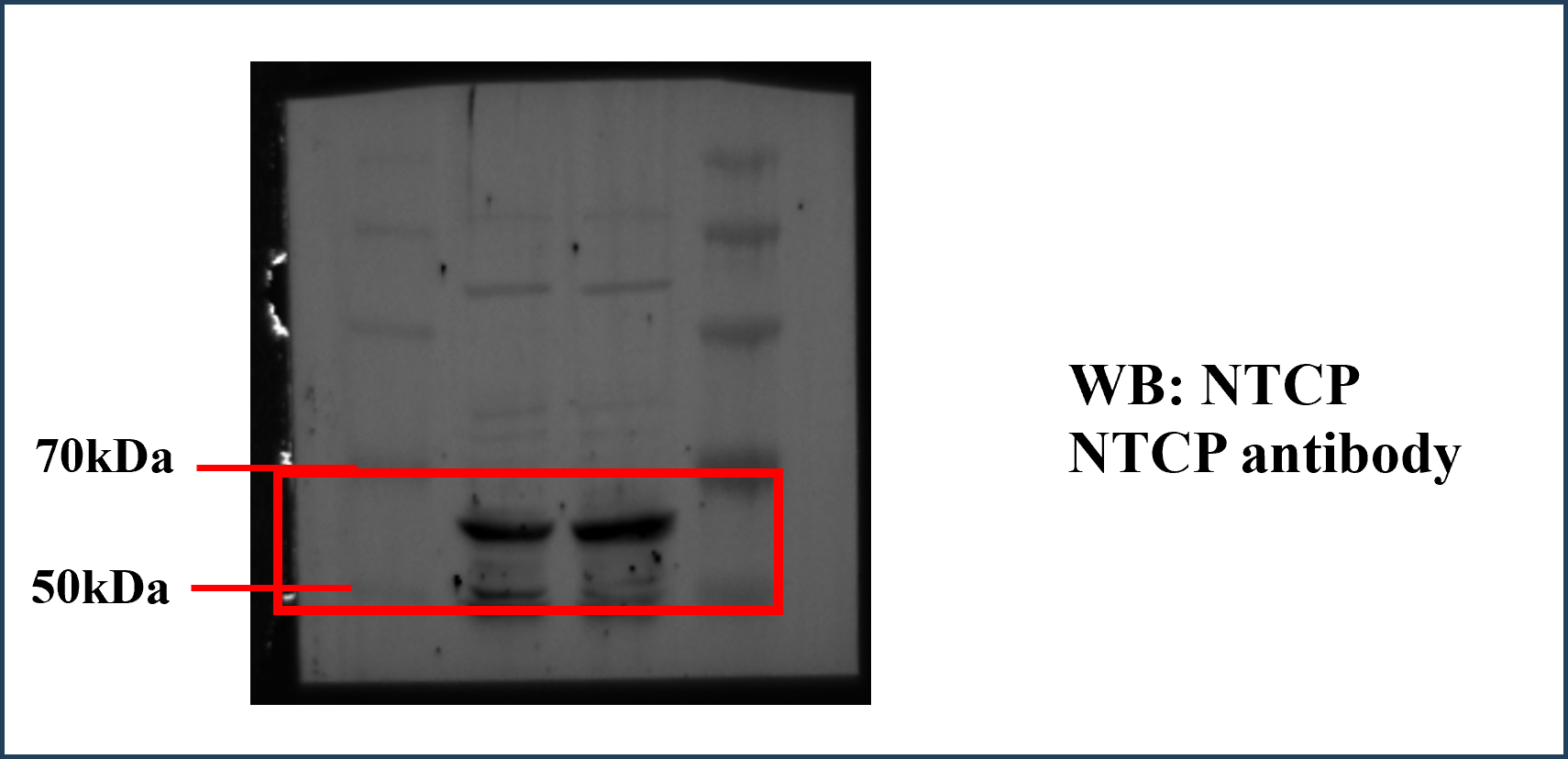

Supplement: Supplementary file 6 — Source data Fig. 5 [file 44319_2025_581_MOESM6_ESM.zip › 5 A/total NTCP.tif]

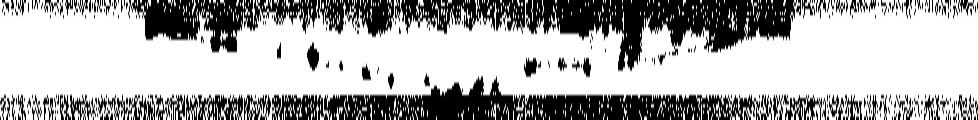

Supplement: Supplementary file 6 — Source data Fig. 5 [file 44319_2025_581_MOESM6_ESM.zip › 5 B/shRab11 Bottom.tif]

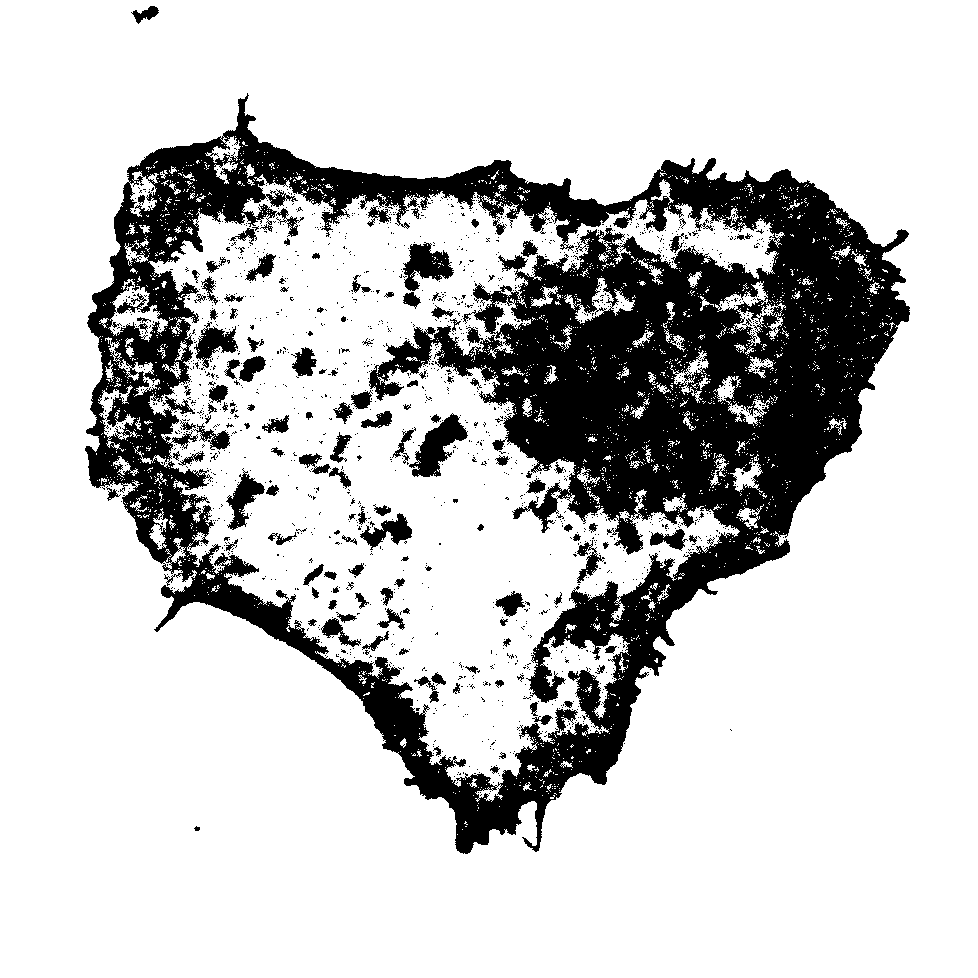

Supplement: Supplementary file 6 — Source data Fig. 5 [file 44319_2025_581_MOESM6_ESM.zip › 5 B/shRab11 Top.tif]

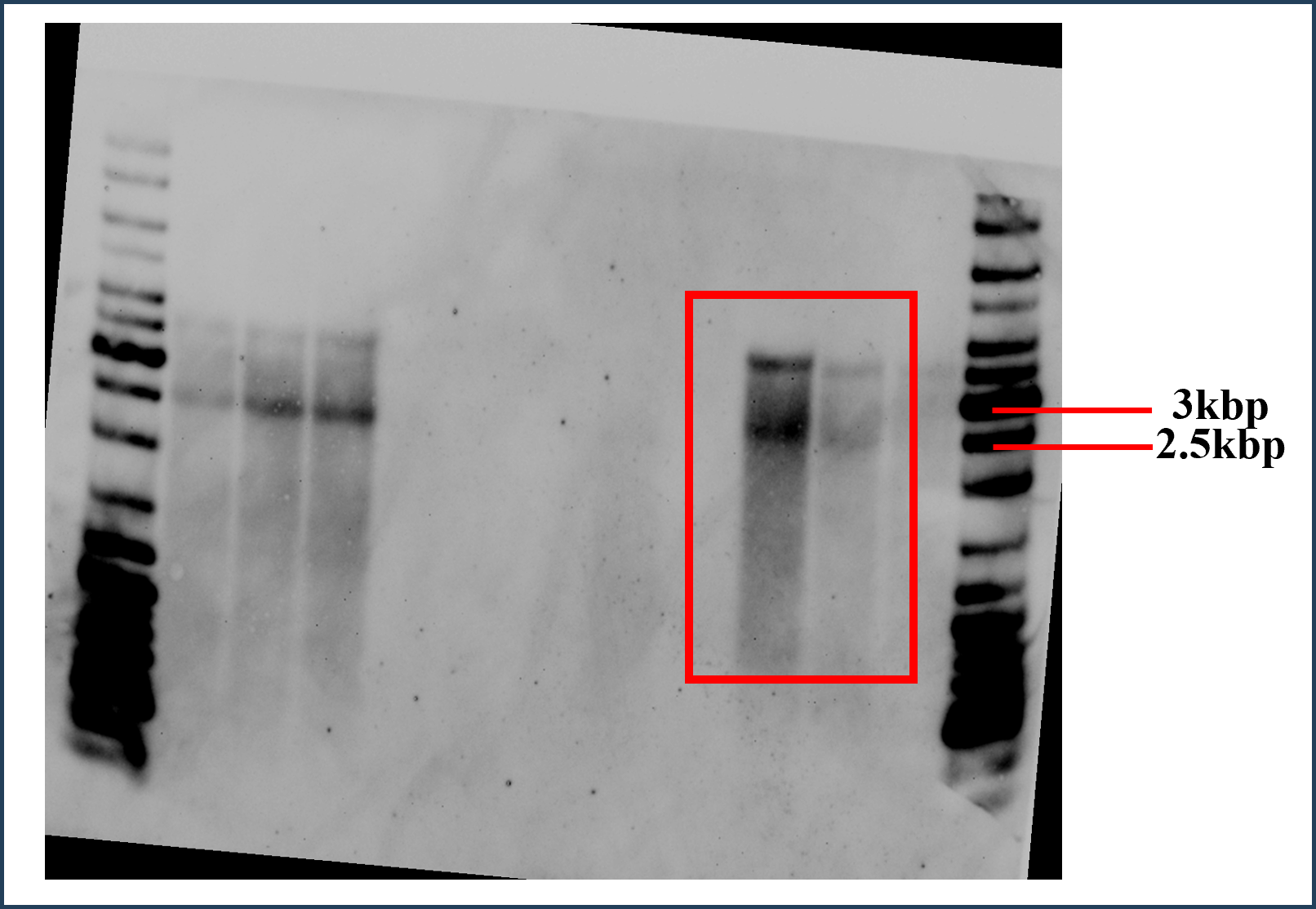

Supplement: Supplementary file 6 — Source data Fig. 5 [file 44319_2025_581_MOESM6_ESM.zip › 5 D/HBV DNA southern.tif]

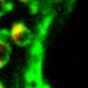

Supplement: Supplementary file 6 — Source data Fig. 5 [file 44319_2025_581_MOESM6_ESM.zip › 5 F/0s.tif]

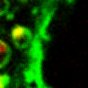

Supplement: Supplementary file 6 — Source data Fig. 5 [file 44319_2025_581_MOESM6_ESM.zip › 5 F/10s.tif]

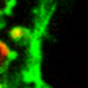

Supplement: Supplementary file 6 — Source data Fig. 5 [file 44319_2025_581_MOESM6_ESM.zip › 5 F/20s.tif]

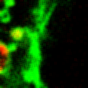

Supplement: Supplementary file 6 — Source data Fig. 5 [file 44319_2025_581_MOESM6_ESM.zip › 5 F/30s.tif]

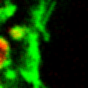

Supplement: Supplementary file 6 — Source data Fig. 5 [file 44319_2025_581_MOESM6_ESM.zip › 5 F/40s.tif]

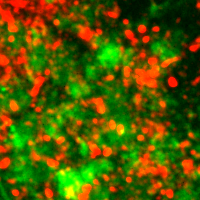

Supplement: Supplementary file 6 — Source data Fig. 5 [file 44319_2025_581_MOESM6_ESM.zip › 5 G/Cdc42 CA Bottom.tif]

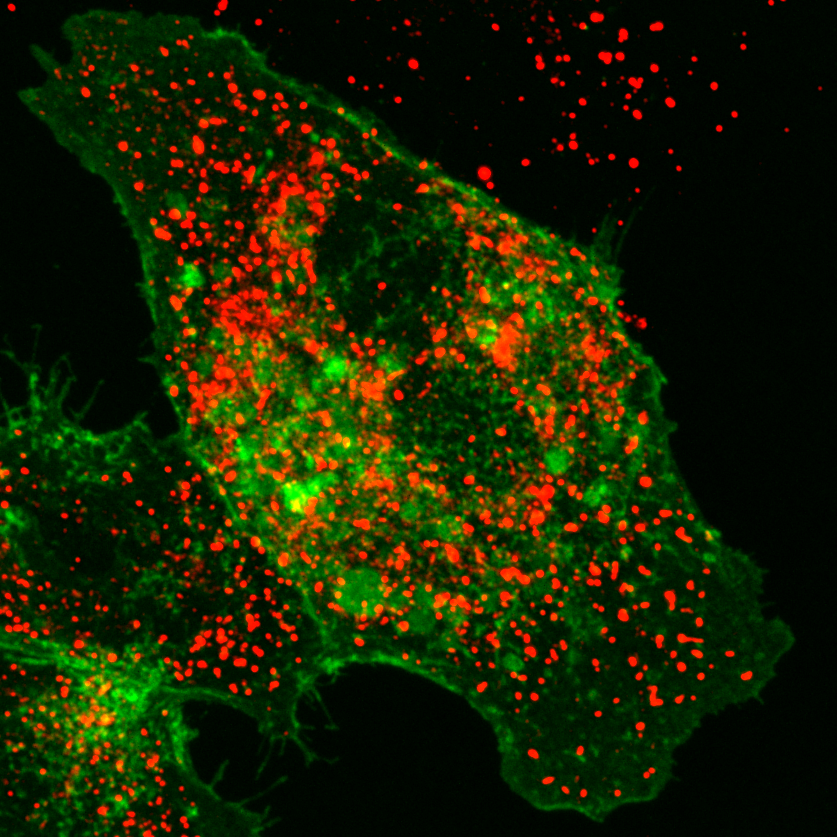

Supplement: Supplementary file 6 — Source data Fig. 5 [file 44319_2025_581_MOESM6_ESM.zip › 5 G/Cdc42 CA Top.tif]

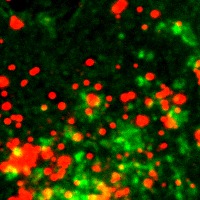

Supplement: Supplementary file 6 — Source data Fig. 5 [file 44319_2025_581_MOESM6_ESM.zip › 5 G/Cdc42 DN Bottom.tif]

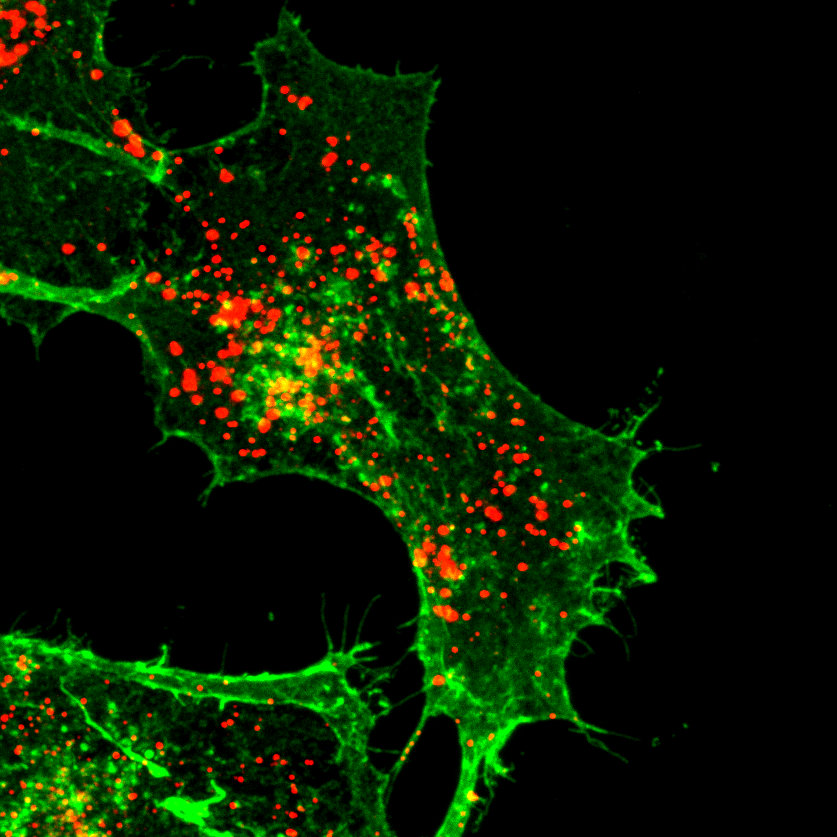

Supplement: Supplementary file 6 — Source data Fig. 5 [file 44319_2025_581_MOESM6_ESM.zip › 5 G/Cdc42 DN Top.tif]

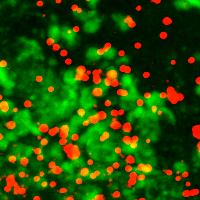

Supplement: Supplementary file 6 — Source data Fig. 5 [file 44319_2025_581_MOESM6_ESM.zip › 5 G/Cdc42 WT Bottom.tif]

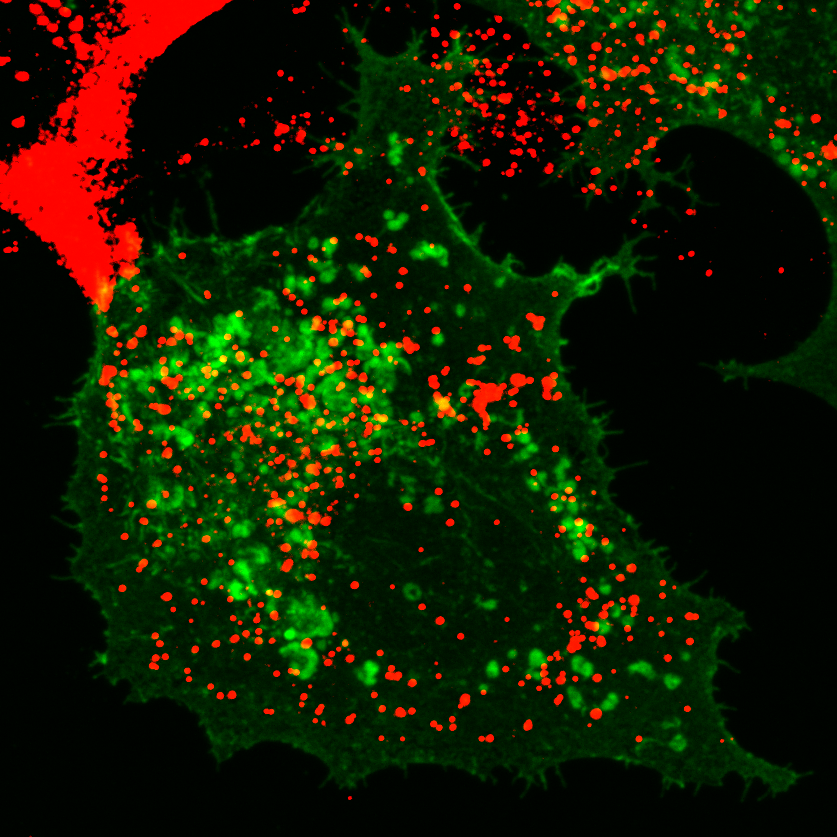

Supplement: Supplementary file 6 — Source data Fig. 5 [file 44319_2025_581_MOESM6_ESM.zip › 5 G/Cdc42 WT Top.tif]

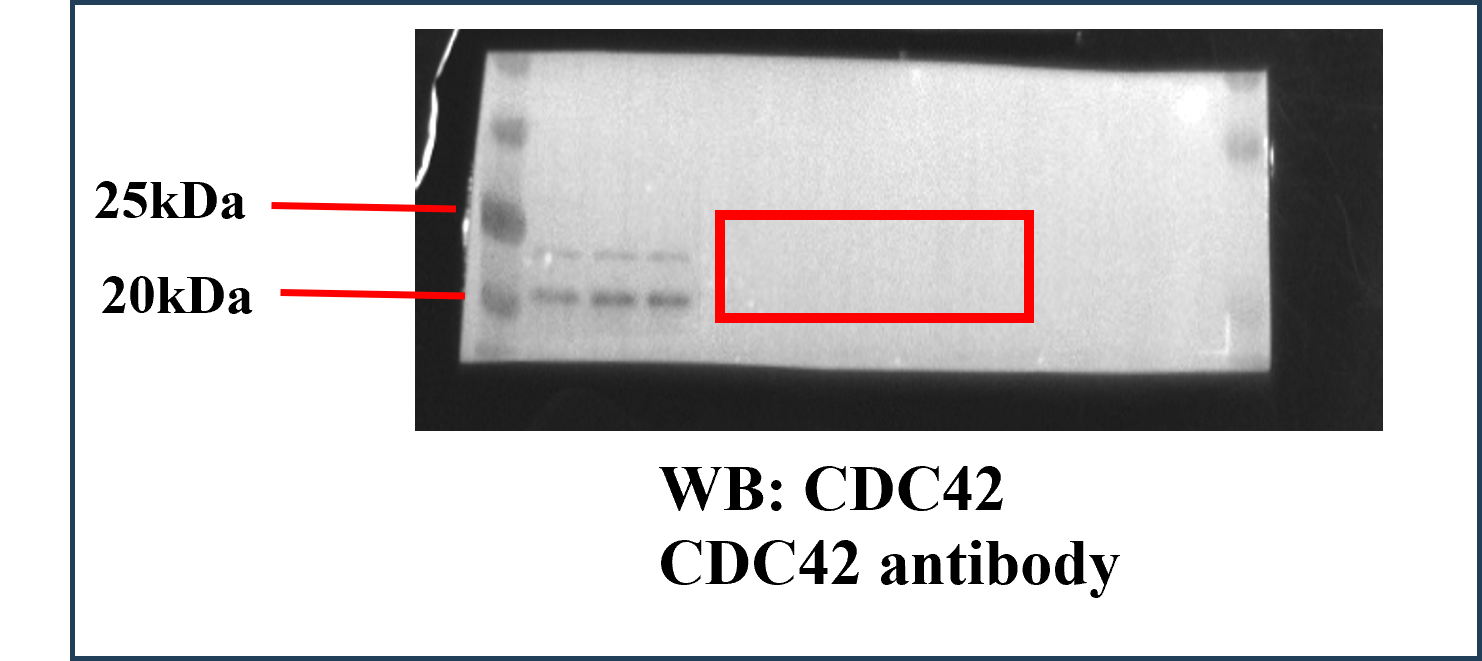

Supplement: Supplementary file 6 — Source data Fig. 5 [file 44319_2025_581_MOESM6_ESM.zip › 5 H/CDC42 IgG IP.tif]

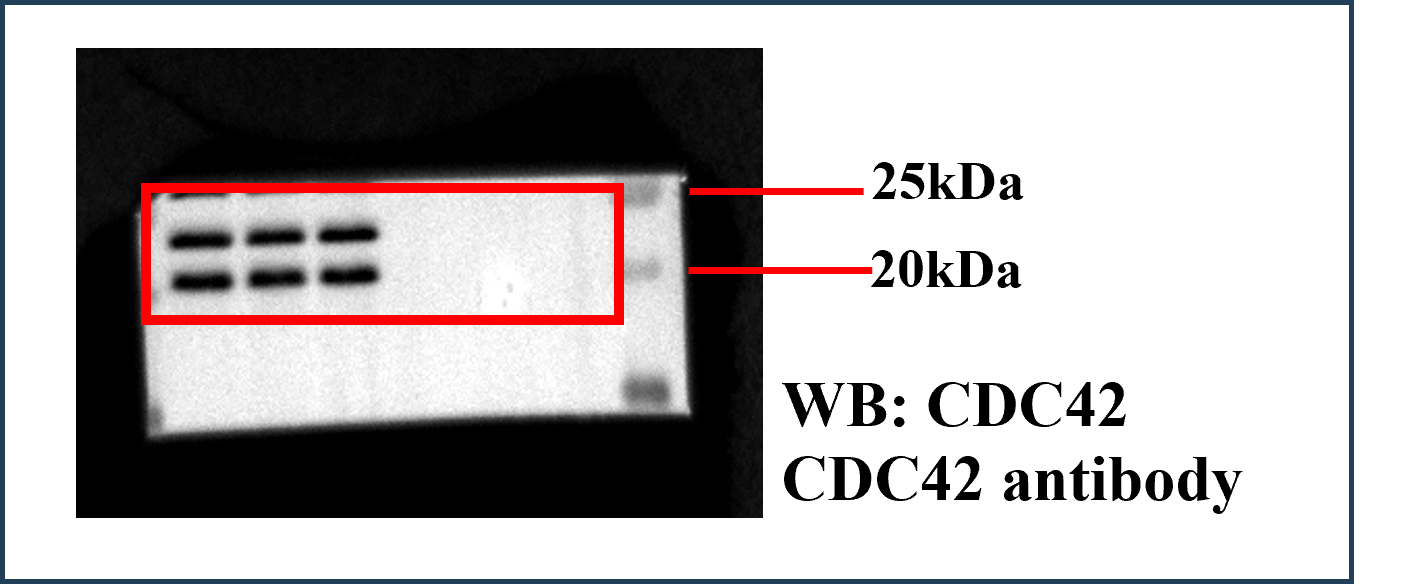

Supplement: Supplementary file 6 — Source data Fig. 5 [file 44319_2025_581_MOESM6_ESM.zip › 5 H/CDC42 input and Rab11 IP.tif]

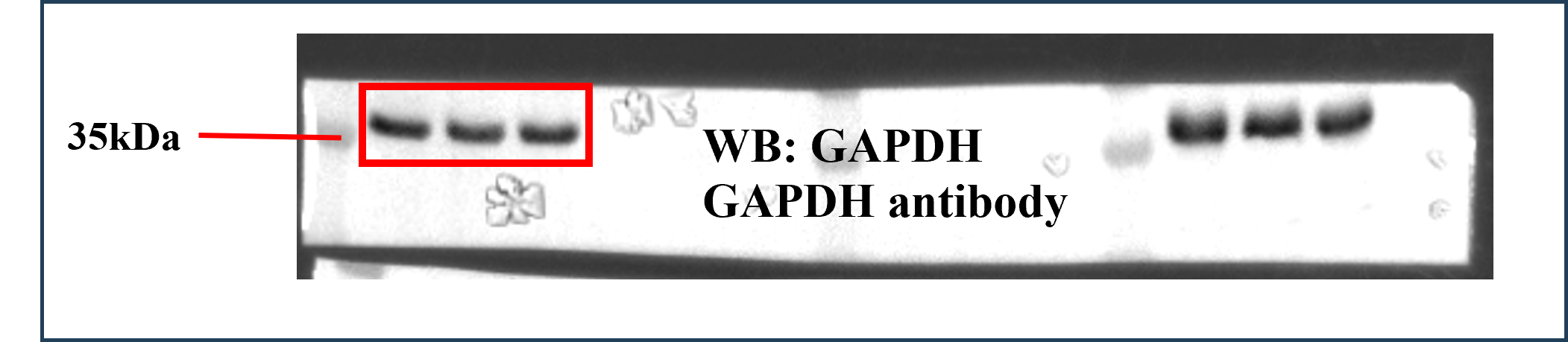

Supplement: Supplementary file 6 — Source data Fig. 5 [file 44319_2025_581_MOESM6_ESM.zip › 5 H/GAPDH.tif]

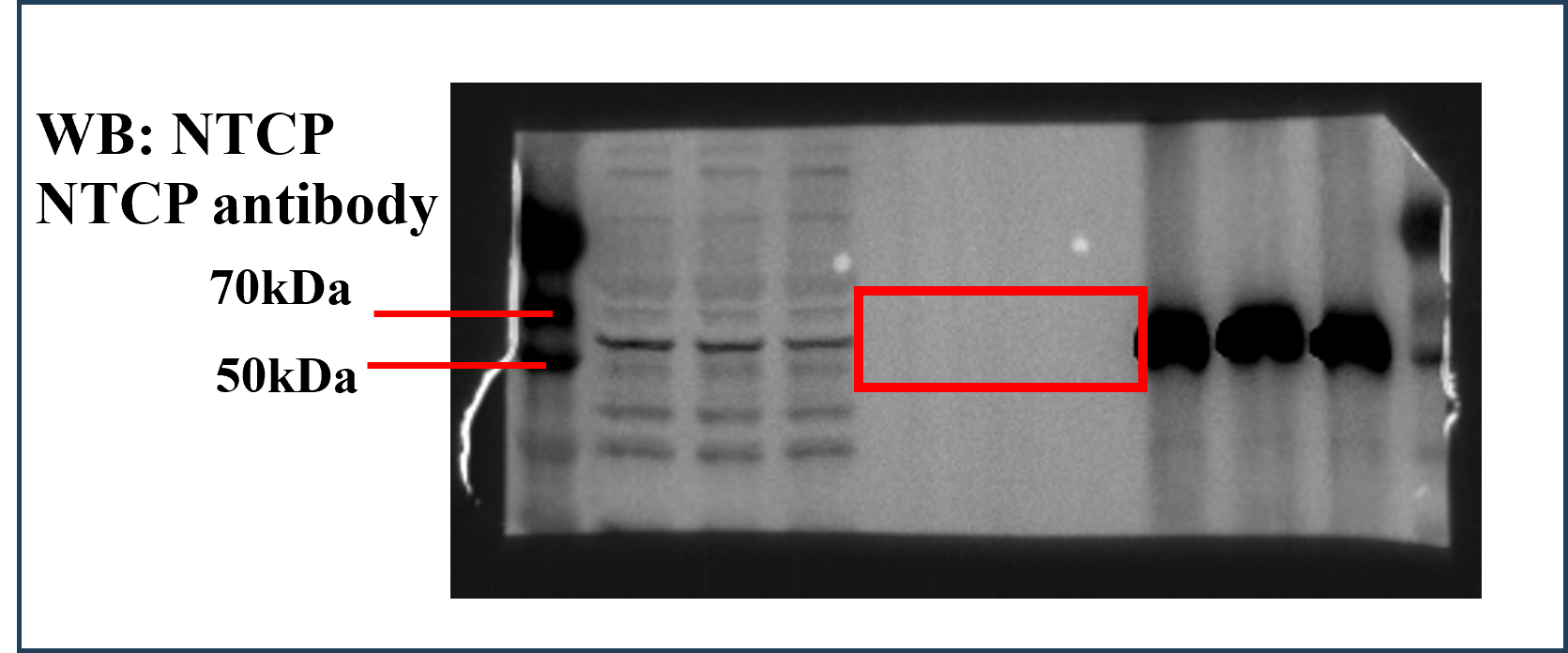

Supplement: Supplementary file 6 — Source data Fig. 5 [file 44319_2025_581_MOESM6_ESM.zip › 5 H/NTCP IgG IP.tif]

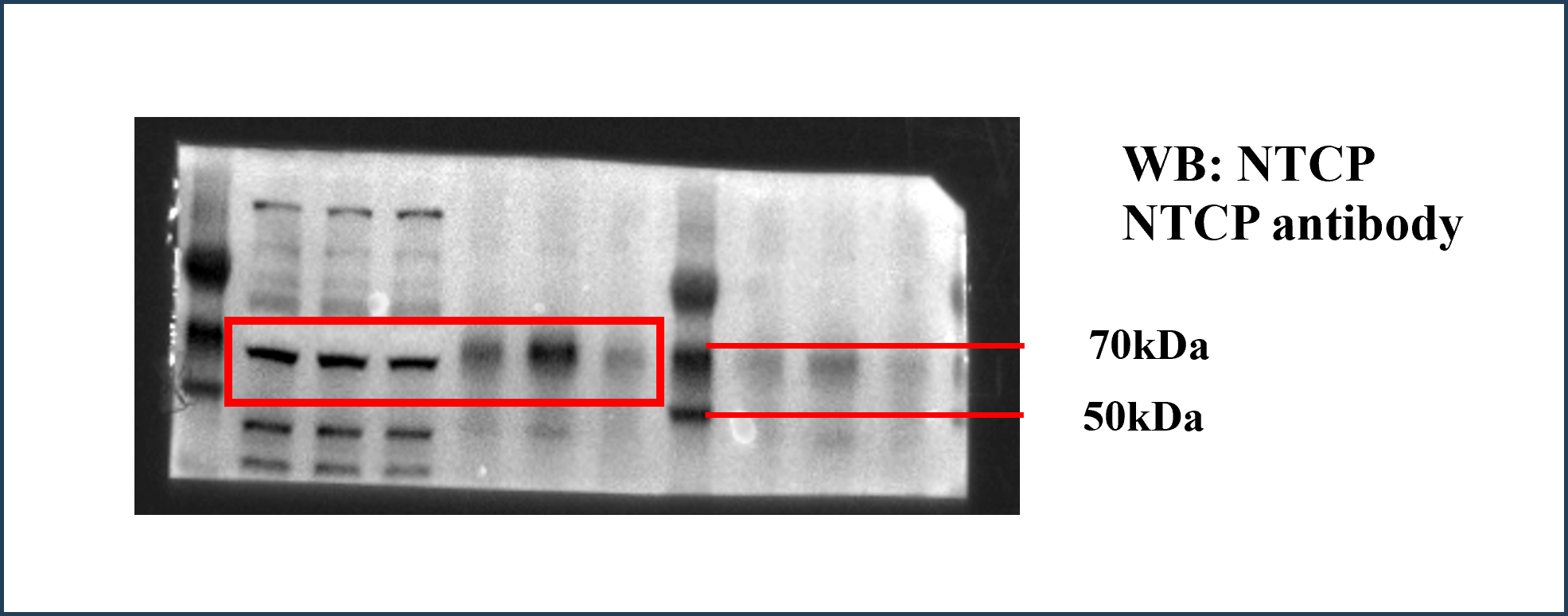

Supplement: Supplementary file 6 — Source data Fig. 5 [file 44319_2025_581_MOESM6_ESM.zip › 5 H/NTCP input and Rab11 IP.tif]

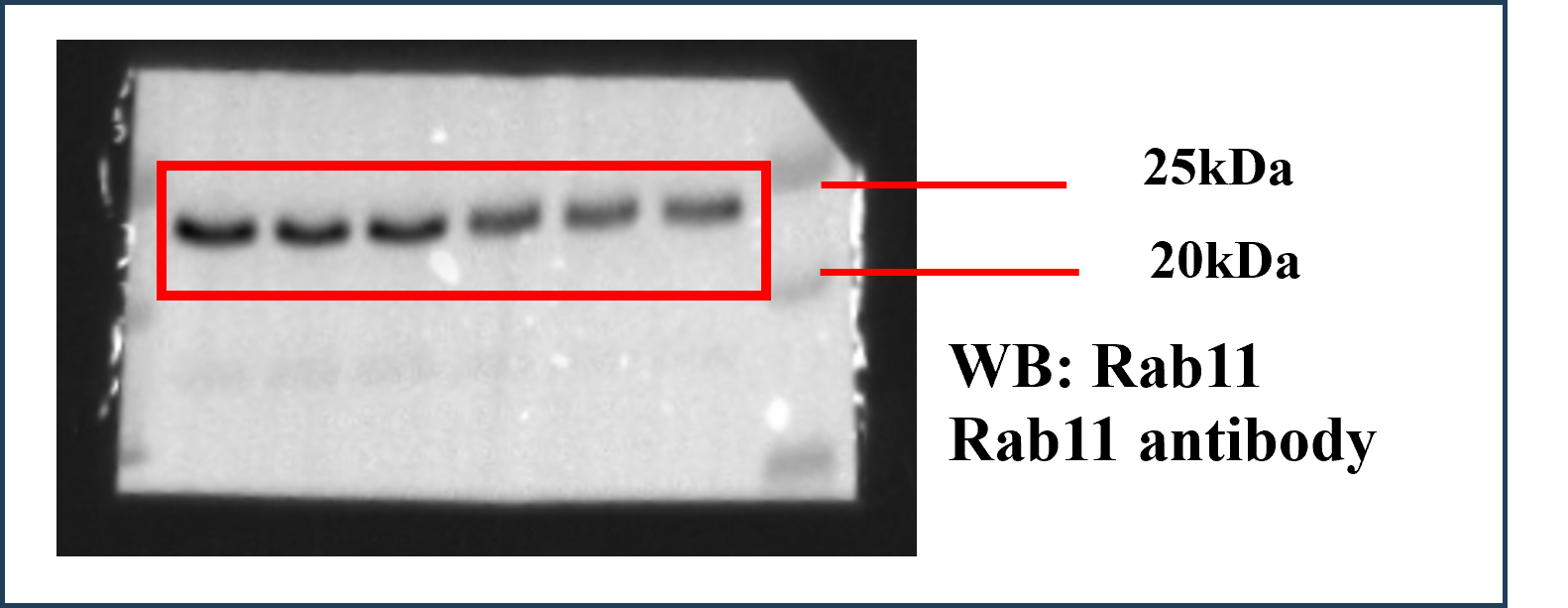

Supplement: Supplementary file 6 — Source data Fig. 5 [file 44319_2025_581_MOESM6_ESM.zip › 5 H/Rab11 Rab11 IP and input.tif]

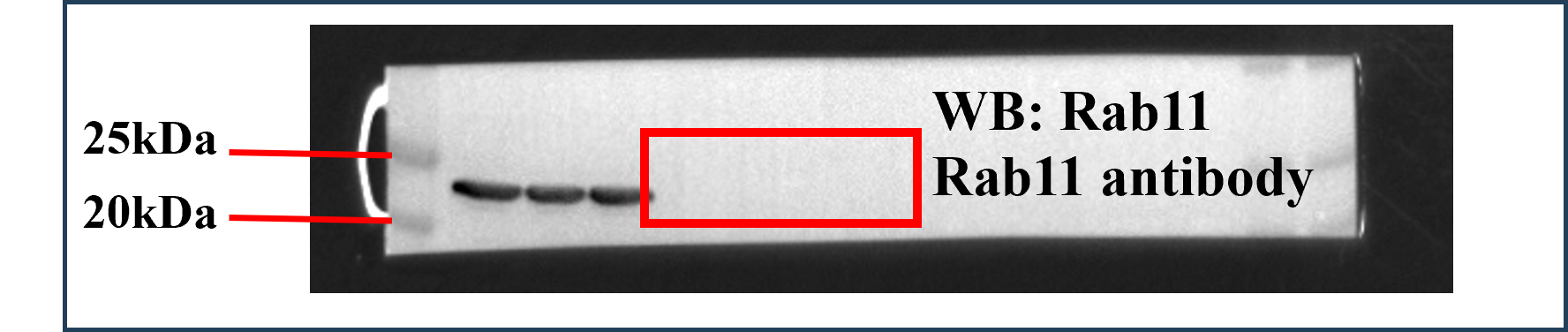

Supplement: Supplementary file 6 — Source data Fig. 5 [file 44319_2025_581_MOESM6_ESM.zip › 5 H/Rab11 IgG IP.tif]

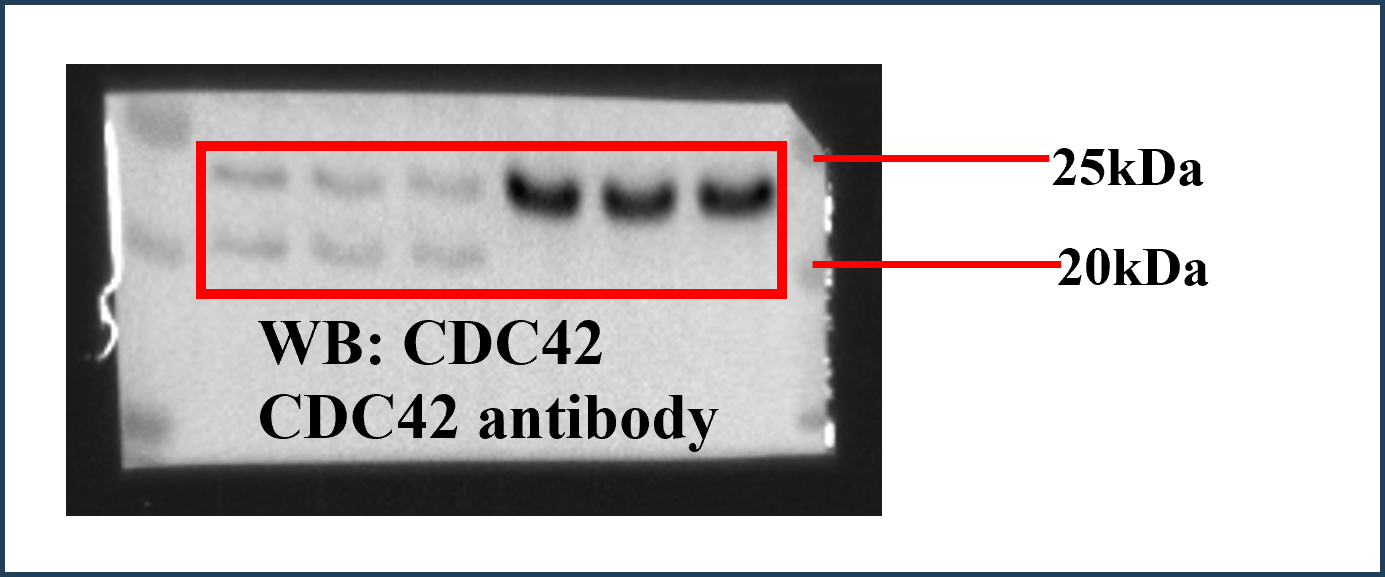

Supplement: Supplementary file 6 — Source data Fig. 5 [file 44319_2025_581_MOESM6_ESM.zip › 5 I/CDC42 input and IP.tif]

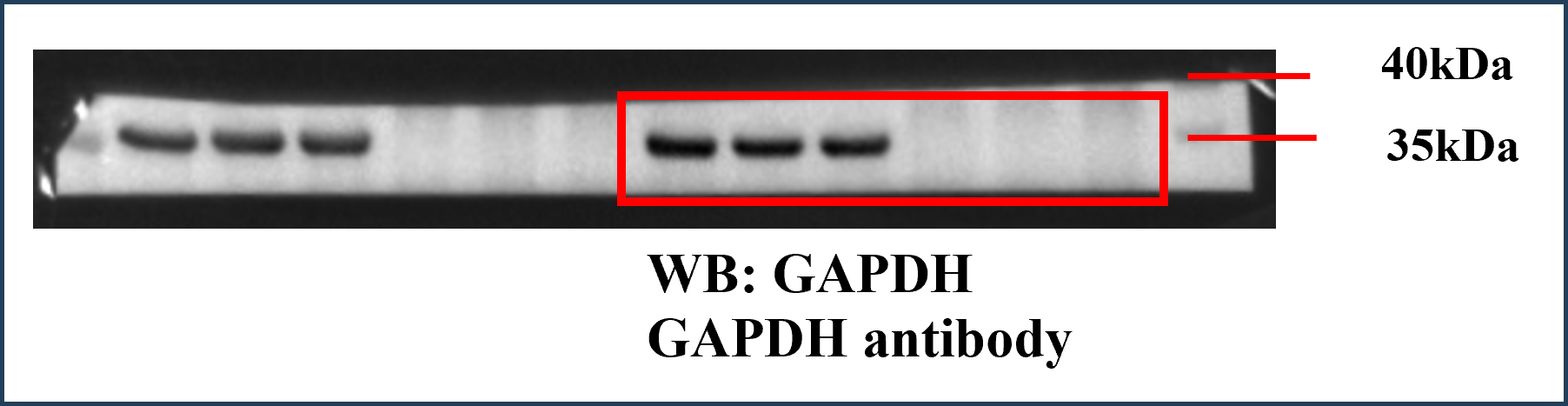

Supplement: Supplementary file 6 — Source data Fig. 5 [file 44319_2025_581_MOESM6_ESM.zip › 5 I/GAPDH.tif]

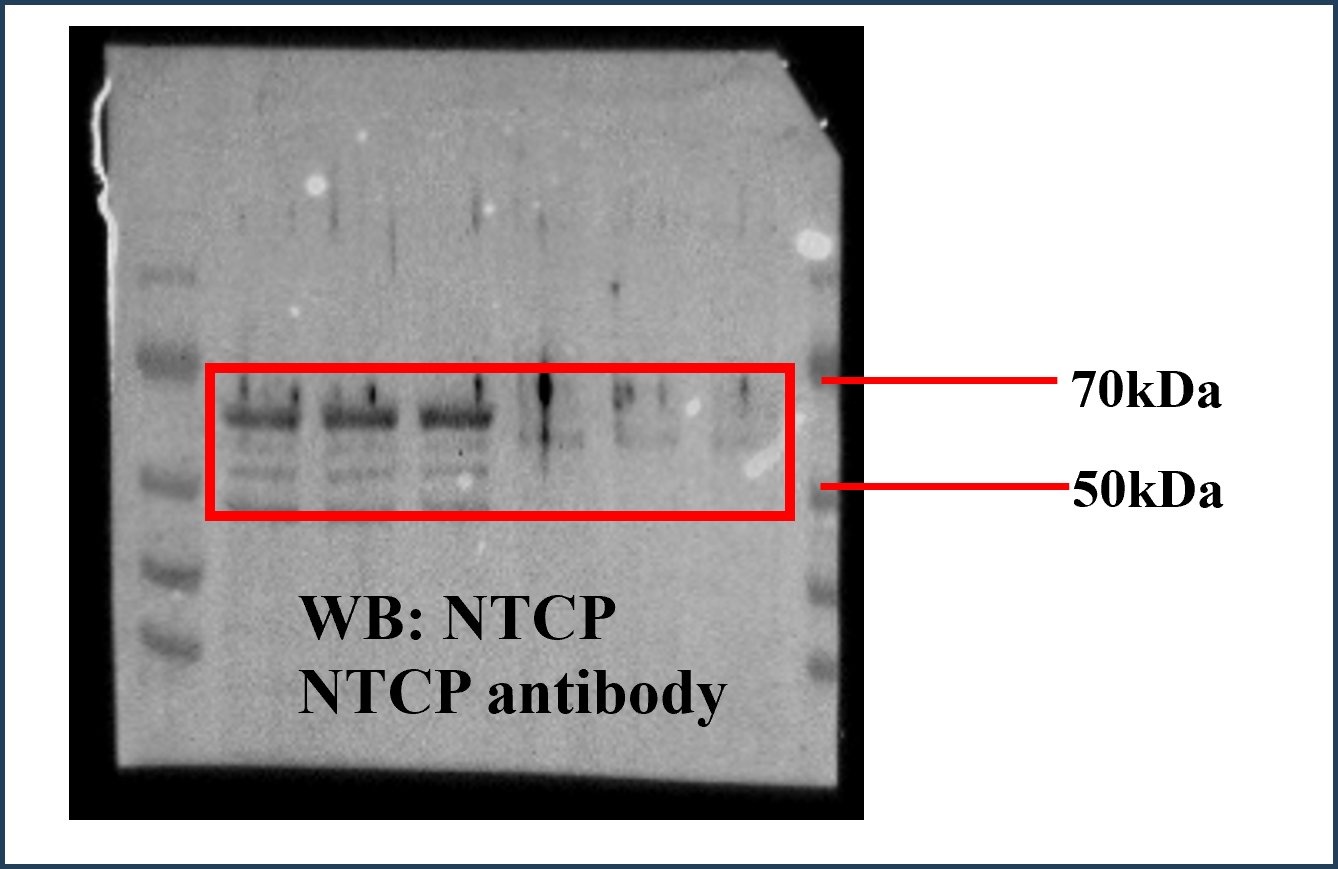

Supplement: Supplementary file 6 — Source data Fig. 5 [file 44319_2025_581_MOESM6_ESM.zip › 5 I/NTCP input and IP.tif]

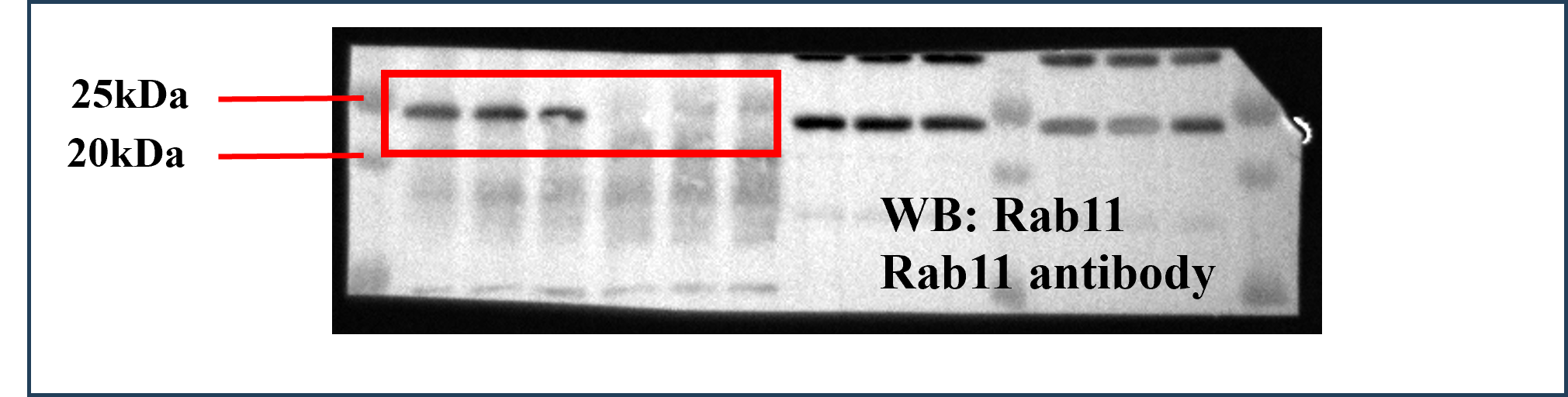

Supplement: Supplementary file 6 — Source data Fig. 5 [file 44319_2025_581_MOESM6_ESM.zip › 5 I/Rab11 input and IP.tif]

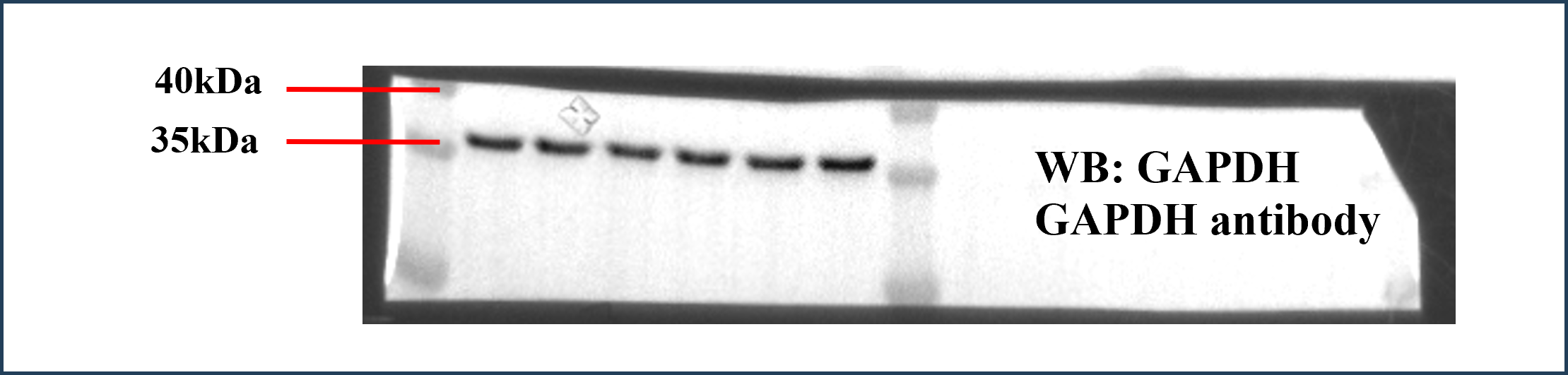

Supplement: Supplementary file 6 — Source data Fig. 5 [file 44319_2025_581_MOESM6_ESM.zip › 5 J/GAPDH.tif]

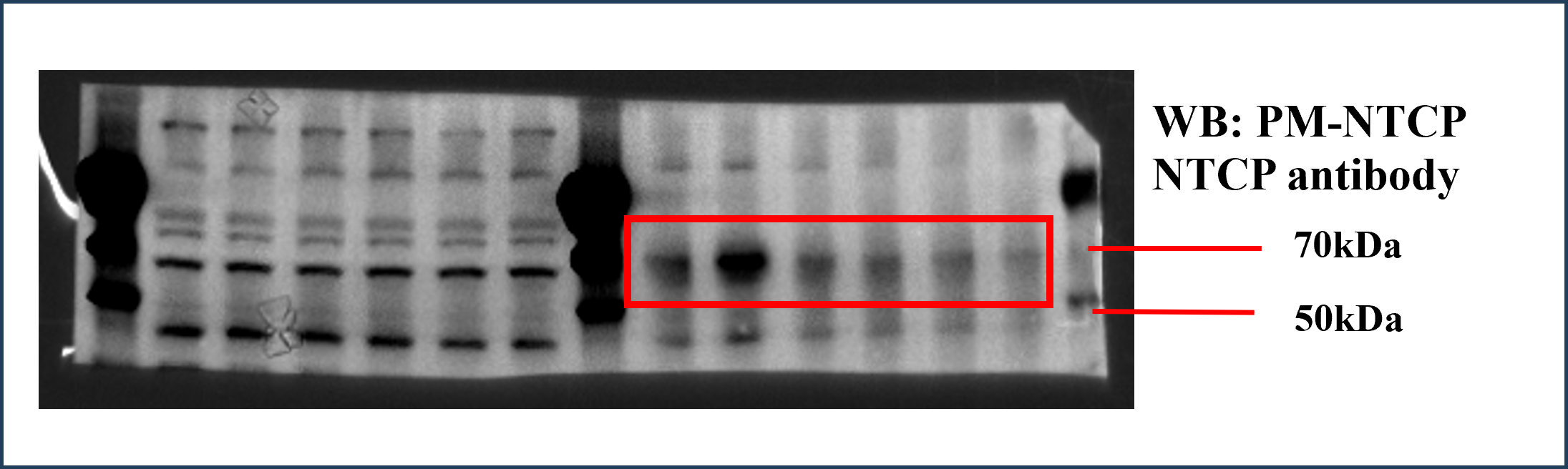

Supplement: Supplementary file 6 — Source data Fig. 5 [file 44319_2025_581_MOESM6_ESM.zip › 5 J/PM-NTCP.png]

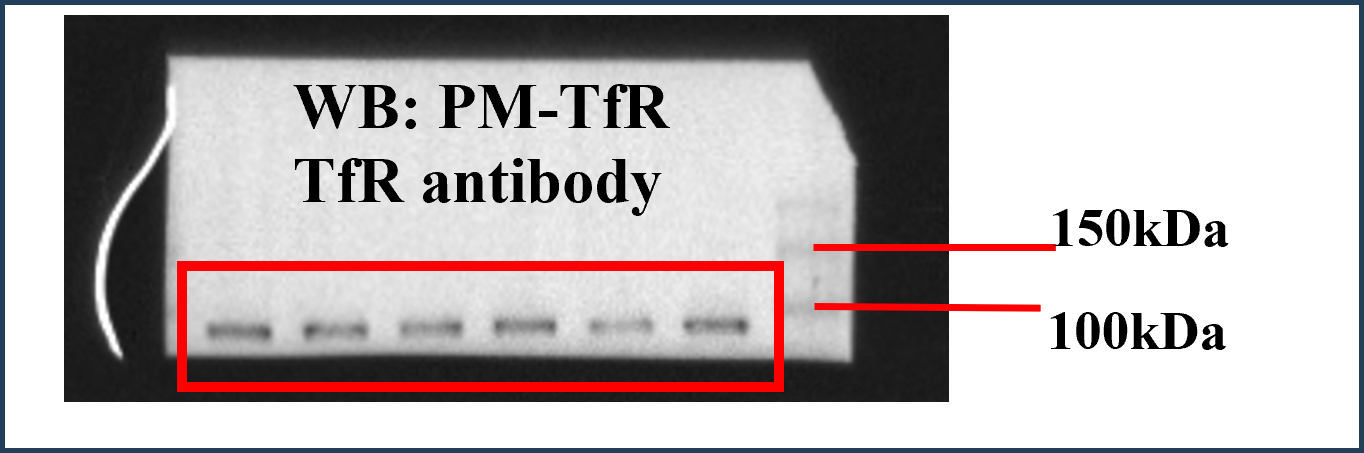

Supplement: Supplementary file 6 — Source data Fig. 5 [file 44319_2025_581_MOESM6_ESM.zip › 5 J/PM-TfR.tif]

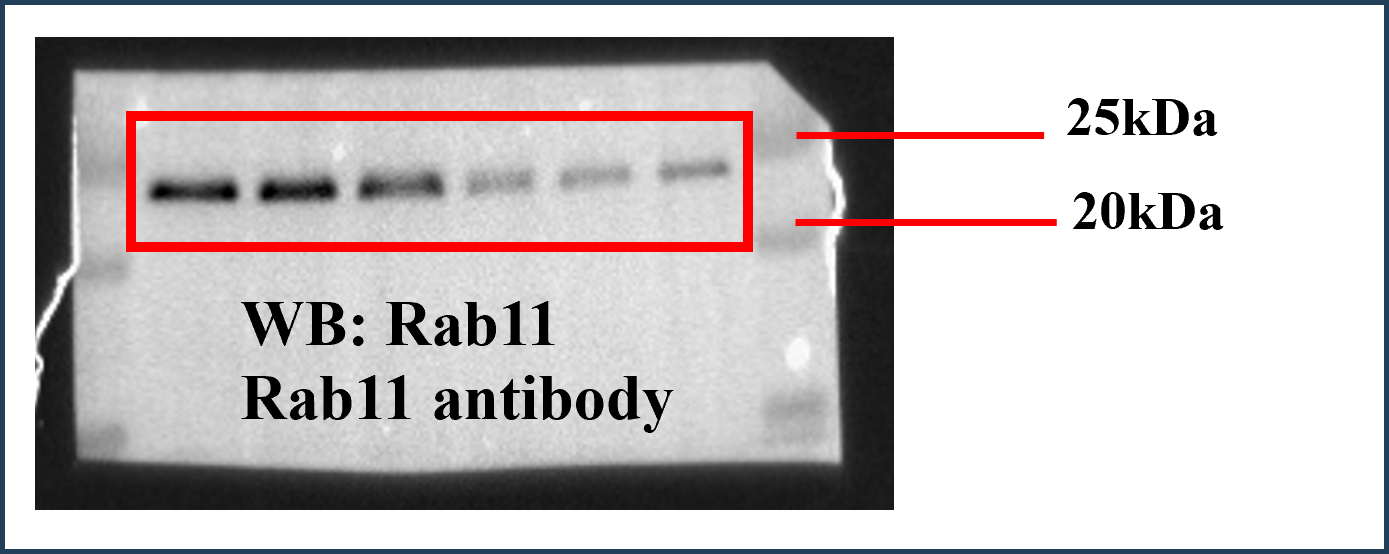

Supplement: Supplementary file 6 — Source data Fig. 5 [file 44319_2025_581_MOESM6_ESM.zip › 5 J/Rab11.tif]

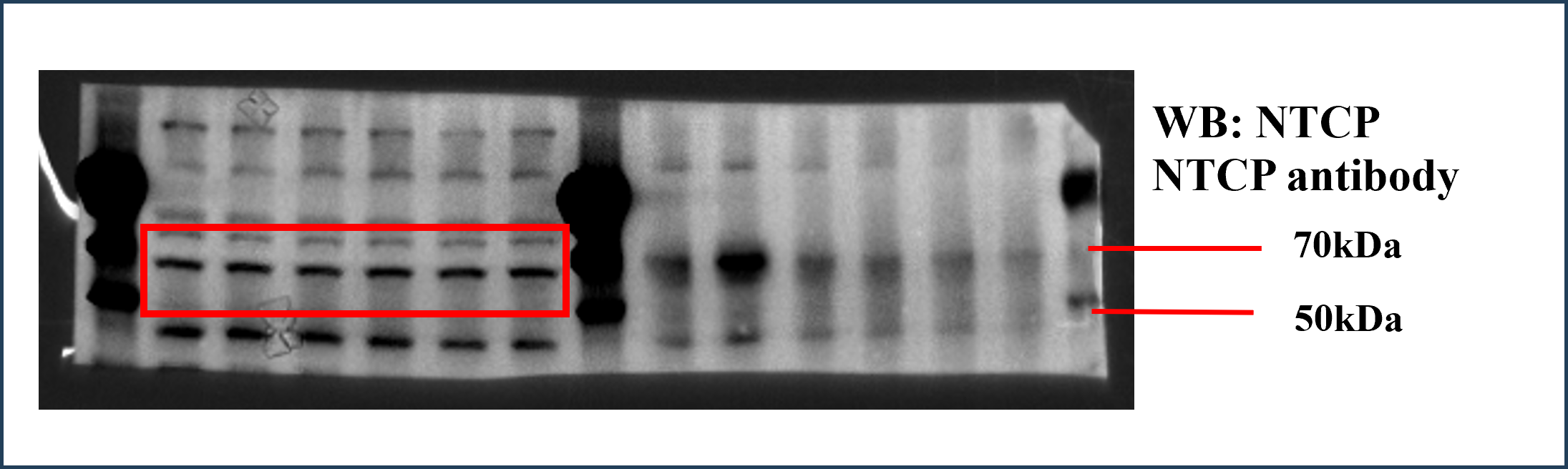

Supplement: Supplementary file 6 — Source data Fig. 5 [file 44319_2025_581_MOESM6_ESM.zip › 5 J/total NTCP.tif]

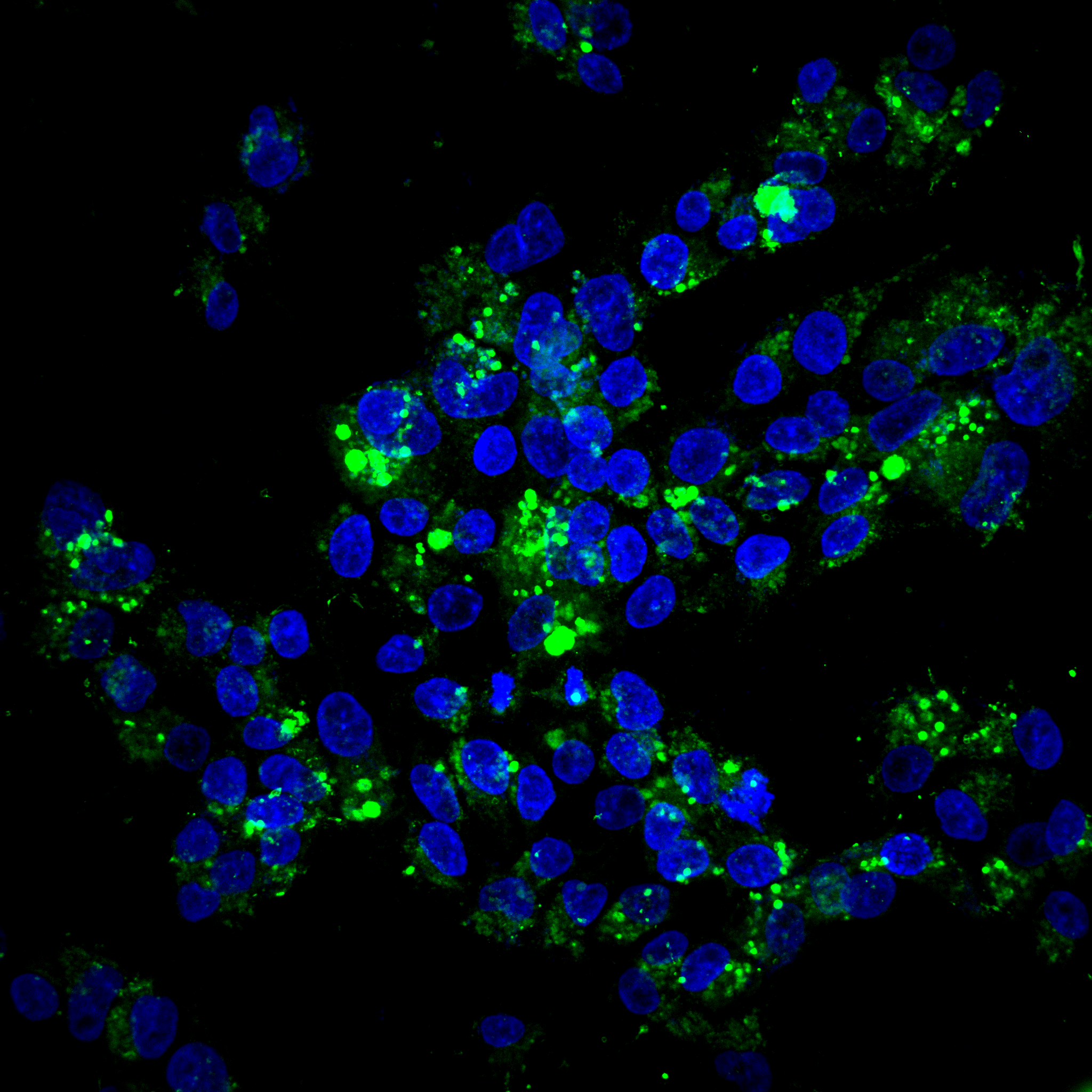

Supplement: Supplementary file 7 — Source data Fig. 6 [file 44319_2025_581_MOESM7_ESM.zip › 6 A/Cdc42 CA.tif]

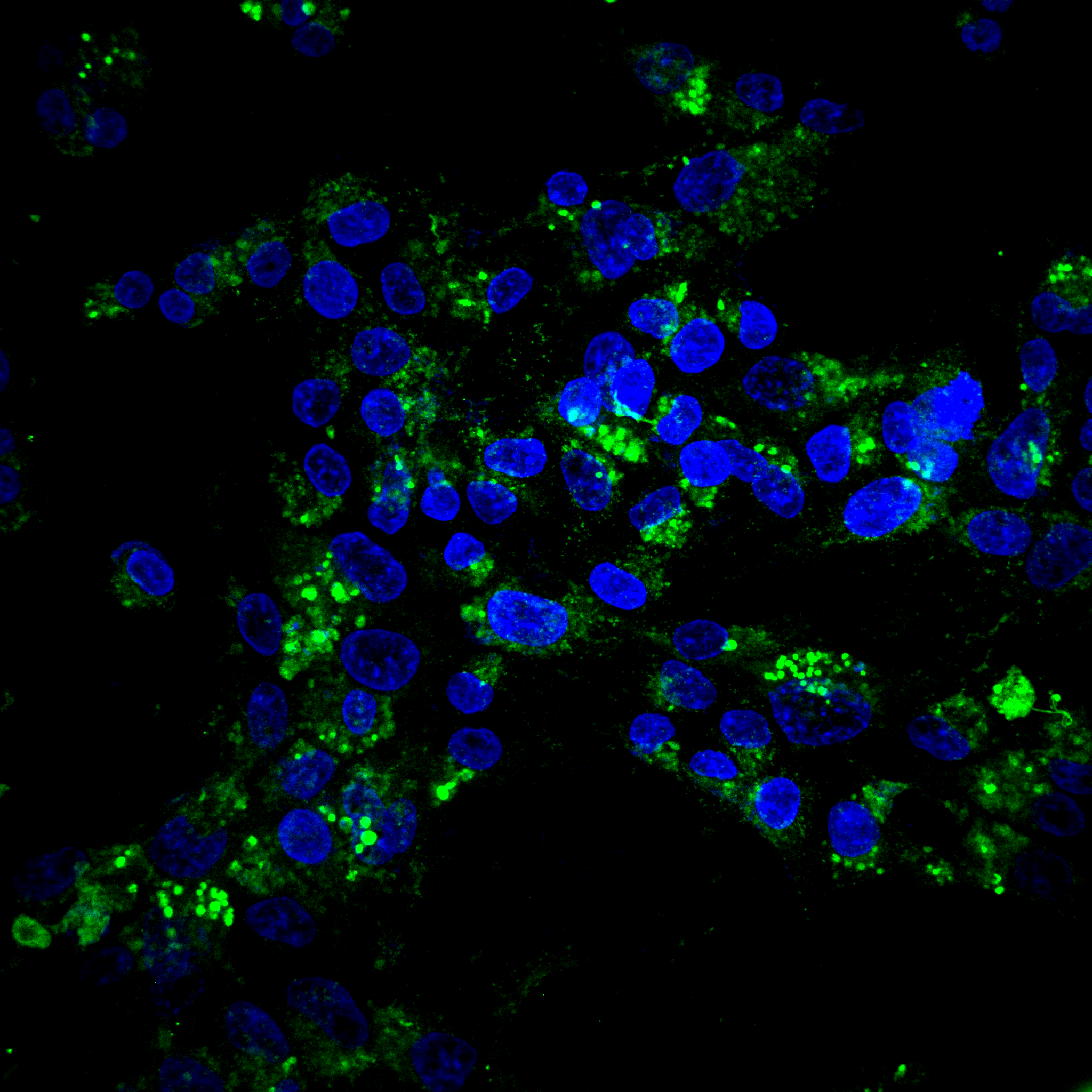

Supplement: Supplementary file 7 — Source data Fig. 6 [file 44319_2025_581_MOESM7_ESM.zip › 6 A/Cdc42 DN.tif]

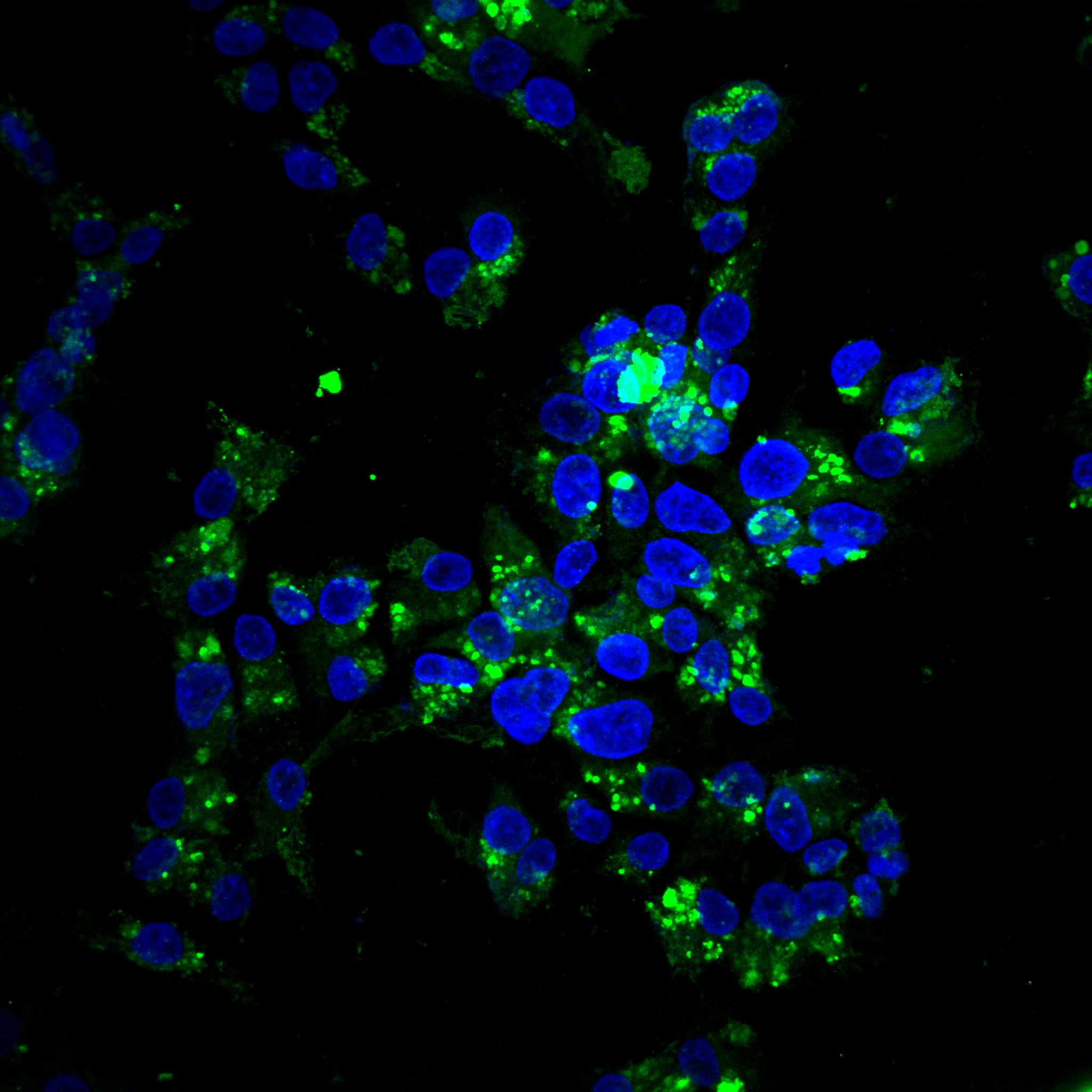

Supplement: Supplementary file 7 — Source data Fig. 6 [file 44319_2025_581_MOESM7_ESM.zip › 6 A/Cdc42 WT.tif]

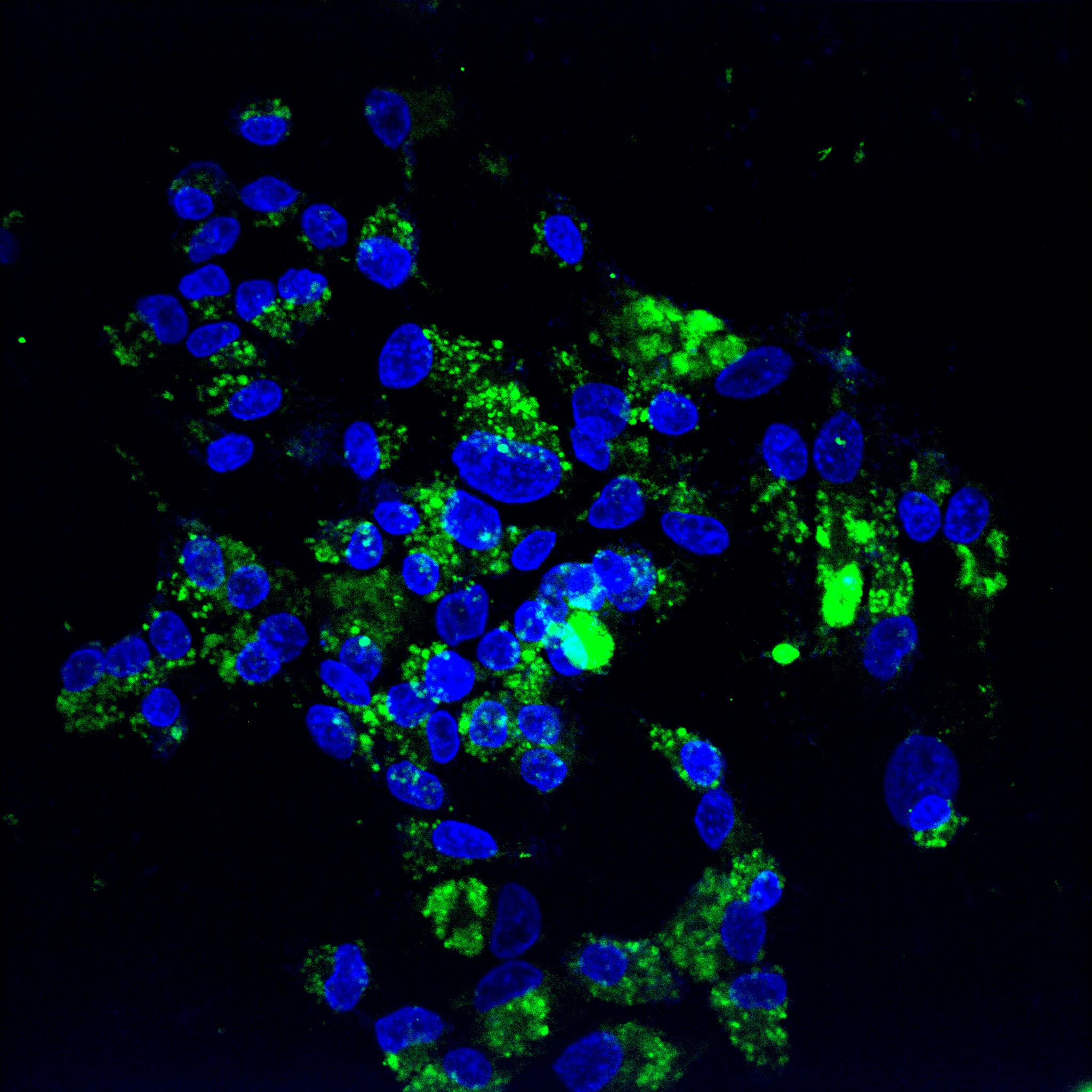

Supplement: Supplementary file 7 — Source data Fig. 6 [file 44319_2025_581_MOESM7_ESM.zip › 6 C/Bradykinin.tif]

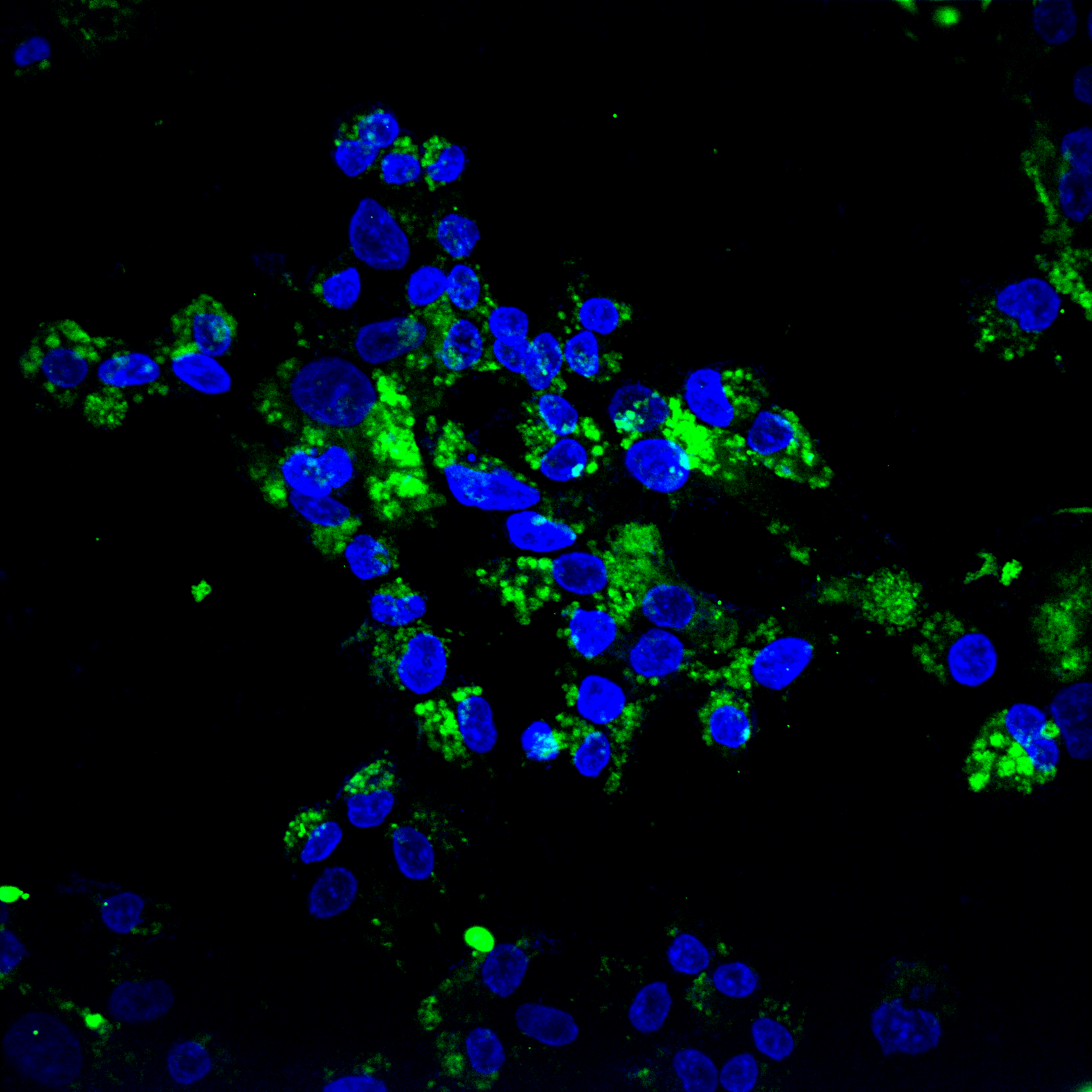

Supplement: Supplementary file 7 — Source data Fig. 6 [file 44319_2025_581_MOESM7_ESM.zip › 6 C/ML141.tif]

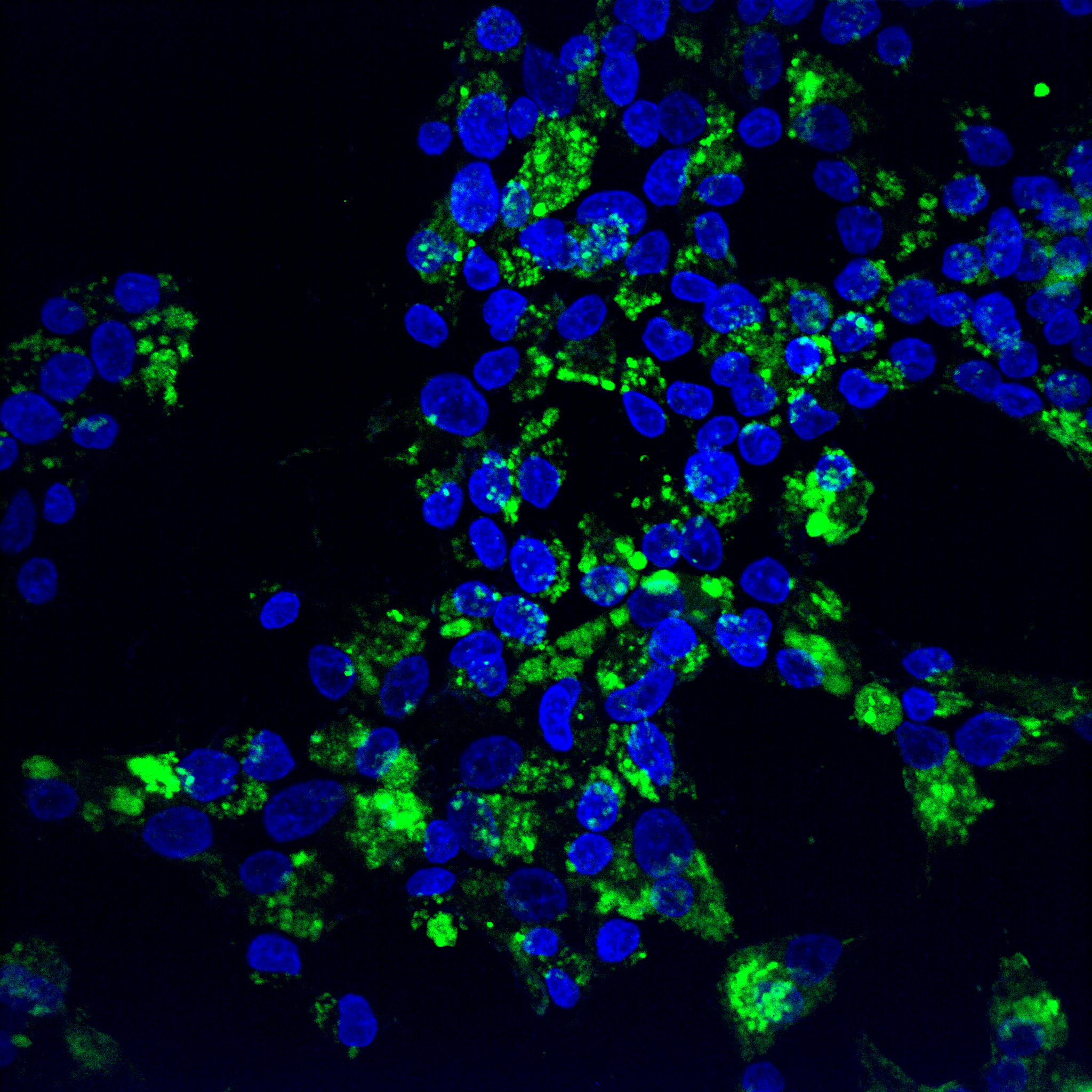

Supplement: Supplementary file 7 — Source data Fig. 6 [file 44319_2025_581_MOESM7_ESM.zip › 6 C/Untreated.tif]

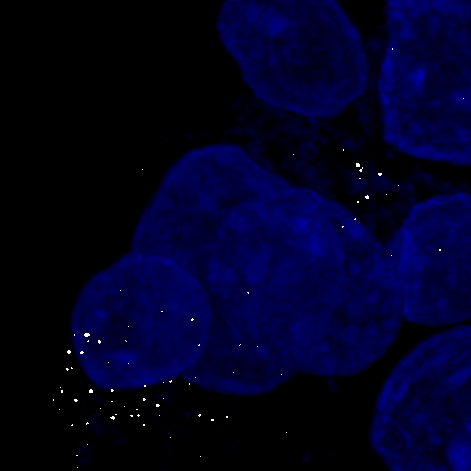

Supplement: Supplementary file 8 — Source data Fig. 7 [file 44319_2025_581_MOESM8_ESM.zip › 7 B/EIPA Bottom.tif]

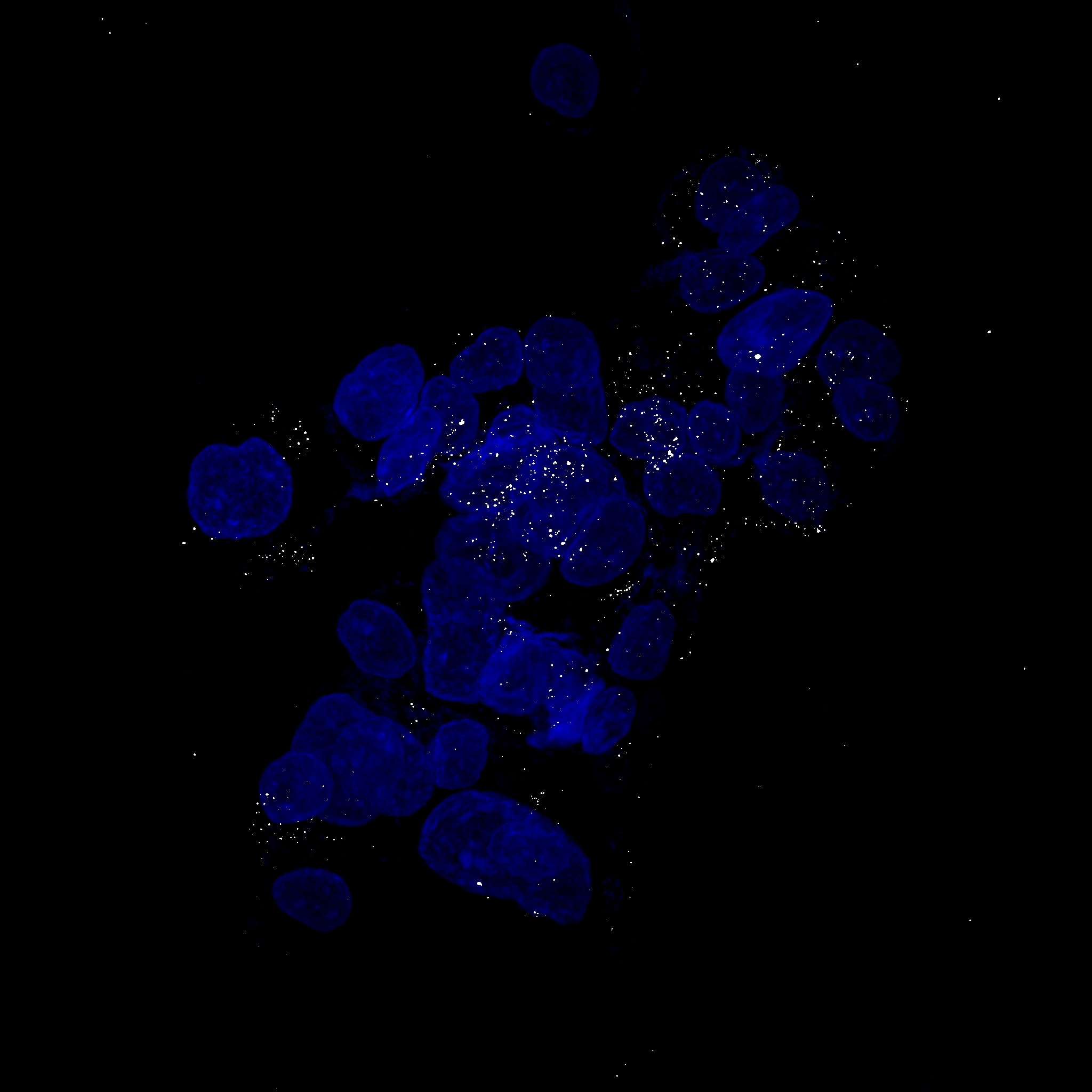

Supplement: Supplementary file 8 — Source data Fig. 7 [file 44319_2025_581_MOESM8_ESM.zip › 7 B/EIPA Top.tif]

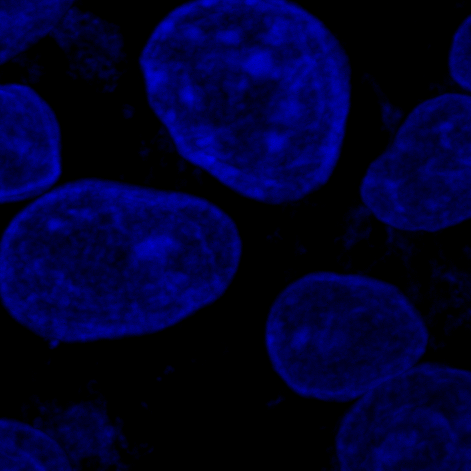

Supplement: Supplementary file 8 — Source data Fig. 7 [file 44319_2025_581_MOESM8_ESM.zip › 7 B/Noninfected Bottom.tif]

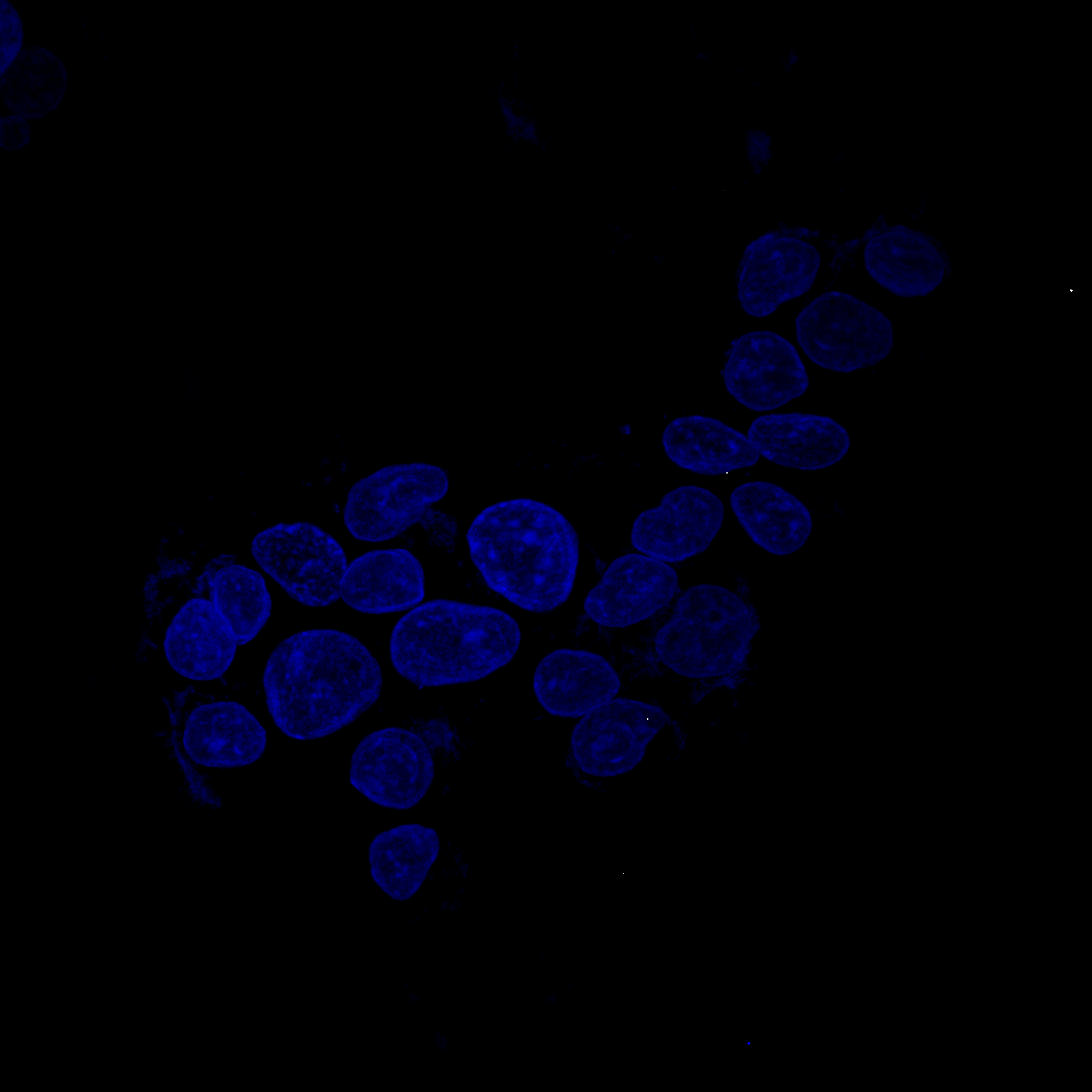

Supplement: Supplementary file 8 — Source data Fig. 7 [file 44319_2025_581_MOESM8_ESM.zip › 7 B/Noninfected Top.tif]

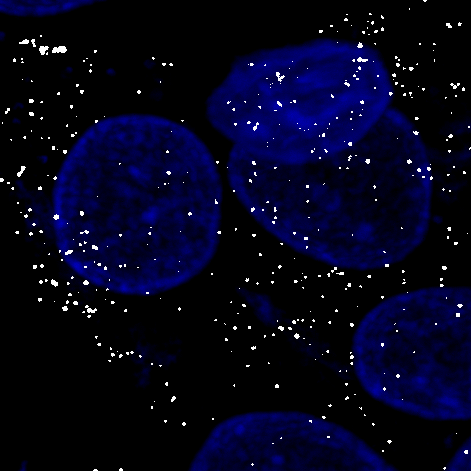

Supplement: Supplementary file 8 — Source data Fig. 7 [file 44319_2025_581_MOESM8_ESM.zip › 7 B/Untreated Bottom.tif]

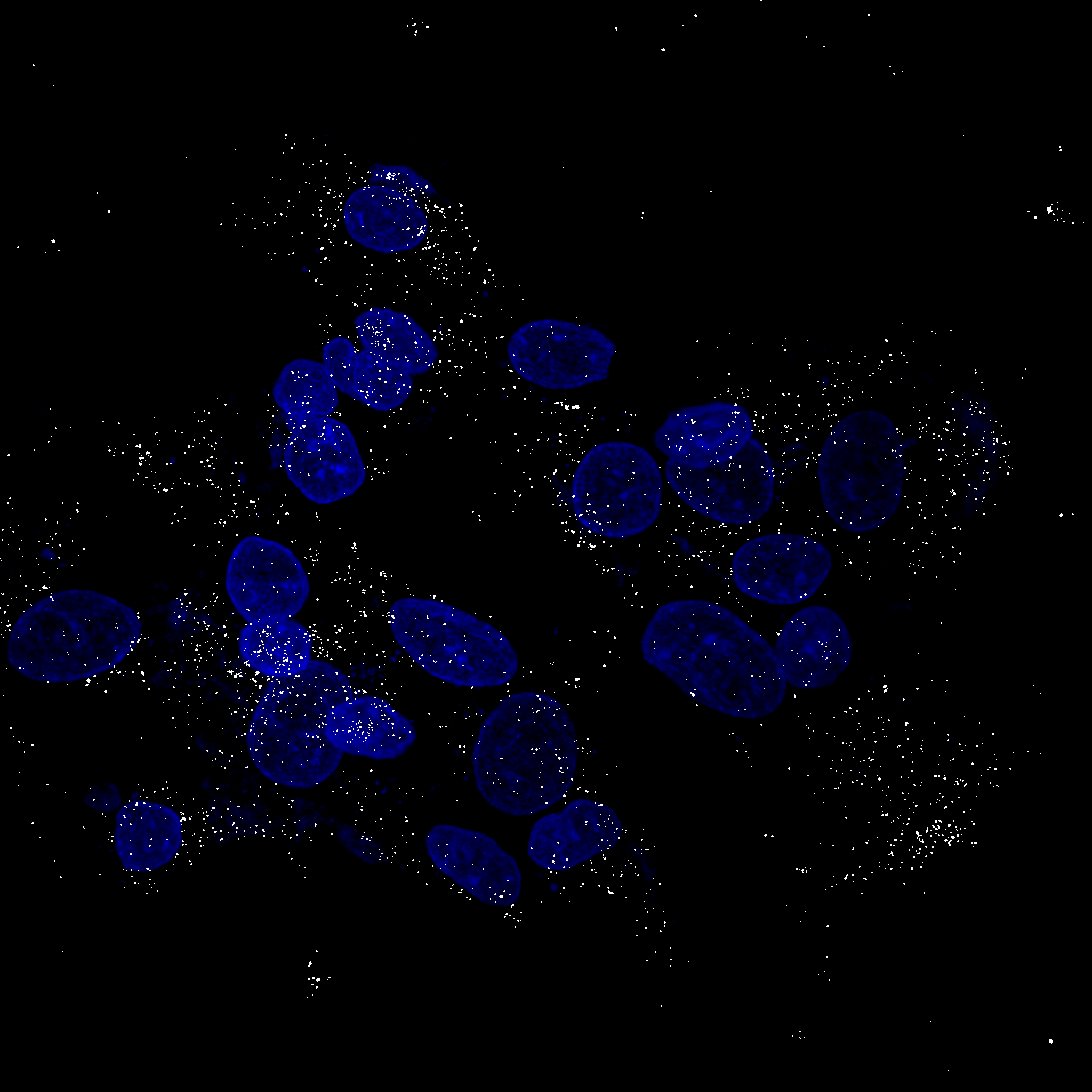

Supplement: Supplementary file 8 — Source data Fig. 7 [file 44319_2025_581_MOESM8_ESM.zip › 7 B/Untreated Top.tif]

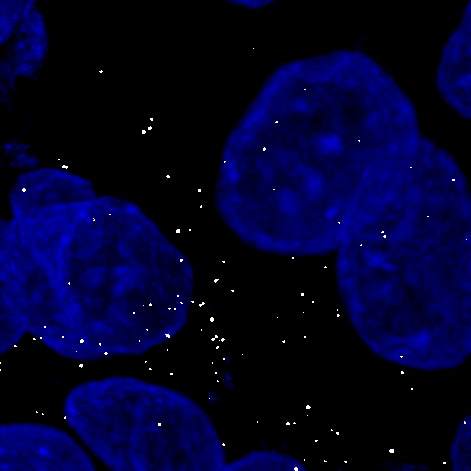

Supplement: Supplementary file 8 — Source data Fig. 7 [file 44319_2025_581_MOESM8_ESM.zip › 7 B/Wortmannin Bottom.tif]

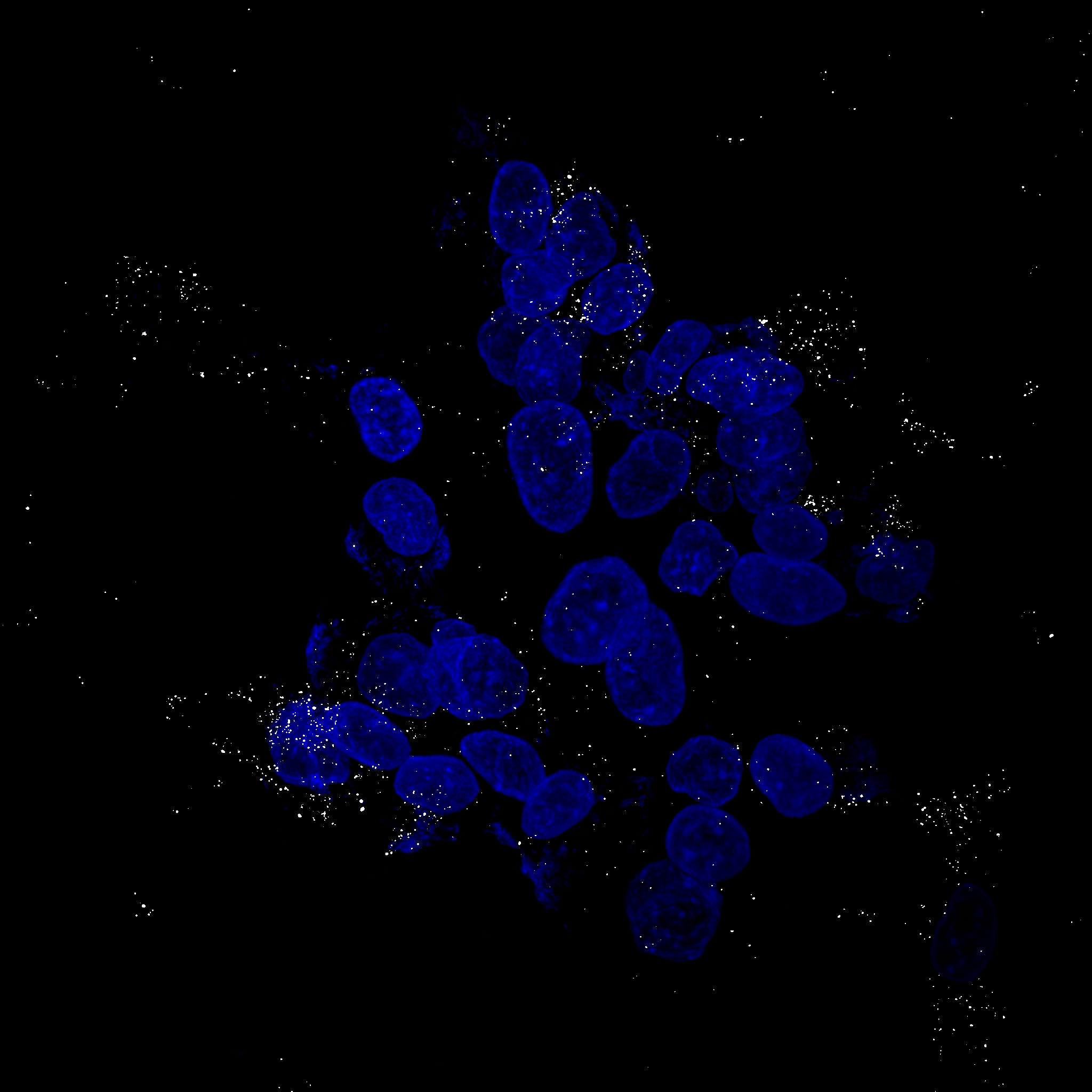

Supplement: Supplementary file 8 — Source data Fig. 7 [file 44319_2025_581_MOESM8_ESM.zip › 7 B/Wortmannin Top.tif]

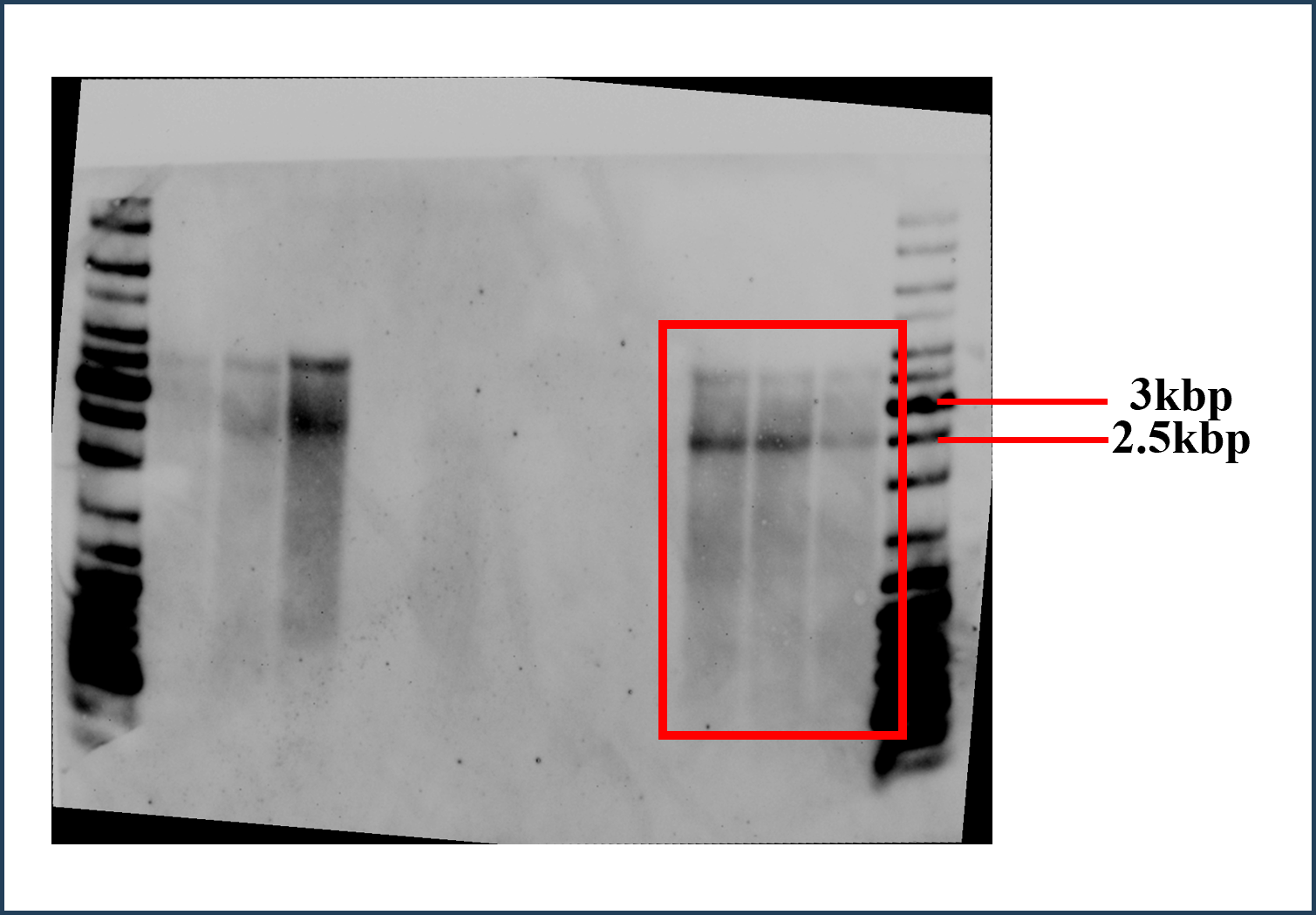

Supplement: Supplementary file 8 — Source data Fig. 7 [file 44319_2025_581_MOESM8_ESM.zip › 7 D/HBV DNA southern.tif]

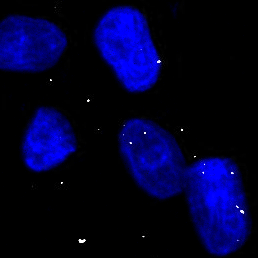

Supplement: Supplementary file 9 — Figure EV2 Source Data [file 44319_2025_581_MOESM9_ESM.zip › EV2/C/2hpi CA zoom.tif]

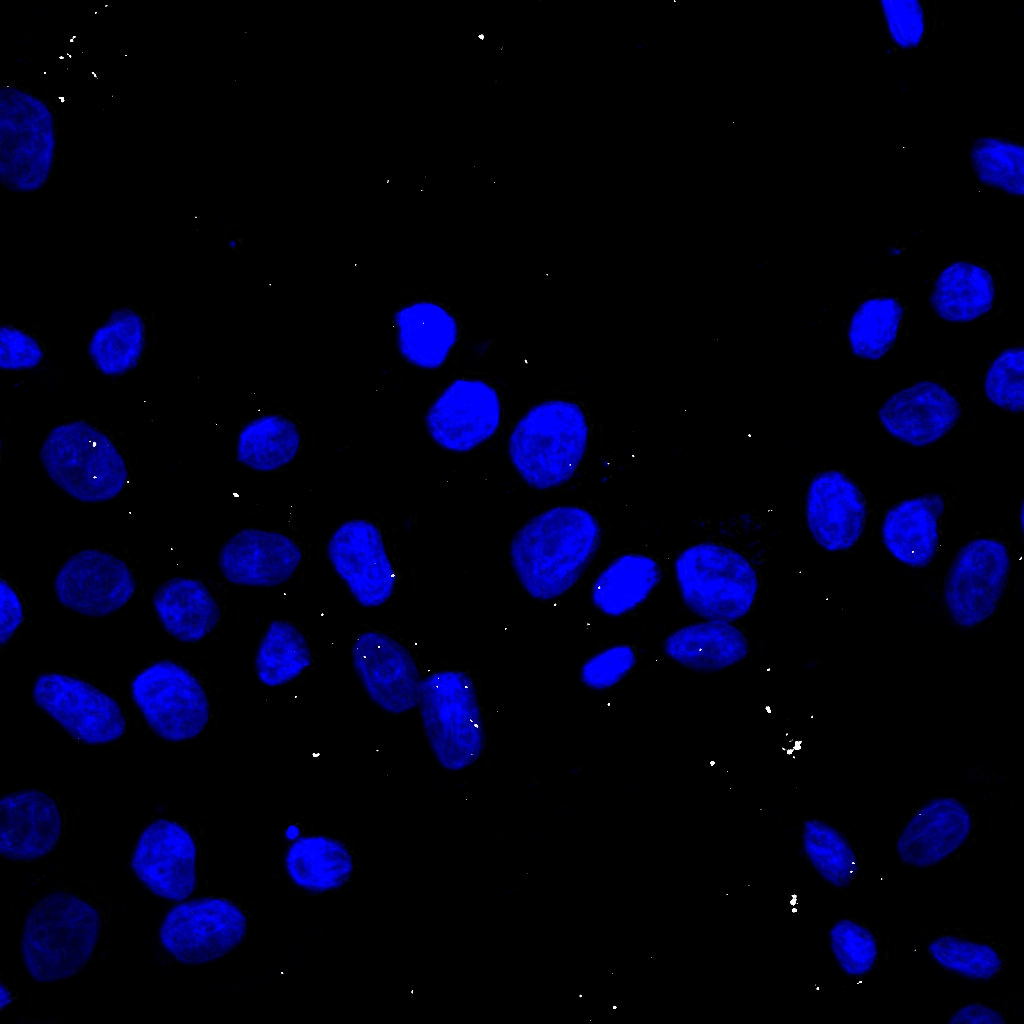

Supplement: Supplementary file 9 — Figure EV2 Source Data [file 44319_2025_581_MOESM9_ESM.zip › EV2/C/2hpi CA.tif]

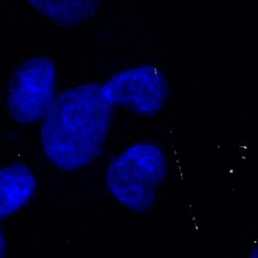

Supplement: Supplementary file 9 — Figure EV2 Source Data [file 44319_2025_581_MOESM9_ESM.zip › EV2/C/2hpi DN zoom.tif]

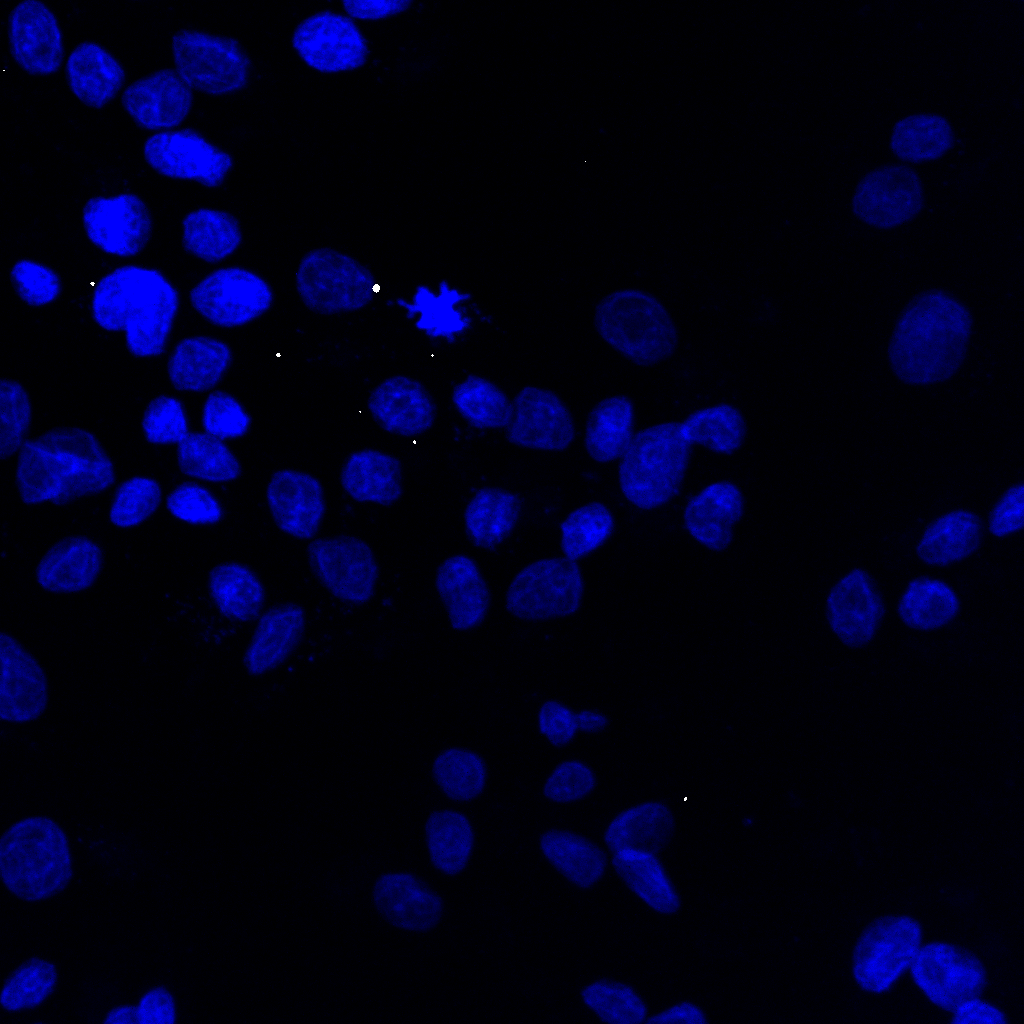

Supplement: Supplementary file 9 — Figure EV2 Source Data [file 44319_2025_581_MOESM9_ESM.zip › EV2/C/2hpi DN.tif]
