# Supplementary figures and images for: CDC42 supports HBV entry by NTCP translocation to the plasma membrane and macropinocytosis (part 2 of 2)
Source: EMBO Rep. 2025 Sep 15;26(21):5239–69. doi: 10.1038/s44319-025-00581-8 (PMC12592336; doi:10.1038/s44319-025-00581-8)

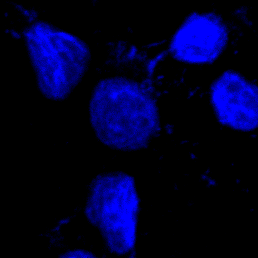

Supplement: Supplementary file 9 — Figure EV2 Source Data [file 44319_2025_581_MOESM9_ESM.zip › EV2/C/2hpi MOCK zoom.tif]

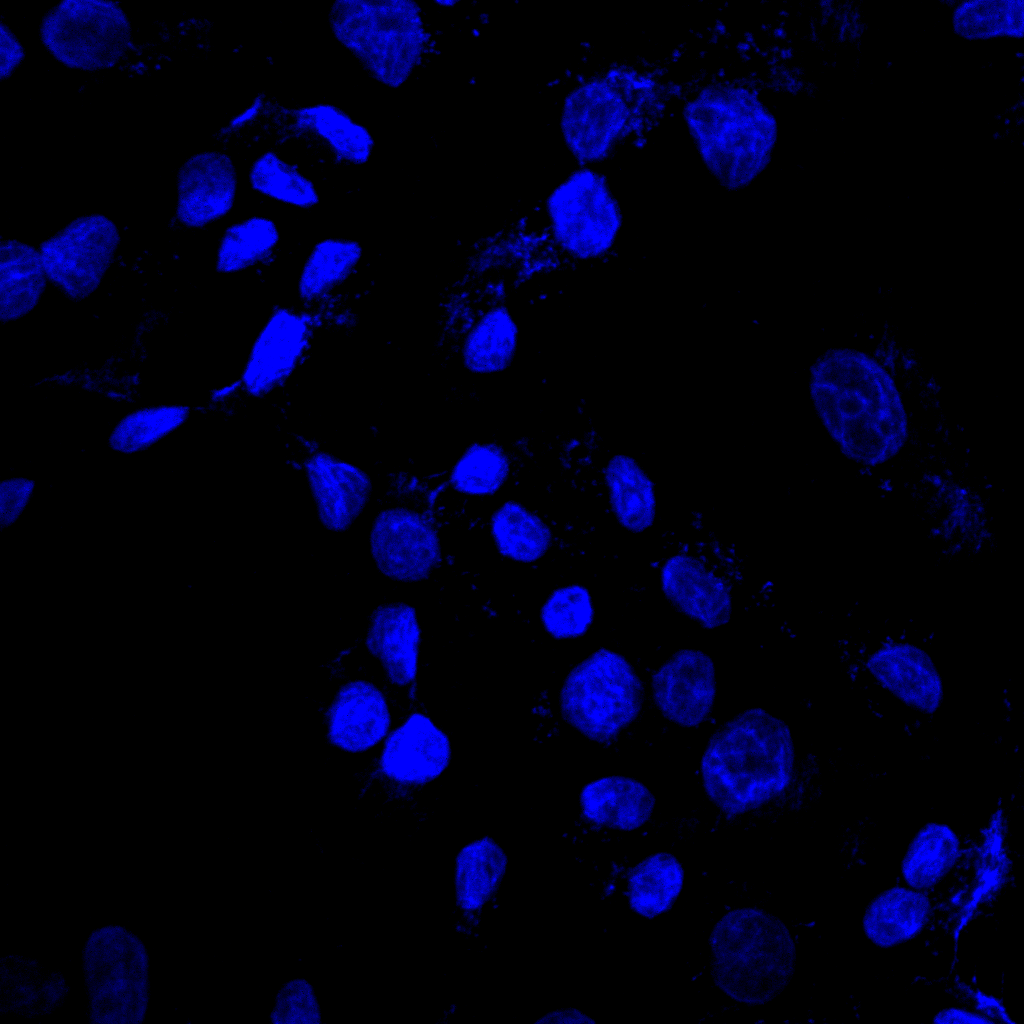

Supplement: Supplementary file 9 — Figure EV2 Source Data [file 44319_2025_581_MOESM9_ESM.zip › EV2/C/2hpi MOCK.tif]

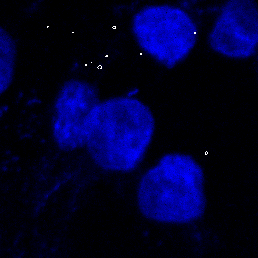

Supplement: Supplementary file 9 — Figure EV2 Source Data [file 44319_2025_581_MOESM9_ESM.zip › EV2/C/2hpi Vector zoom.tif]

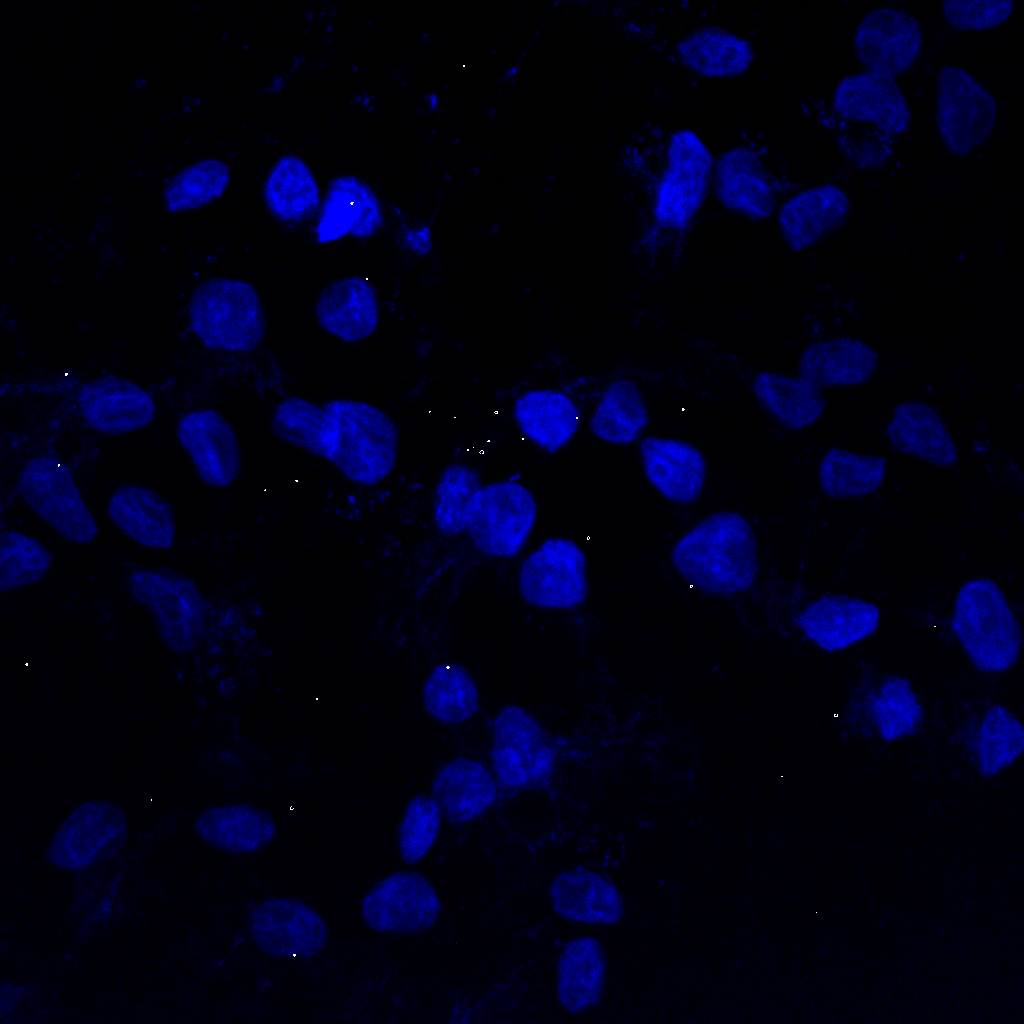

Supplement: Supplementary file 9 — Figure EV2 Source Data [file 44319_2025_581_MOESM9_ESM.zip › EV2/C/2hpi Vector.tif]

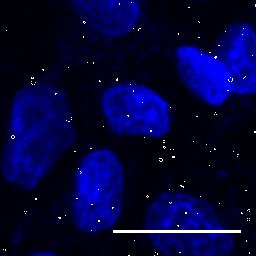

Supplement: Supplementary file 9 — Figure EV2 Source Data [file 44319_2025_581_MOESM9_ESM.zip › EV2/C/4hpi CA zoom.tif]

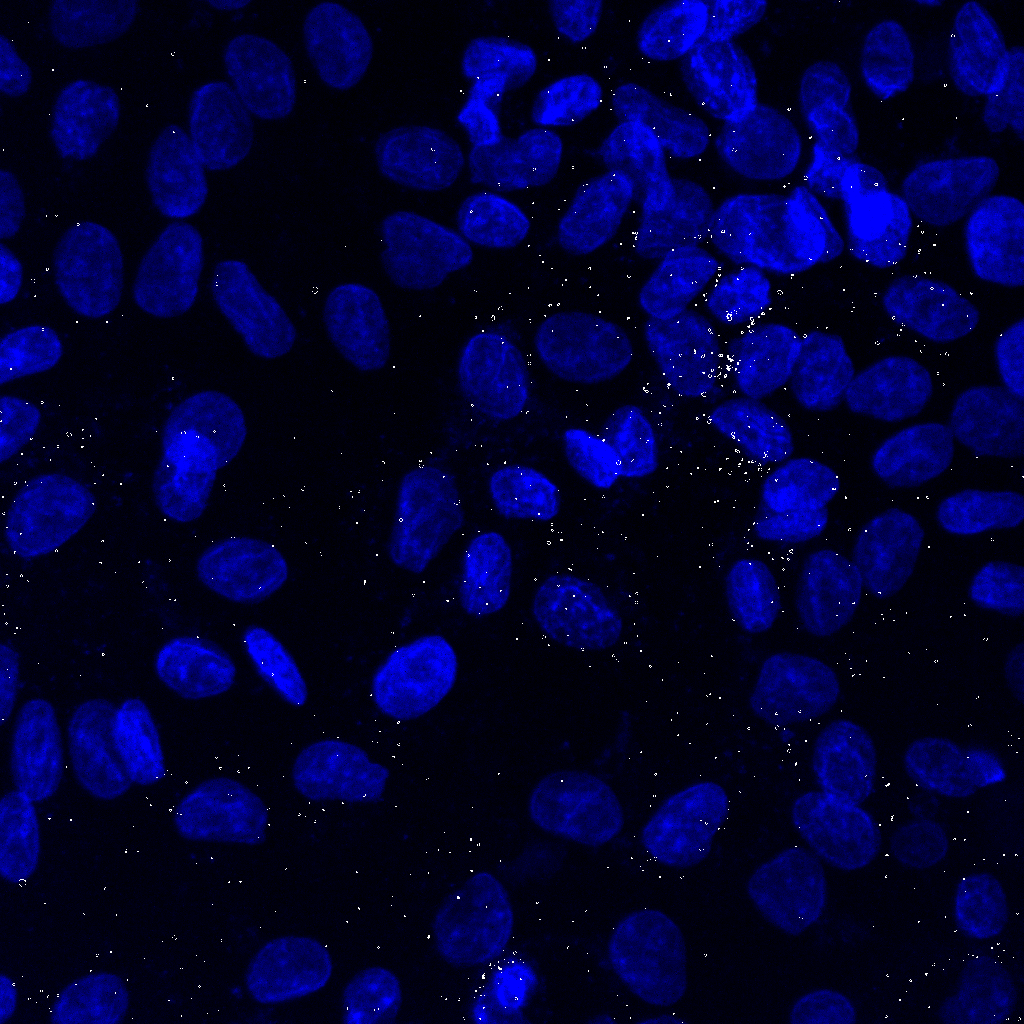

Supplement: Supplementary file 9 — Figure EV2 Source Data [file 44319_2025_581_MOESM9_ESM.zip › EV2/C/4hpi CA.tif]

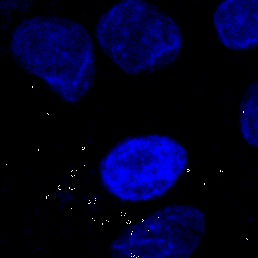

Supplement: Supplementary file 9 — Figure EV2 Source Data [file 44319_2025_581_MOESM9_ESM.zip › EV2/C/4hpi DN zoom.tif]

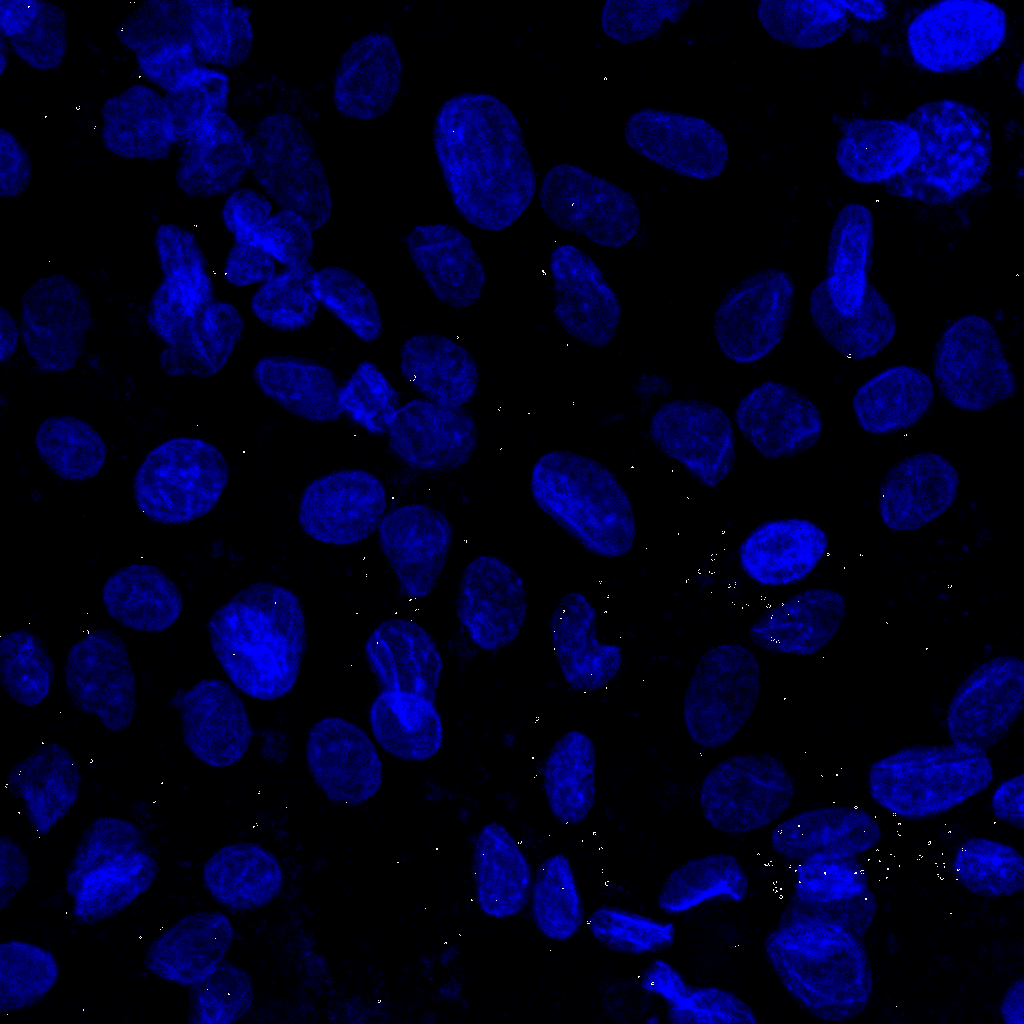

Supplement: Supplementary file 9 — Figure EV2 Source Data [file 44319_2025_581_MOESM9_ESM.zip › EV2/C/4hpi DN.tif]

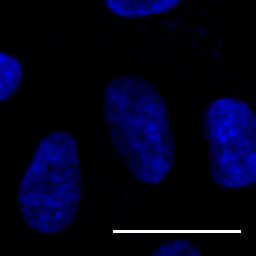

Supplement: Supplementary file 9 — Figure EV2 Source Data [file 44319_2025_581_MOESM9_ESM.zip › EV2/C/4hpi MOCK zoom.tif]

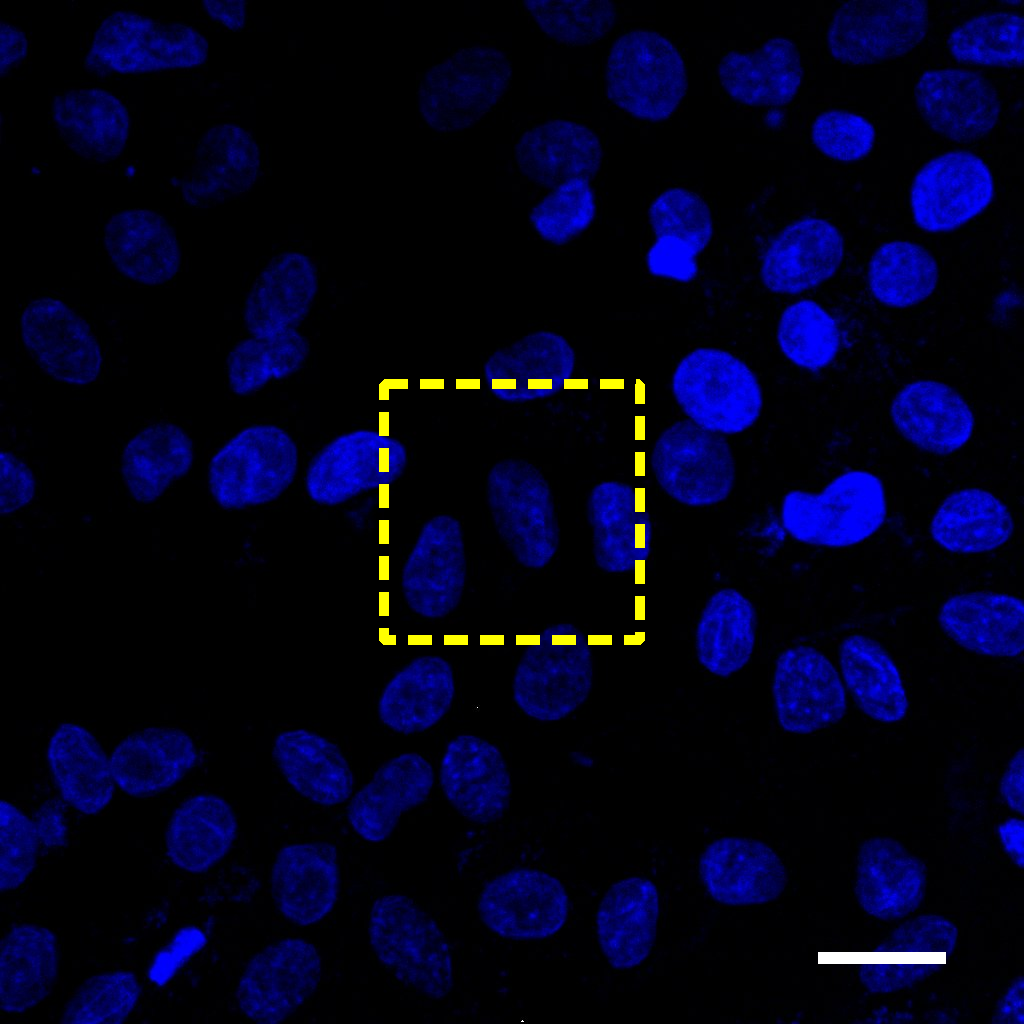

Supplement: Supplementary file 9 — Figure EV2 Source Data [file 44319_2025_581_MOESM9_ESM.zip › EV2/C/4hpi MOCK.tif]

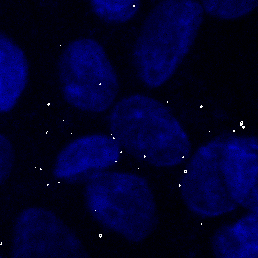

Supplement: Supplementary file 9 — Figure EV2 Source Data [file 44319_2025_581_MOESM9_ESM.zip › EV2/C/4hpi Vector zoom.tif]

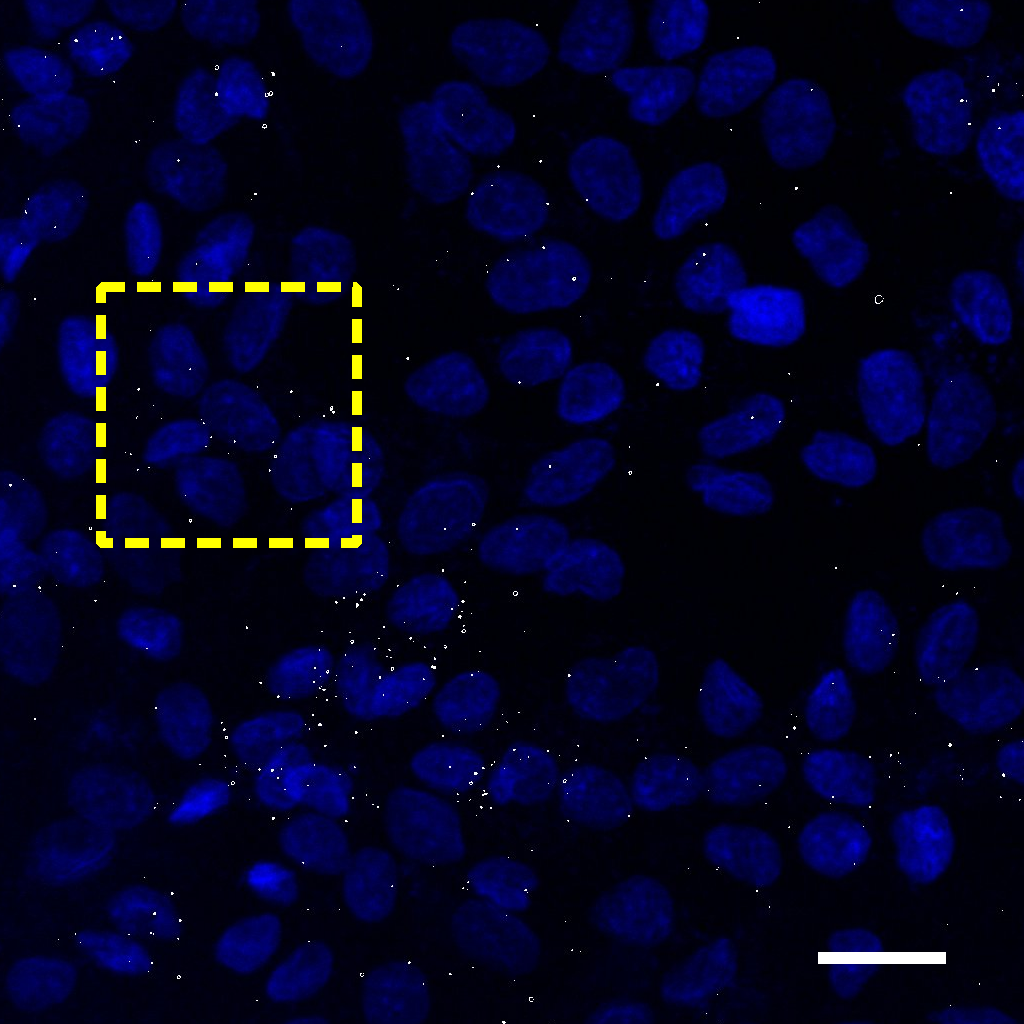

Supplement: Supplementary file 9 — Figure EV2 Source Data [file 44319_2025_581_MOESM9_ESM.zip › EV2/C/4hpi Vector.tif]

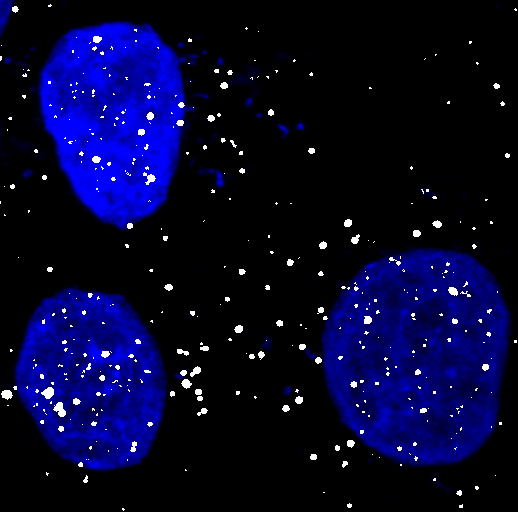

Supplement: Supplementary file 9 — Figure EV2 Source Data [file 44319_2025_581_MOESM9_ESM.zip › EV2/C/6hpi CA zoom.tif]

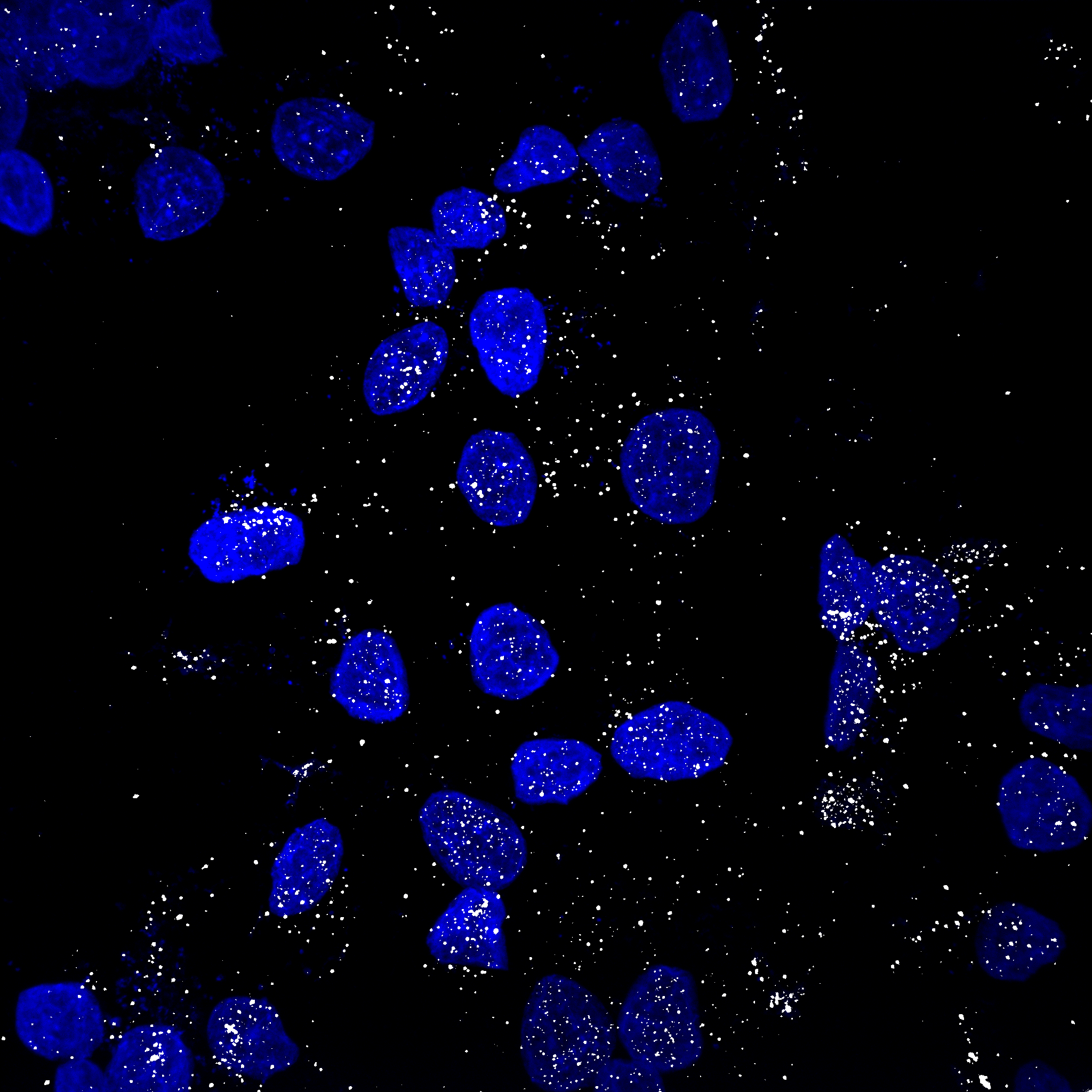

Supplement: Supplementary file 9 — Figure EV2 Source Data [file 44319_2025_581_MOESM9_ESM.zip › EV2/C/6hpi CA.tif]

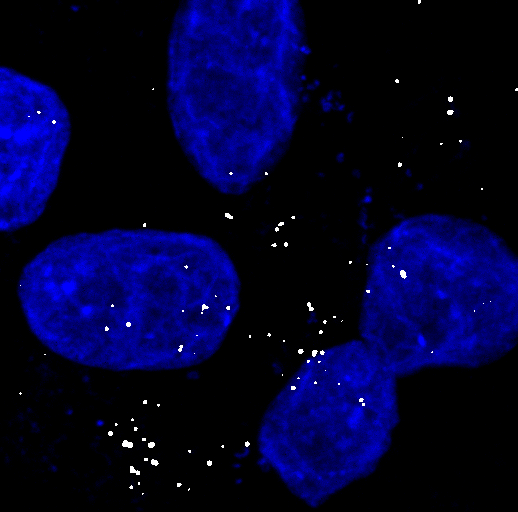

Supplement: Supplementary file 9 — Figure EV2 Source Data [file 44319_2025_581_MOESM9_ESM.zip › EV2/C/6hpi DN zoom.tif]

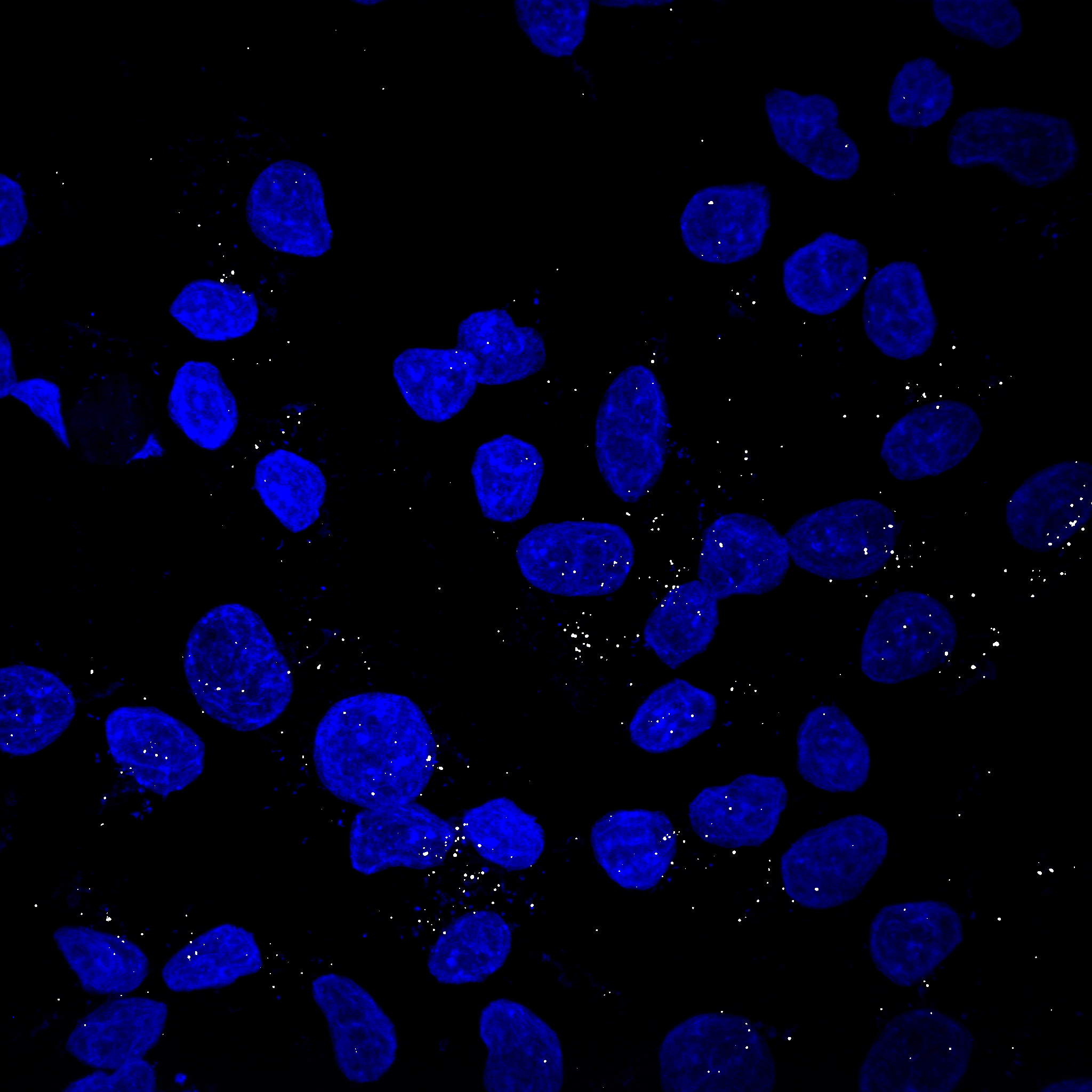

Supplement: Supplementary file 9 — Figure EV2 Source Data [file 44319_2025_581_MOESM9_ESM.zip › EV2/C/6hpi DN.tif]

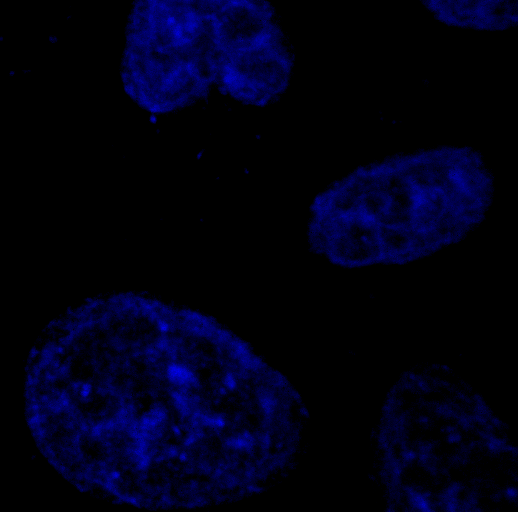

Supplement: Supplementary file 9 — Figure EV2 Source Data [file 44319_2025_581_MOESM9_ESM.zip › EV2/C/6hpi MOCK zoom.tif]

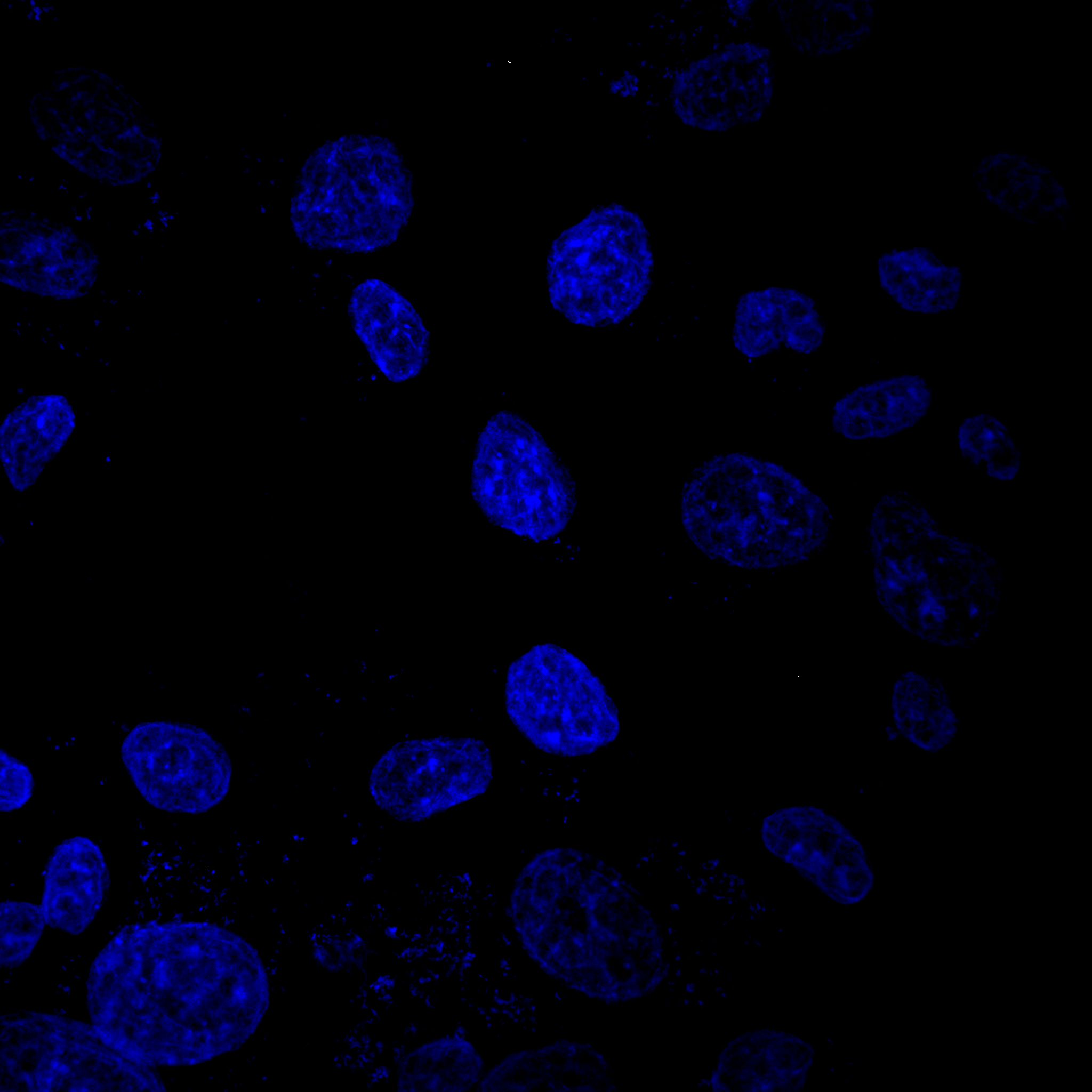

Supplement: Supplementary file 9 — Figure EV2 Source Data [file 44319_2025_581_MOESM9_ESM.zip › EV2/C/6hpi MOCK.tif]

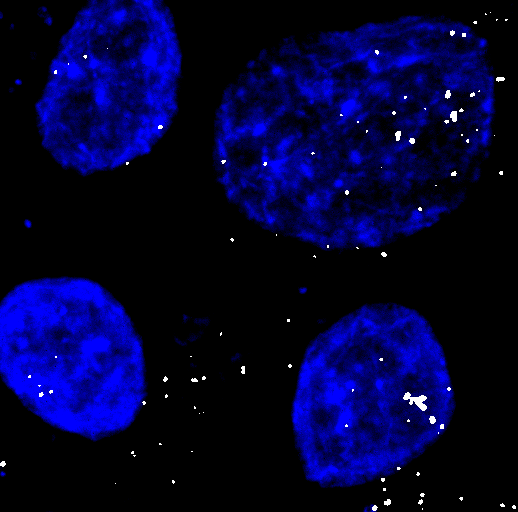

Supplement: Supplementary file 9 — Figure EV2 Source Data [file 44319_2025_581_MOESM9_ESM.zip › EV2/C/6hpi Vector zoom.tif]

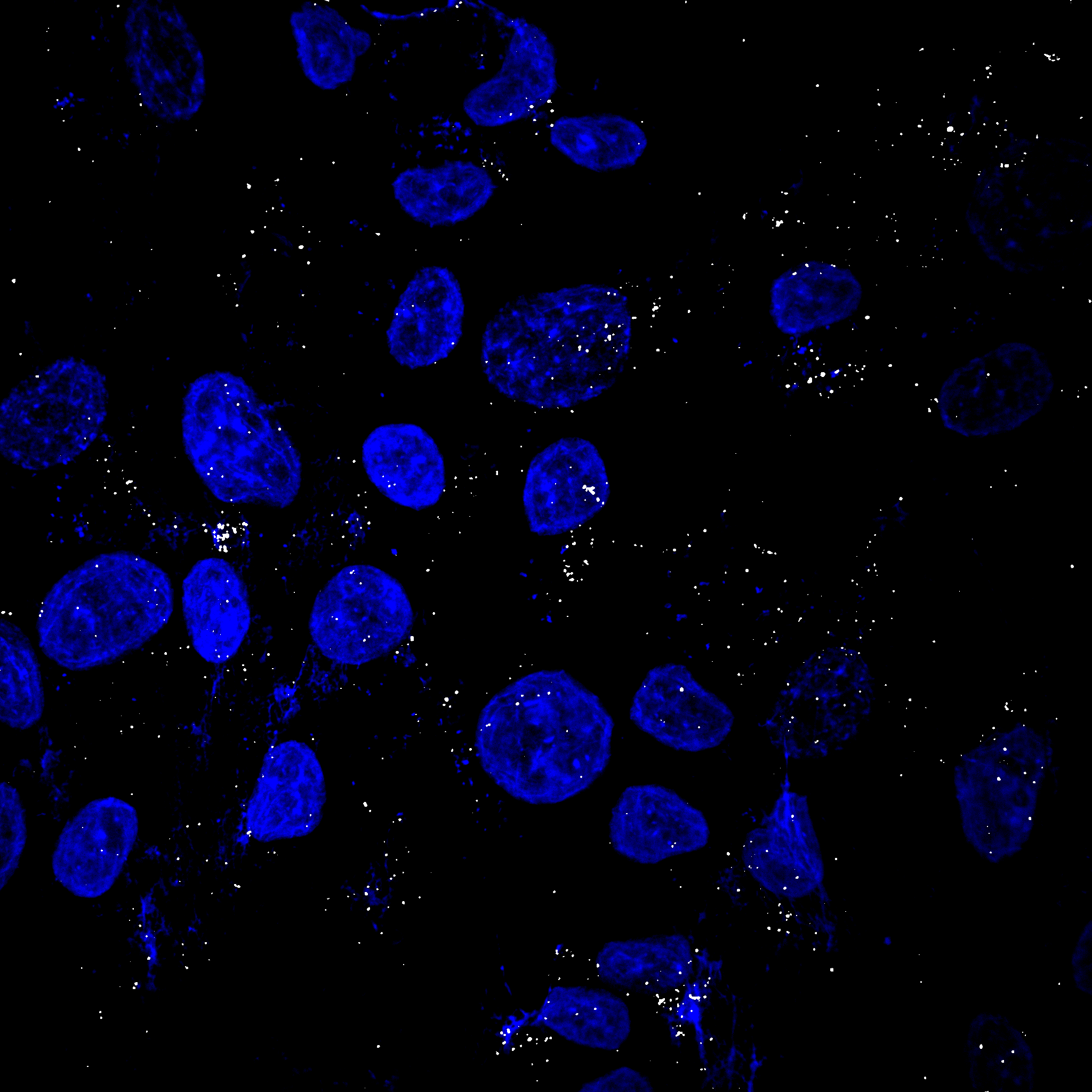

Supplement: Supplementary file 9 — Figure EV2 Source Data [file 44319_2025_581_MOESM9_ESM.zip › EV2/C/6hpi Vector.tif]

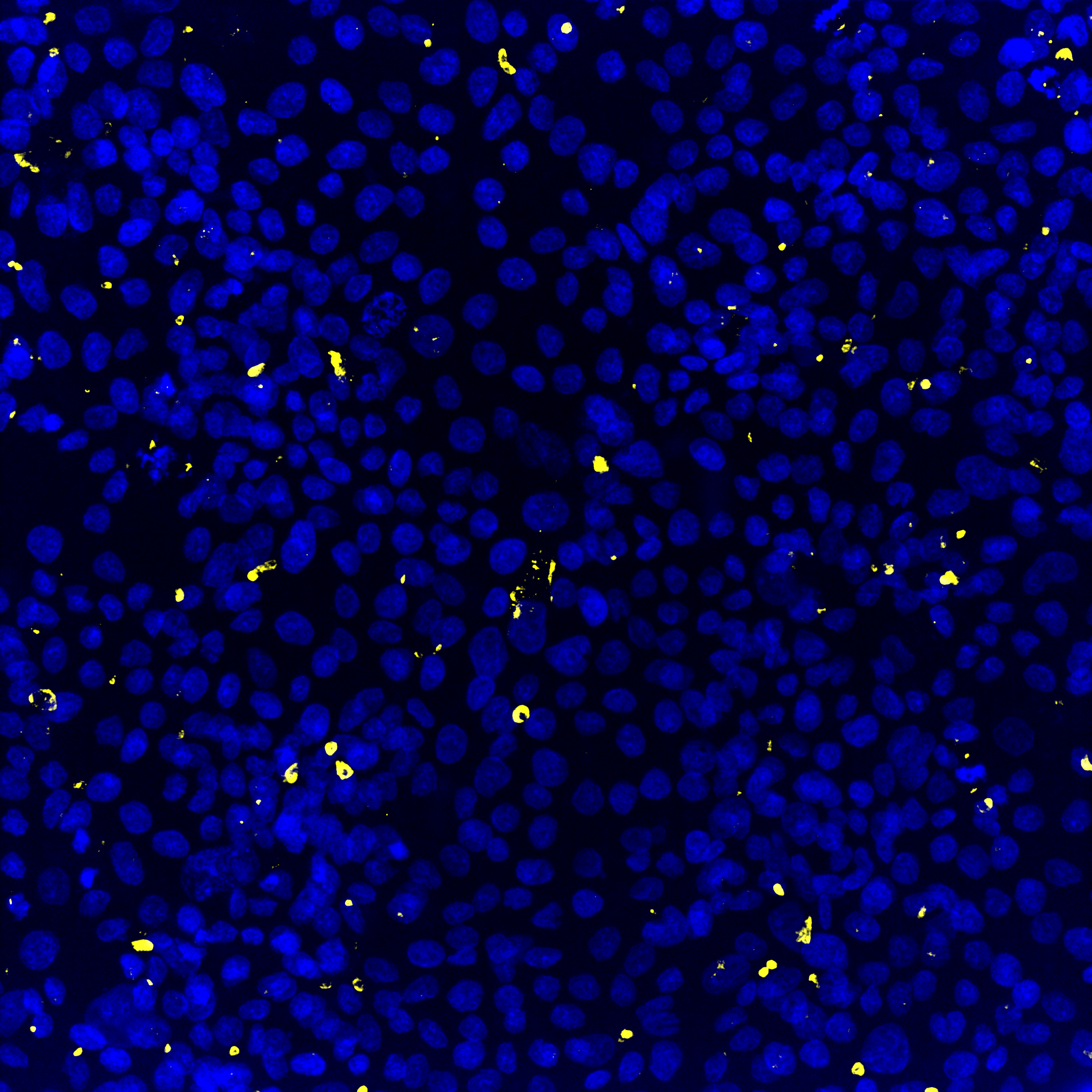

Supplement: Supplementary file 10 — Figure EV3 Source Data [file 44319_2025_581_MOESM10_ESM.zip › EV3/E/CDC42-CA.tif]

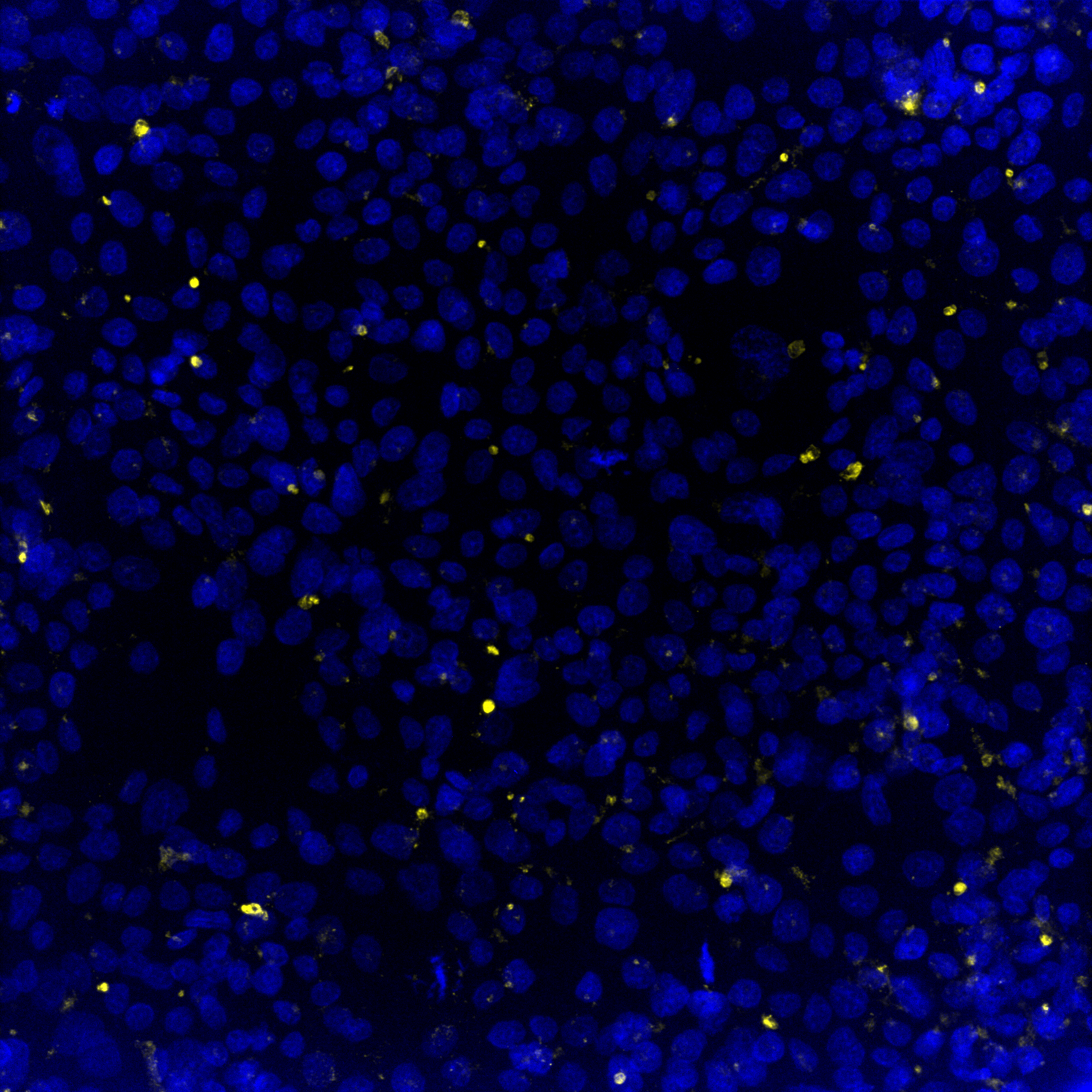

Supplement: Supplementary file 10 — Figure EV3 Source Data [file 44319_2025_581_MOESM10_ESM.zip › EV3/E/CDC42-DN.tif]

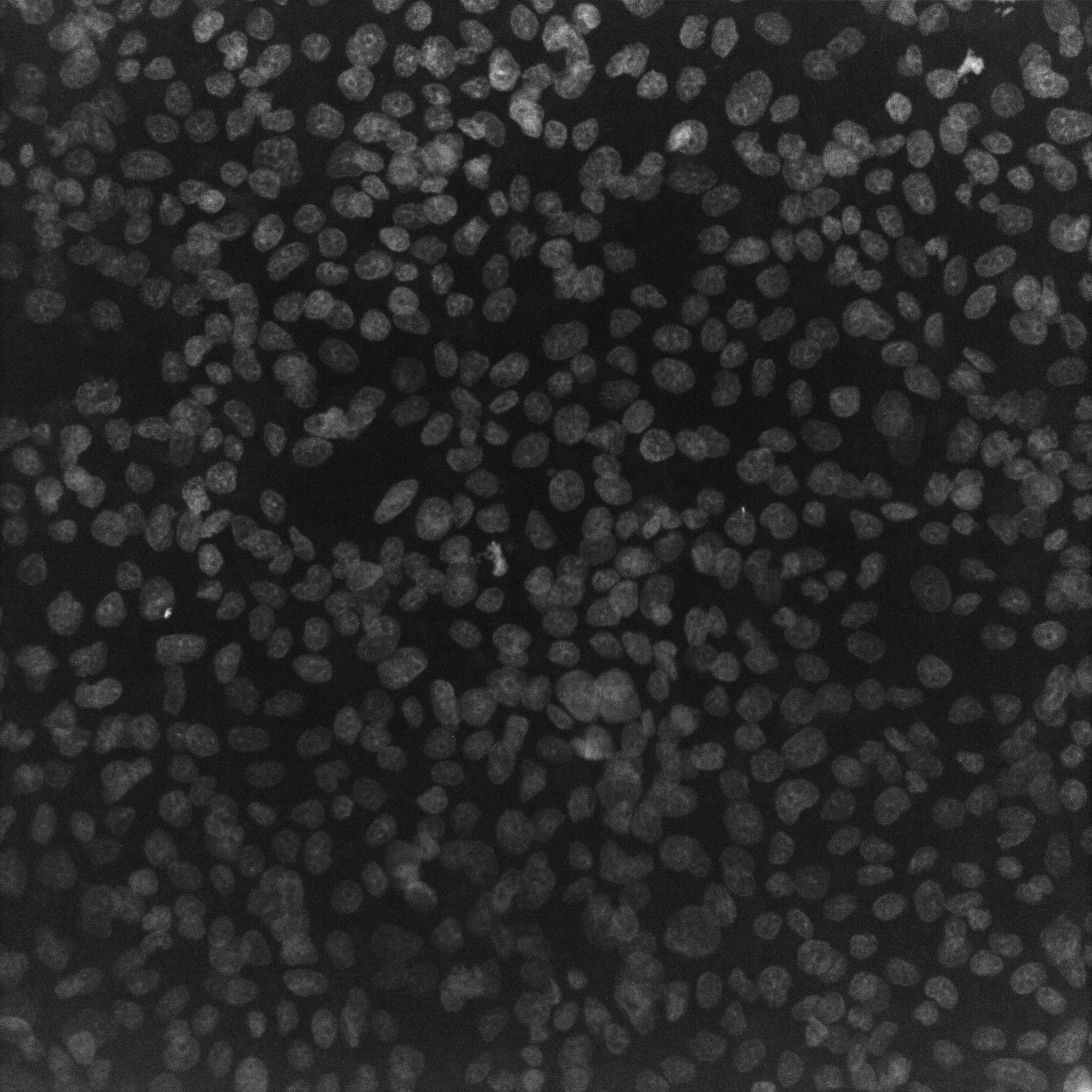

Supplement: Supplementary file 10 — Figure EV3 Source Data [file 44319_2025_581_MOESM10_ESM.zip › EV3/E/Vector.tif]

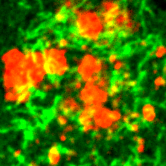

Supplement: Supplementary file 11 — Figure EV4 Source Data [file 44319_2025_581_MOESM11_ESM.zip › EV4/B/Cdc42 CA Bottom.tif]

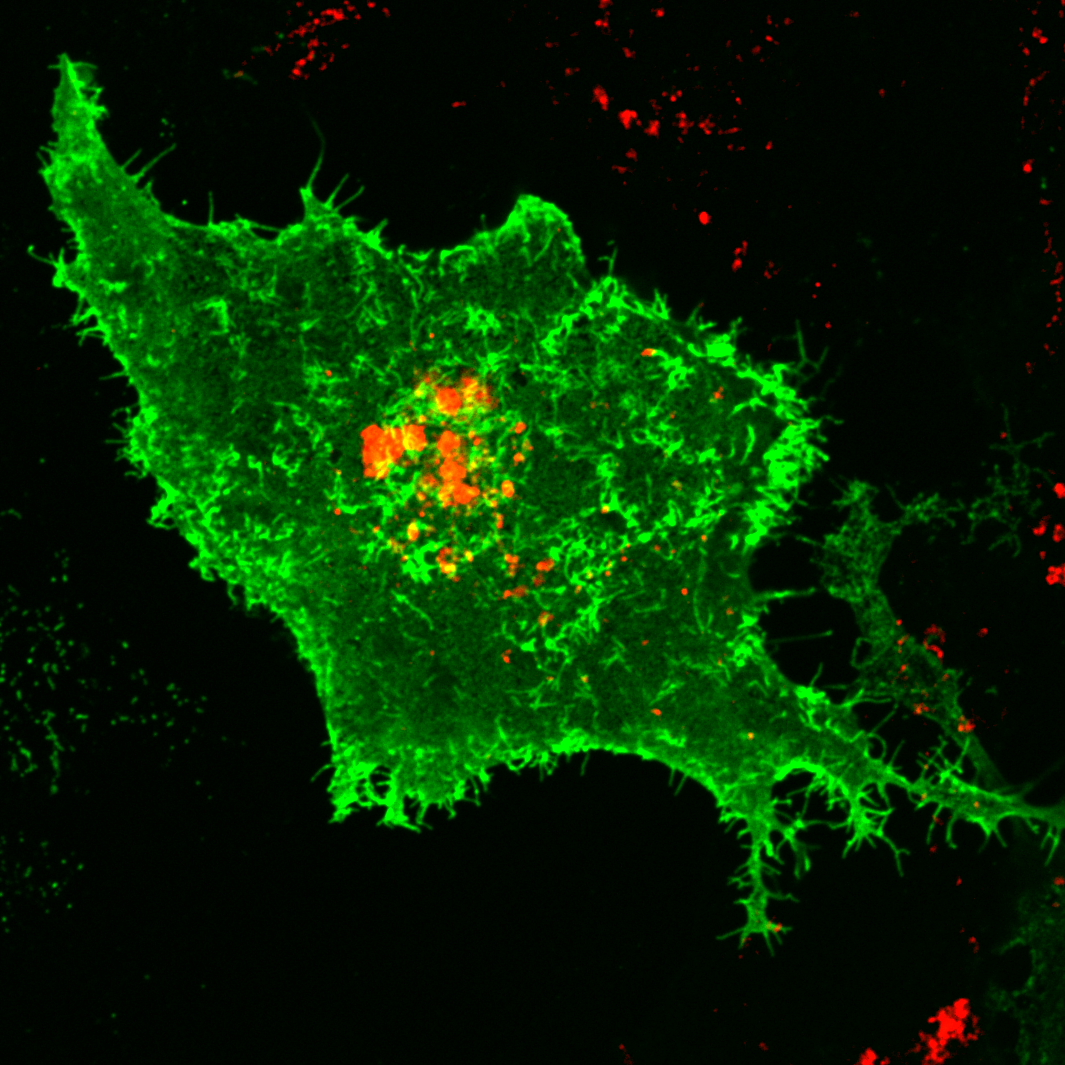

Supplement: Supplementary file 11 — Figure EV4 Source Data [file 44319_2025_581_MOESM11_ESM.zip › EV4/B/Cdc42 CA Top.tif]

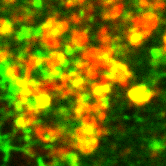

Supplement: Supplementary file 11 — Figure EV4 Source Data [file 44319_2025_581_MOESM11_ESM.zip › EV4/B/Cdc42 DN Bottom.tif]

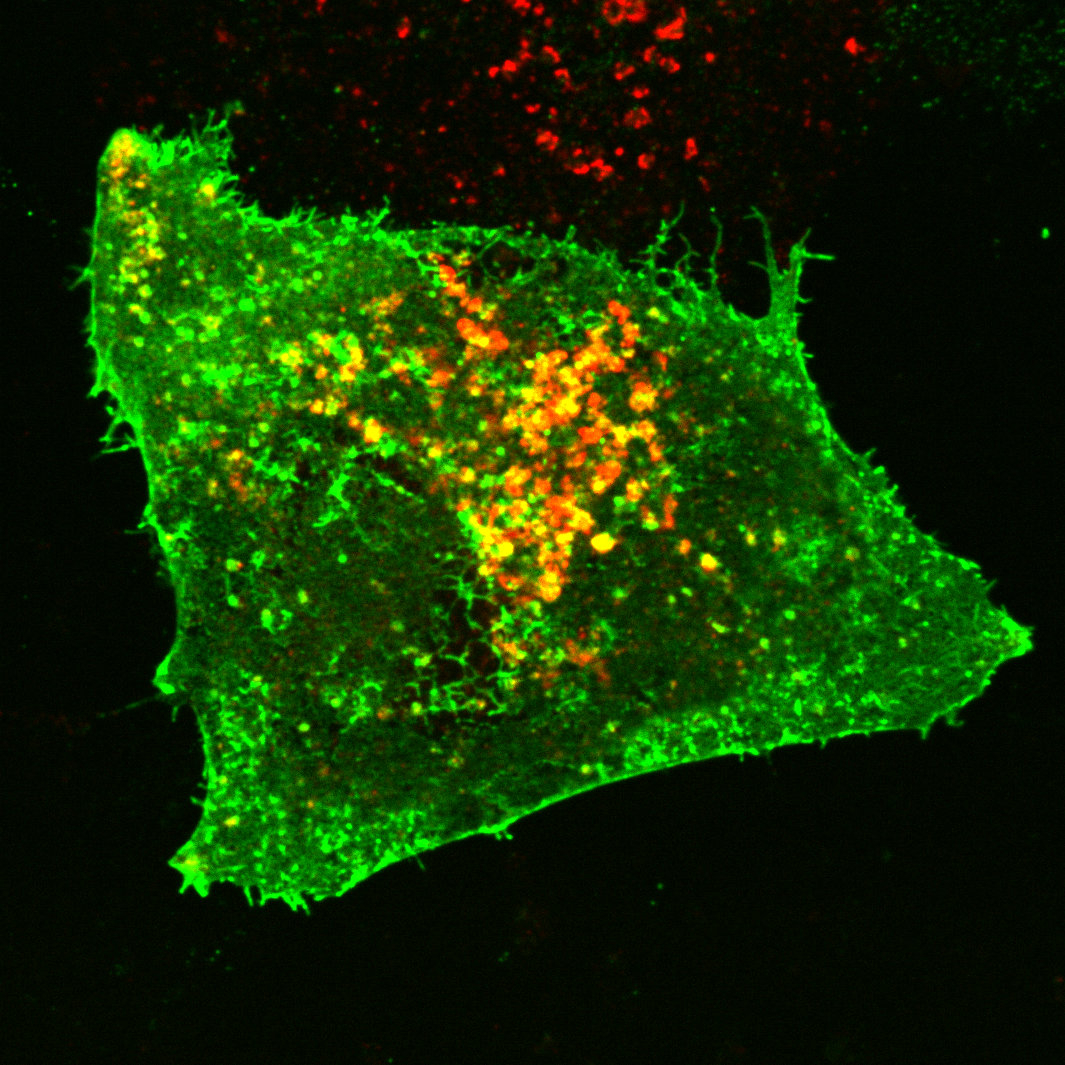

Supplement: Supplementary file 11 — Figure EV4 Source Data [file 44319_2025_581_MOESM11_ESM.zip › EV4/B/Cdc42 DN Top.tif]

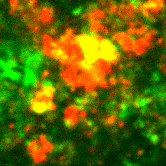

Supplement: Supplementary file 11 — Figure EV4 Source Data [file 44319_2025_581_MOESM11_ESM.zip › EV4/B/Cdc42 WT Bottom.tif]

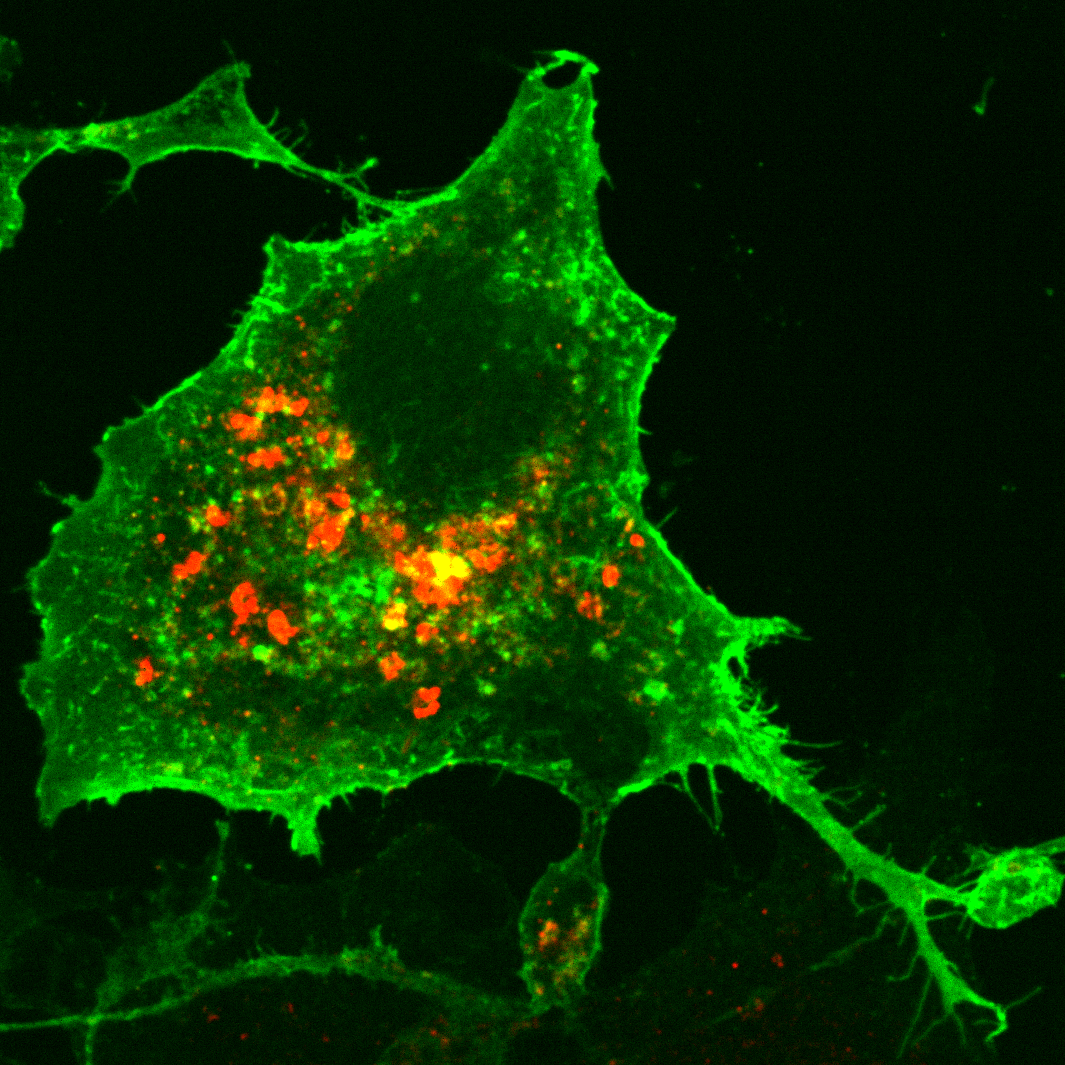

Supplement: Supplementary file 11 — Figure EV4 Source Data [file 44319_2025_581_MOESM11_ESM.zip › EV4/B/Cdc42 WT Top.tif]

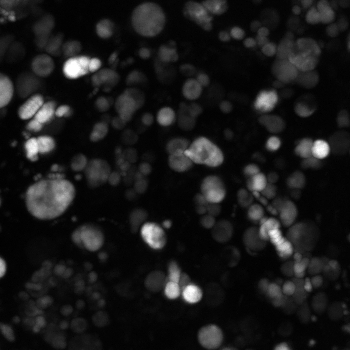

Supplement: Supplementary file 12 — Figure EV5 Source Data [file 44319_2025_581_MOESM12_ESM.zip › EV5/A/CDC42-CA bottom.tif]

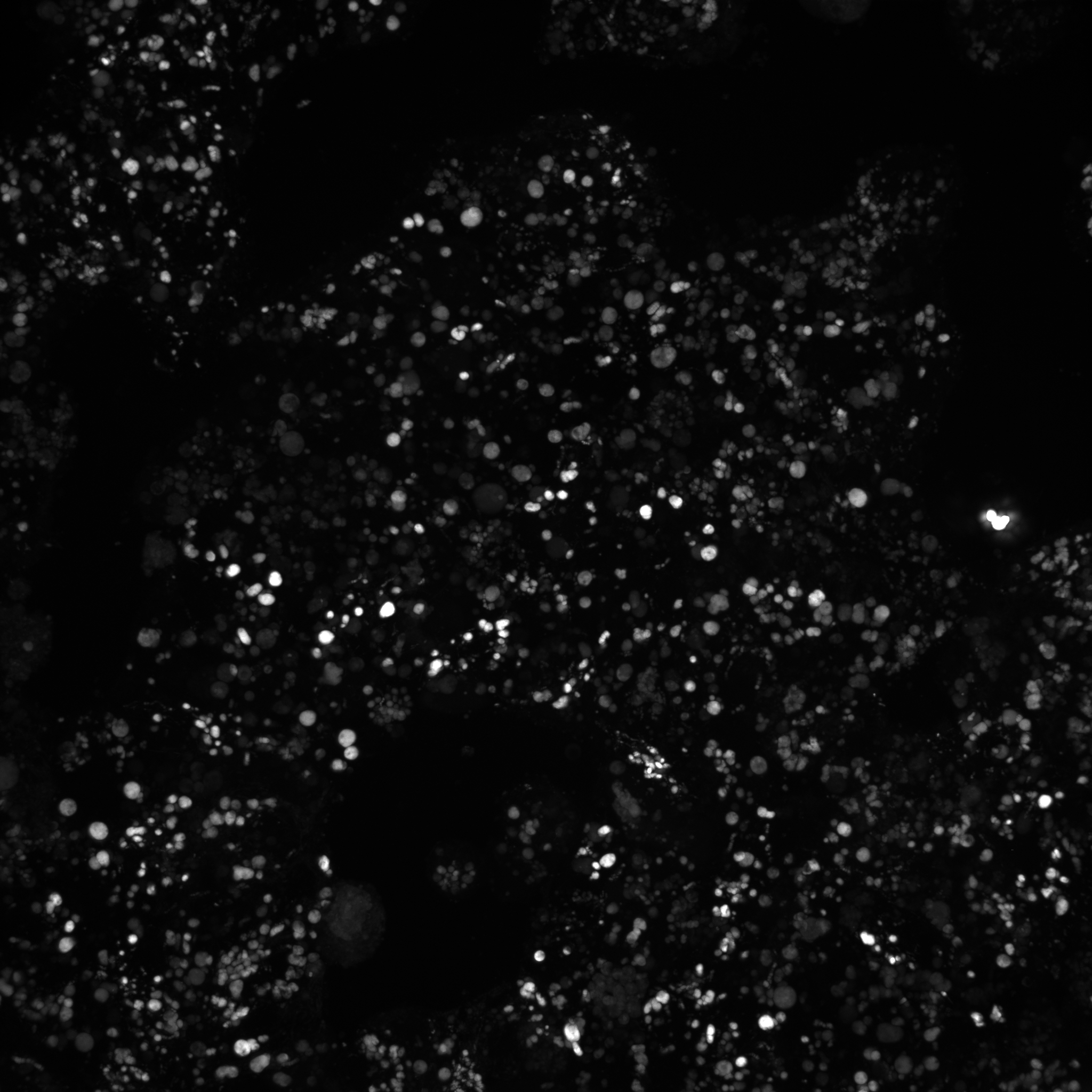

Supplement: Supplementary file 12 — Figure EV5 Source Data [file 44319_2025_581_MOESM12_ESM.zip › EV5/A/CDC42-CA top.tif]

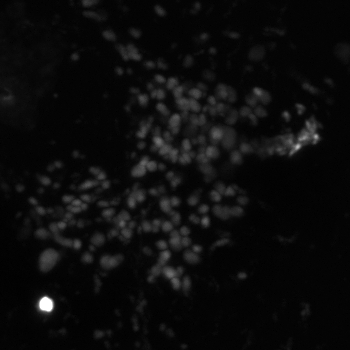

Supplement: Supplementary file 12 — Figure EV5 Source Data [file 44319_2025_581_MOESM12_ESM.zip › EV5/A/CDC42-DN bottom.tif]

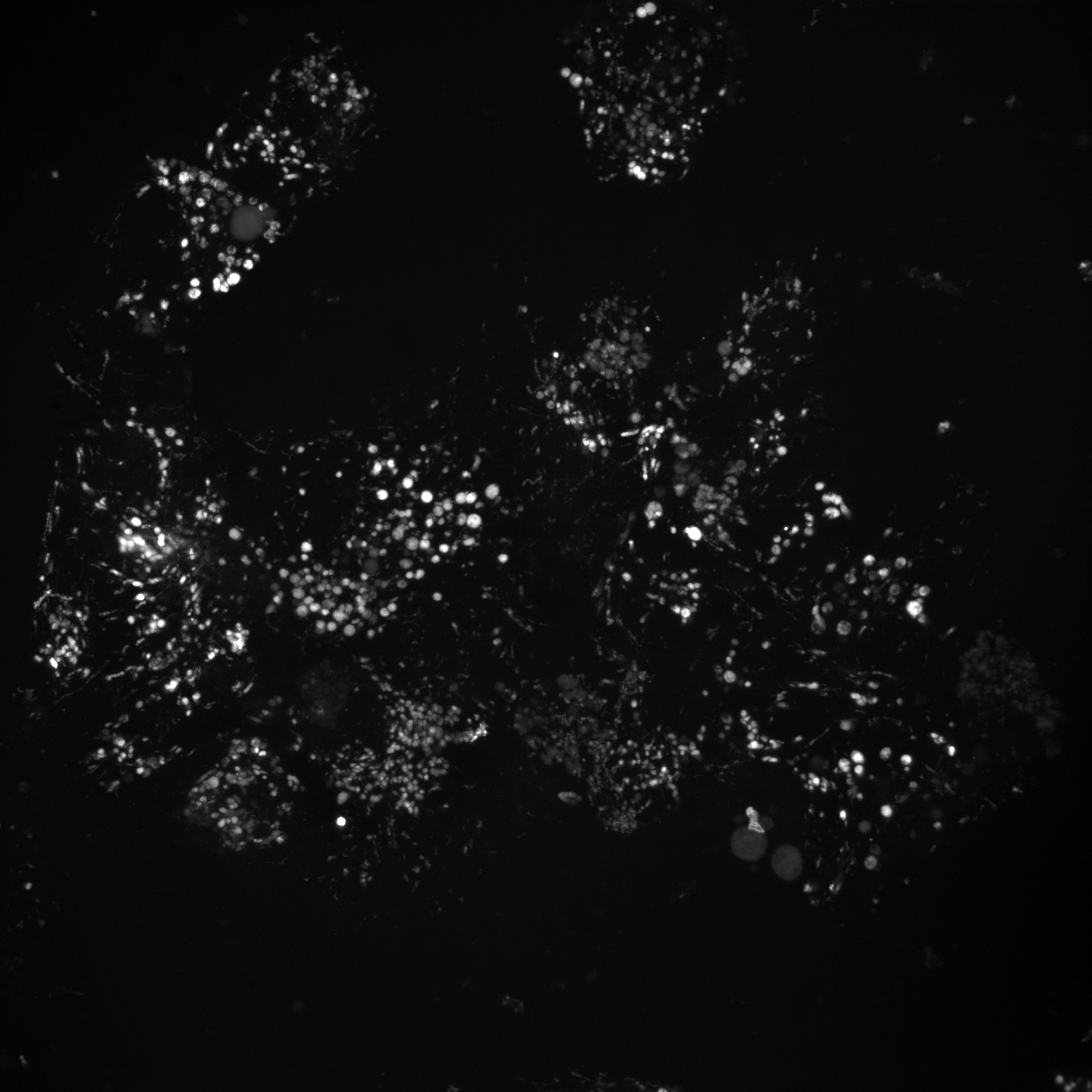

Supplement: Supplementary file 12 — Figure EV5 Source Data [file 44319_2025_581_MOESM12_ESM.zip › EV5/A/CDC42-DN top.tif]

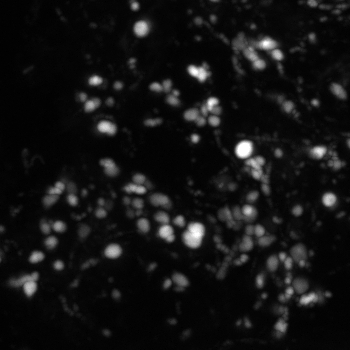

Supplement: Supplementary file 12 — Figure EV5 Source Data [file 44319_2025_581_MOESM12_ESM.zip › EV5/A/Vector bottom.tif]

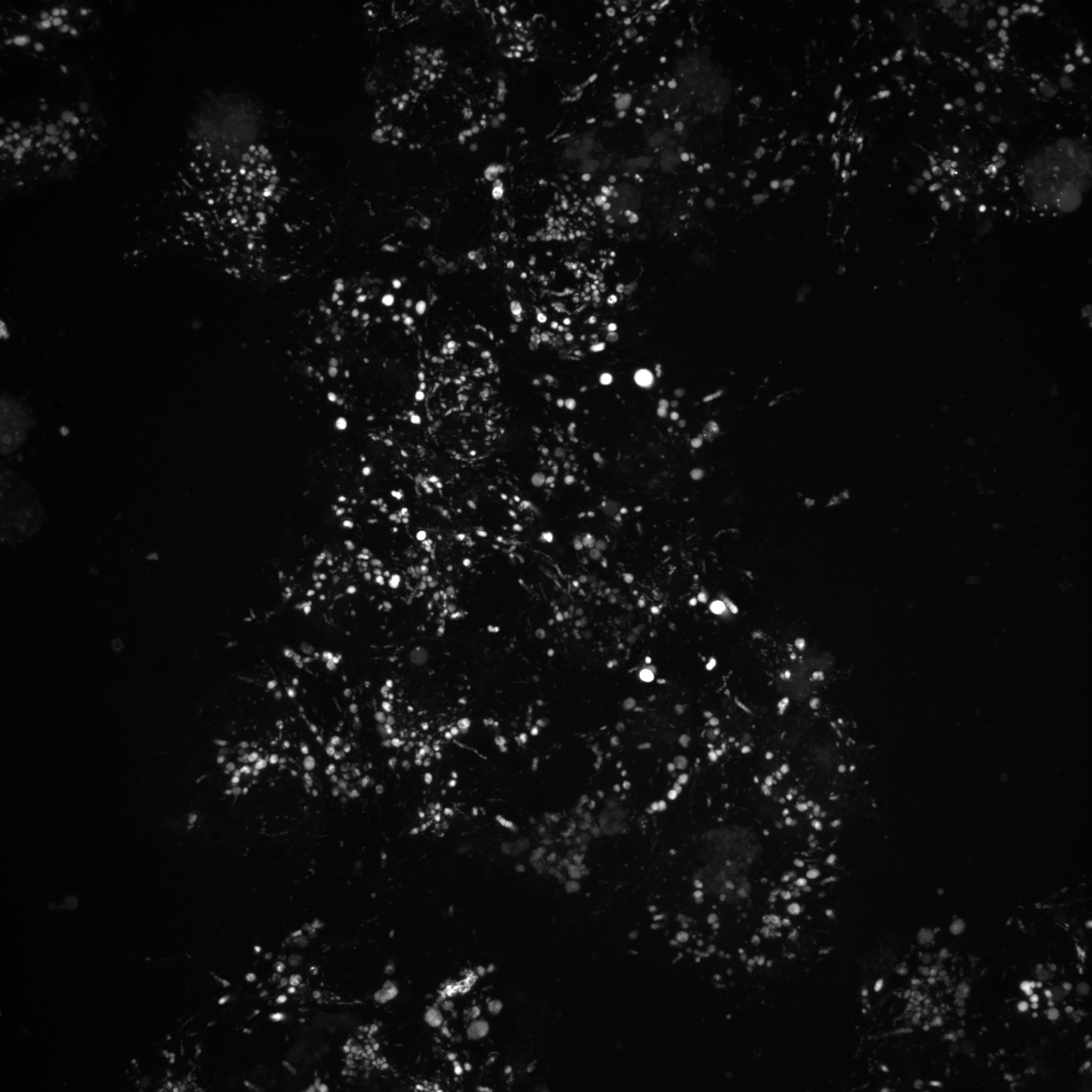

Supplement: Supplementary file 12 — Figure EV5 Source Data [file 44319_2025_581_MOESM12_ESM.zip › EV5/A/Vector-top.tif]

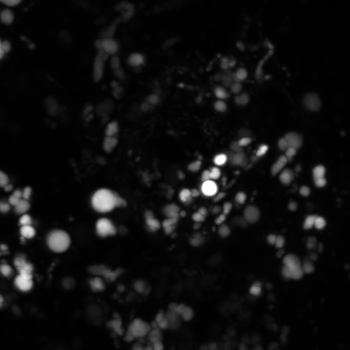

Supplement: Supplementary file 12 — Figure EV5 Source Data [file 44319_2025_581_MOESM12_ESM.zip › EV5/C/Bradykinin bottom.tif]

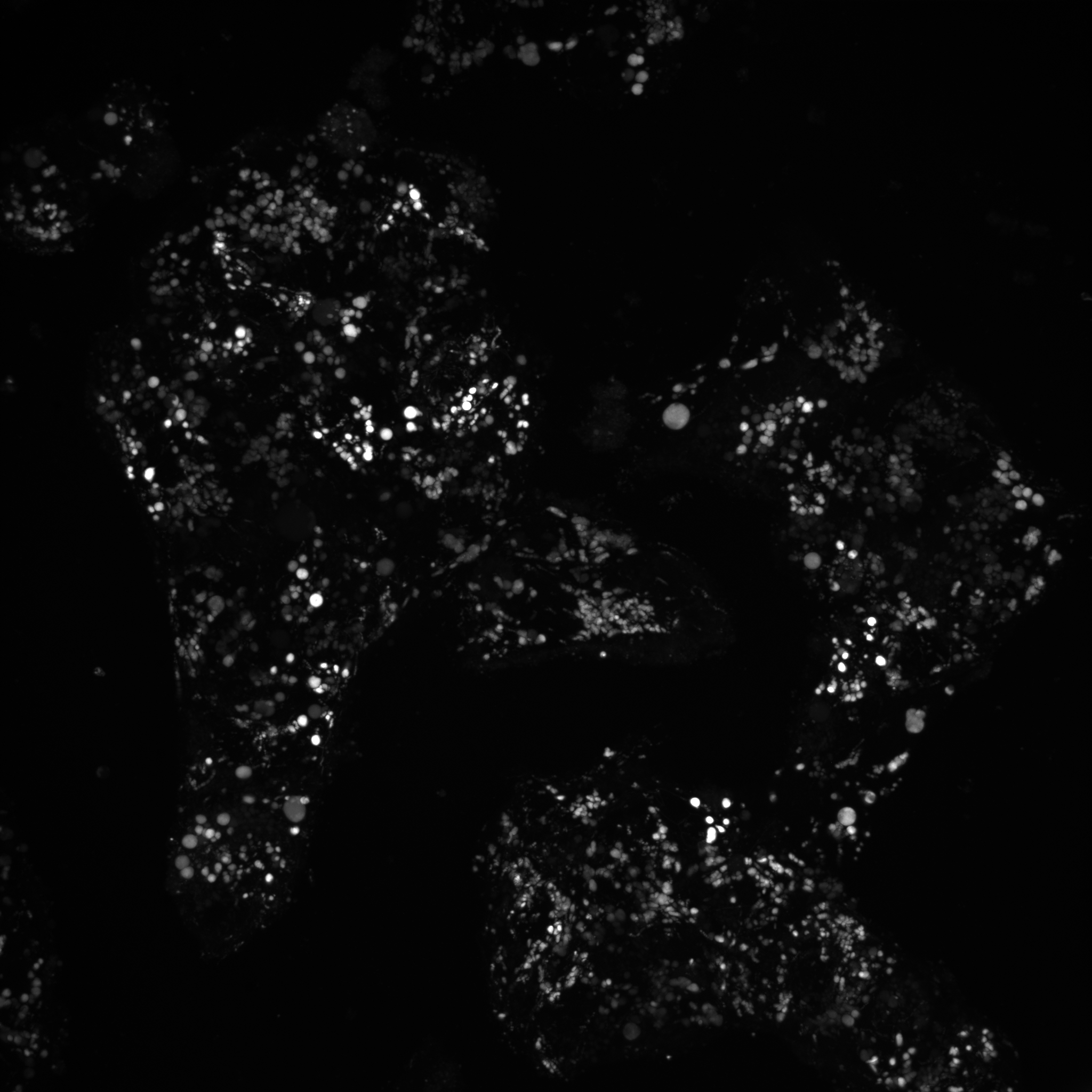

Supplement: Supplementary file 12 — Figure EV5 Source Data [file 44319_2025_581_MOESM12_ESM.zip › EV5/C/Bradykinin top.tif]

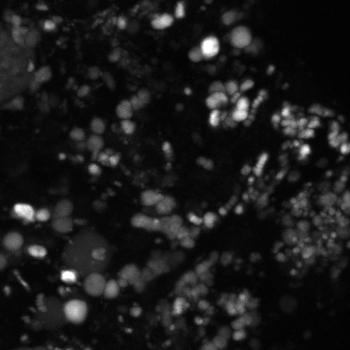

Supplement: Supplementary file 12 — Figure EV5 Source Data [file 44319_2025_581_MOESM12_ESM.zip › EV5/C/ML141 bottom.tif]

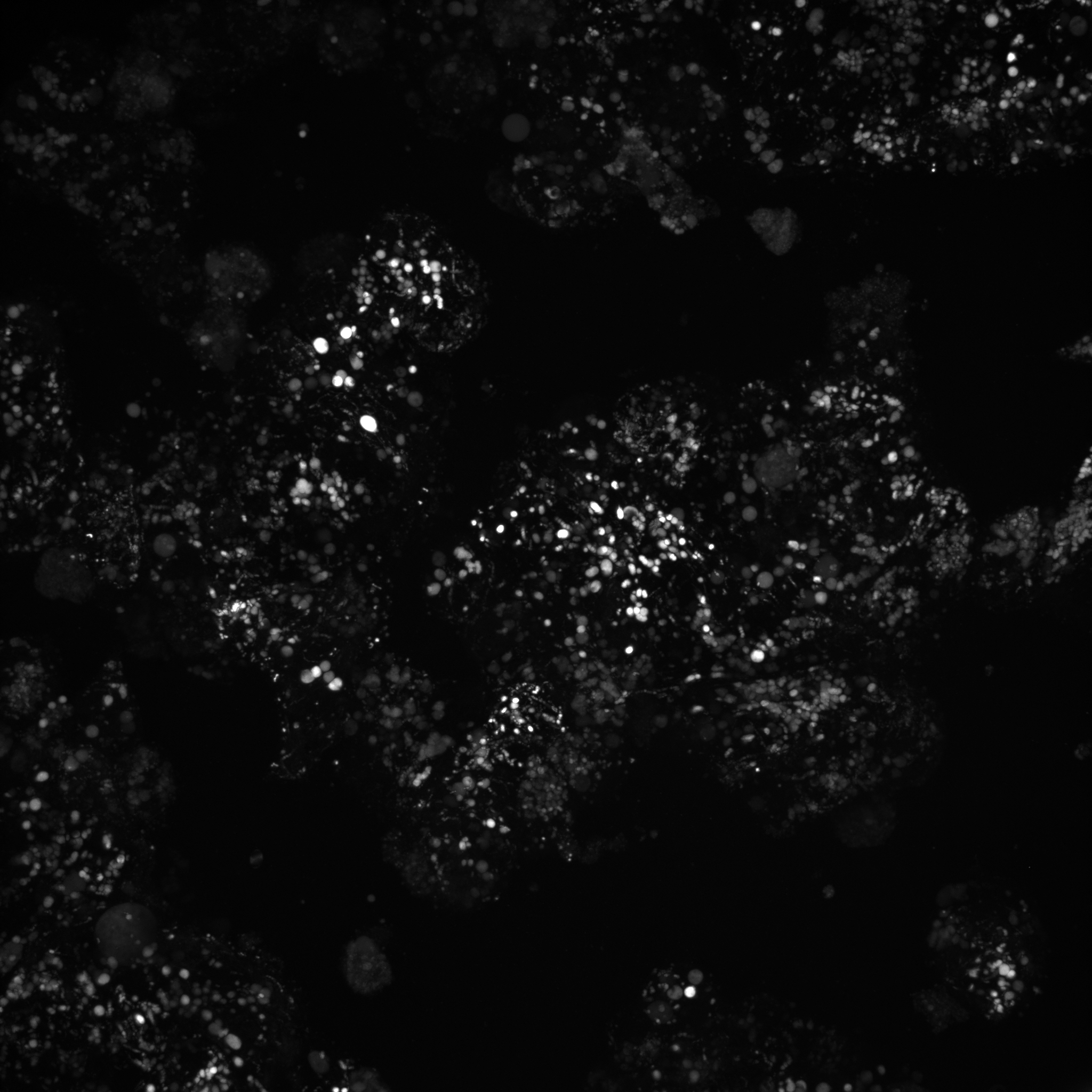

Supplement: Supplementary file 12 — Figure EV5 Source Data [file 44319_2025_581_MOESM12_ESM.zip › EV5/C/ML141 top.tif]

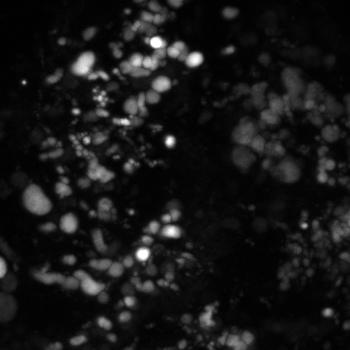

Supplement: Supplementary file 12 — Figure EV5 Source Data [file 44319_2025_581_MOESM12_ESM.zip › EV5/C/Untreatment bottom.tif]

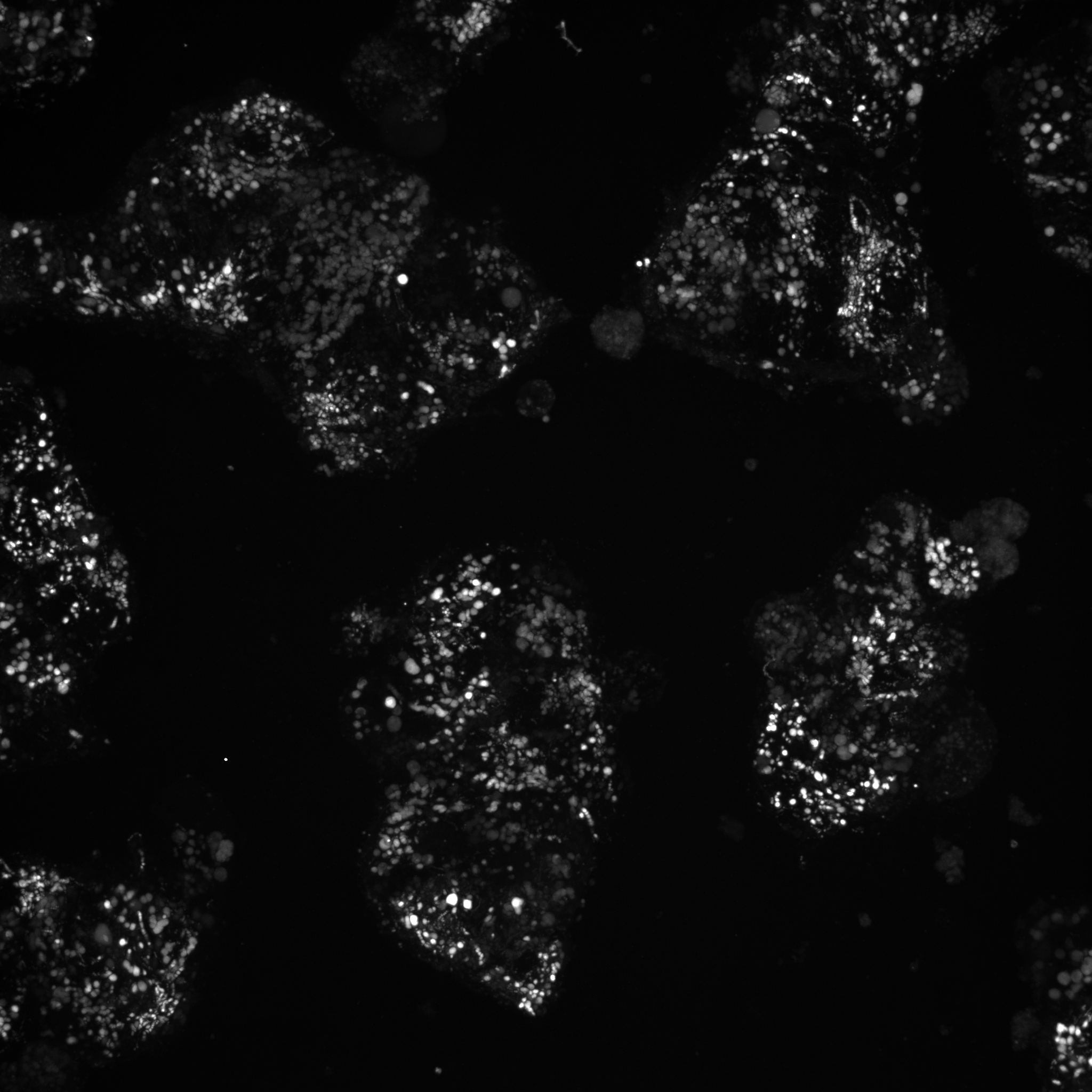

Supplement: Supplementary file 12 — Figure EV5 Source Data [file 44319_2025_581_MOESM12_ESM.zip › EV5/C/Untreatment top.tif]

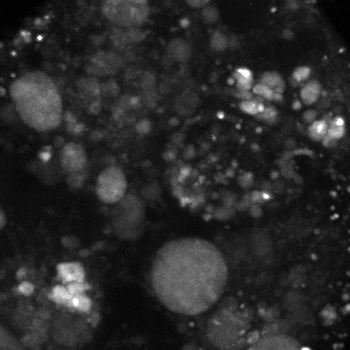

Supplement: Supplementary file 12 — Figure EV5 Source Data [file 44319_2025_581_MOESM12_ESM.zip › EV5/G/EIPA bottom.tif]

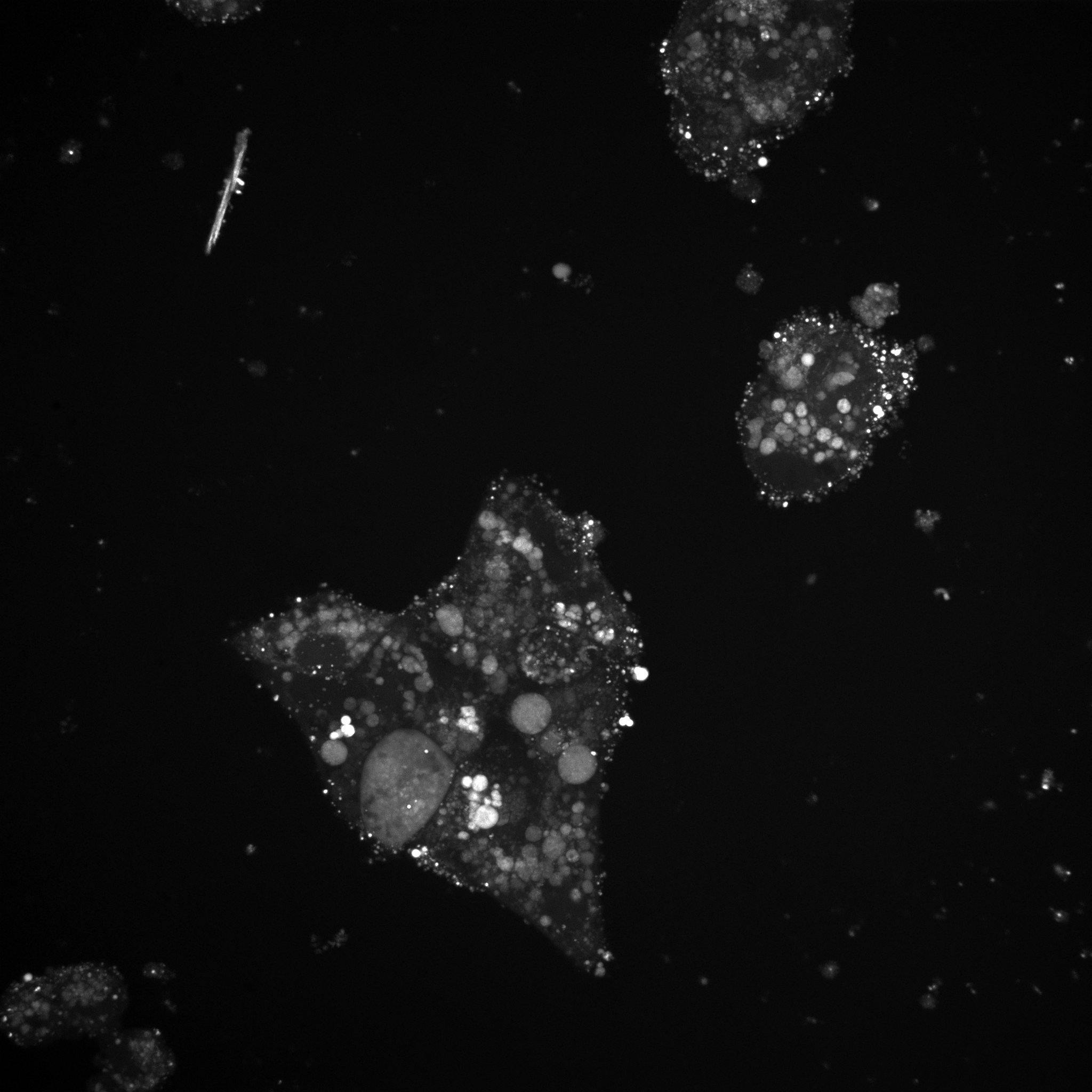

Supplement: Supplementary file 12 — Figure EV5 Source Data [file 44319_2025_581_MOESM12_ESM.zip › EV5/G/EIPA top.tif]

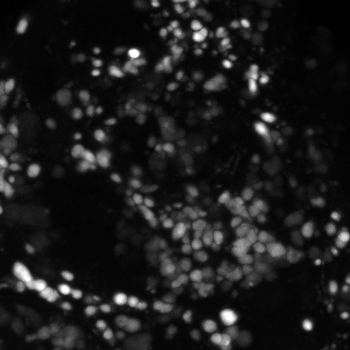

Supplement: Supplementary file 12 — Figure EV5 Source Data [file 44319_2025_581_MOESM12_ESM.zip › EV5/G/Untreatment bottom.tif]

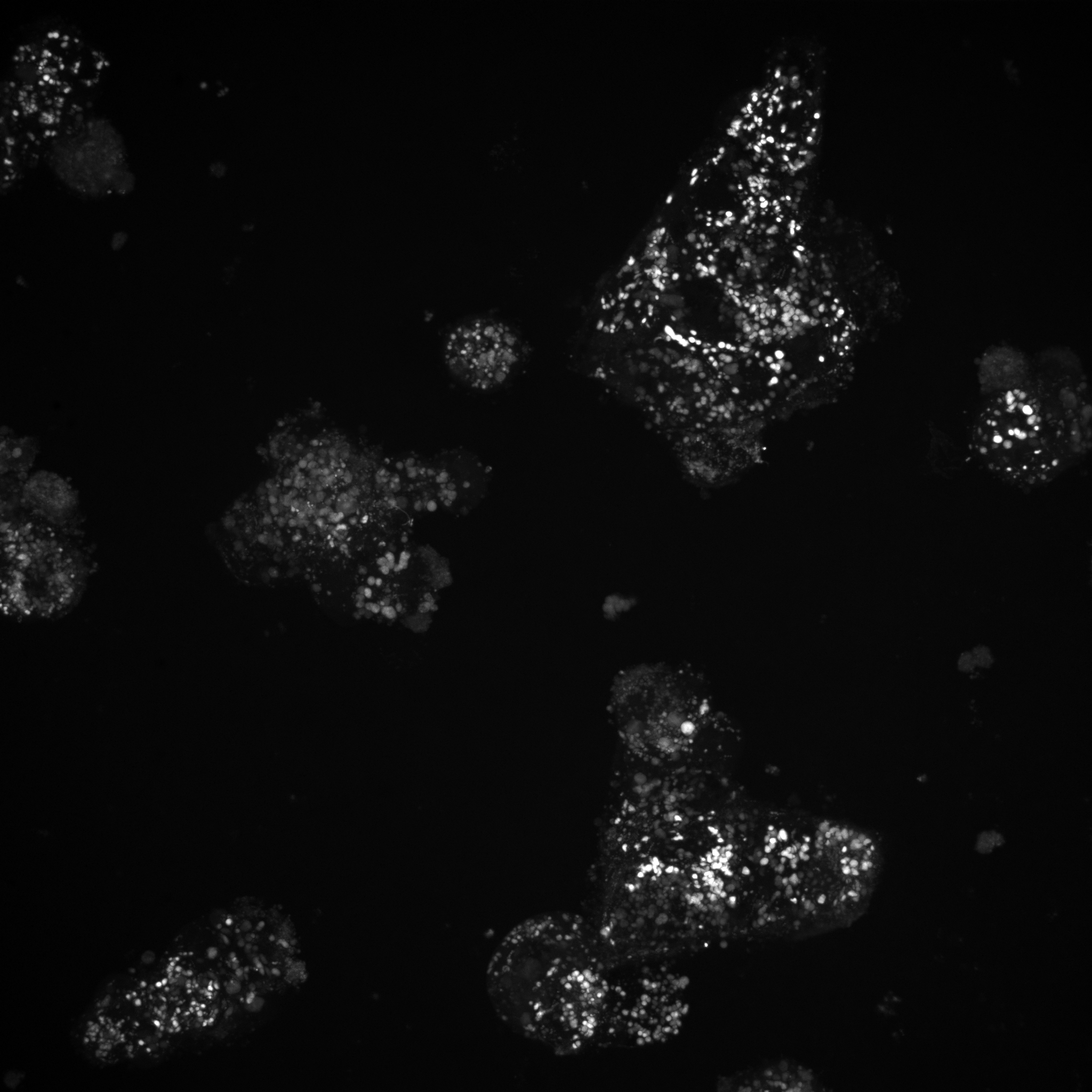

Supplement: Supplementary file 12 — Figure EV5 Source Data [file 44319_2025_581_MOESM12_ESM.zip › EV5/G/Untreatment top.tif]

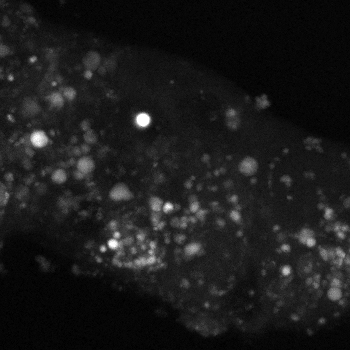

Supplement: Supplementary file 12 — Figure EV5 Source Data [file 44319_2025_581_MOESM12_ESM.zip › EV5/G/Wortmannin bottom.tif]

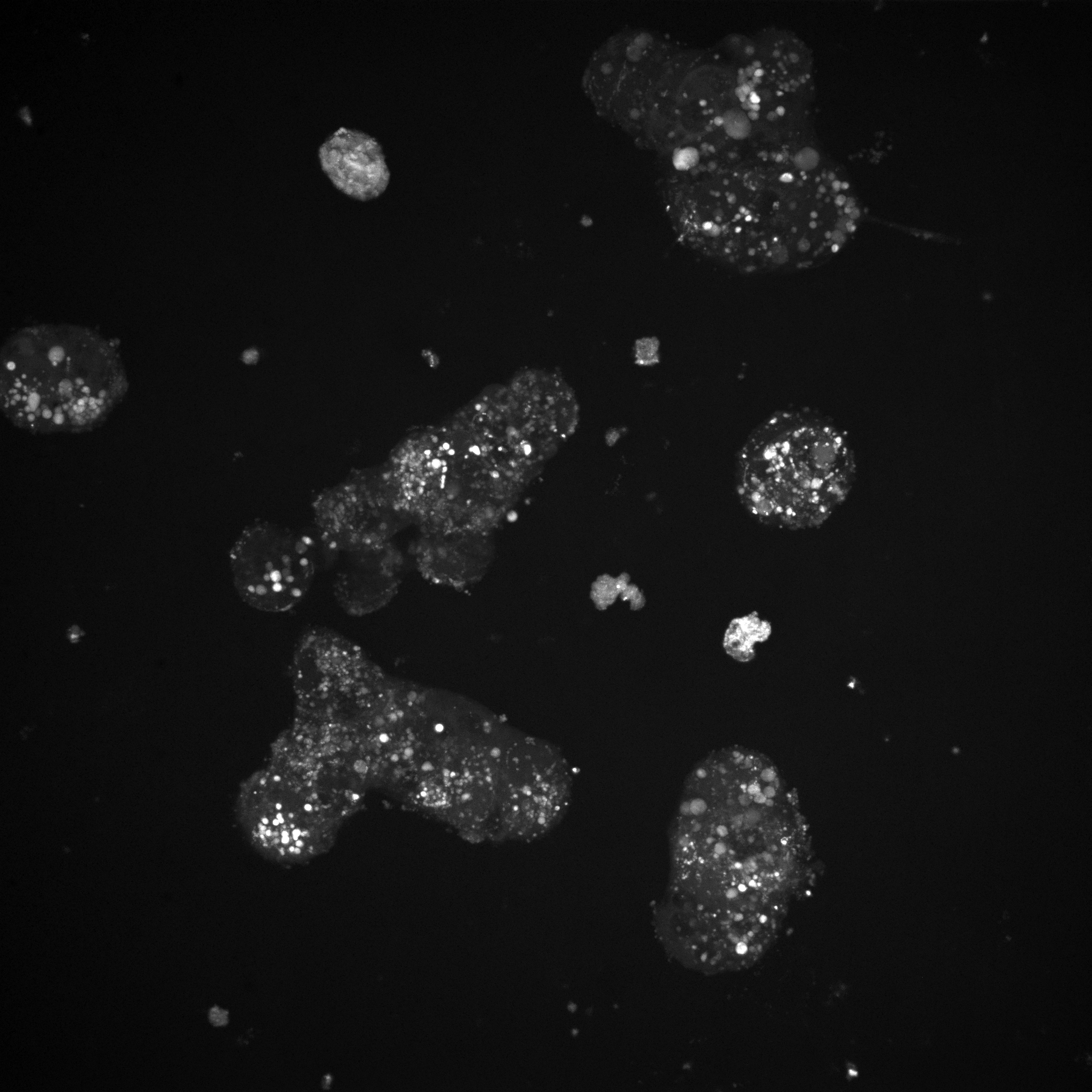

Supplement: Supplementary file 12 — Figure EV5 Source Data [file 44319_2025_581_MOESM12_ESM.zip › EV5/G/Wortmannin top.tif]
