# Supplementary material for: CDC42 supports HBV entry by NTCP translocation to the plasma membrane and macropinocytosis
Source: EMBO Rep. 2025 Sep 15;26(21):5239–69. doi: 10.1038/s44319-025-00581-8 (PMC12592336; doi:10.1038/s44319-025-00581-8)
Supplement: Supplementary file 13 — Expanded View Figures [file 44319_2025_581_MOESM13_ESM.pdf]

Expanded View Figures

Figure EV1. Effects of exogenous expression of CDC42-CA and CDC42-DN in HepG2-NTCP cells on cell growth and HBV infection.

(**A**) Equal numbers of HepG2-NTCP vector, CDC42-CA and CDC42-DN cells were plated in triplicate. Data are shown as fold changes to the vector group. $n = 3$. (**B**, **C**) Determination of the effect of bradykinin ((**B**), $n = 3$) and ML141 ((**C**), $n = 5$) on HepG2-NTCP cell viability by cytotoxicity assay. Data are shown as fold changes to the untreated group. (**D**) HepG2-NTCP cells were treated with bradykinin (100 ng/ml) or ML141 (50 μM) for 10 h. Cells were subjected to GTP-bound GTPase pulldown at indicated time points post treatment. CDC42 GTP-bound forms were analyzed by western blot. (**E**) Intracellular HBV DNA and secreted HBV virion DNA from HepG2-NTCP cells treated with bradykinin (100 ng/ml) or ML141 (50 μM) were purified at 7 dpi and measured by qPCR. Data are shown as fold changes to the untreated group. $n = 3$. Intracellular HBV DNA of Bradykinin: $P = 0.0002$; Intracellular HBV DNA of ML141: $P = 0.0005$; secreted virions of Bradykinin: $P = 0.0006$; secreted virions of ML141: $P = 0.0007$. (**F**) cccDNA was purified at 7 dpi and measured by qPCR. Data are shown as fold changes to the untreated group. $n = 3$. cccDNA of Bradykinin: $P = 0.0026$; cccDNA of ML141: $P = 0.0193$. (**G**) Viral HBeAg in the medium released from infected cells was quantified by ELISA. Data are shown as fold changes to the untreated group. $n = 3$. HBeAg of Bradykinin at 7 days post infection: $P = 0.0001$; HBeAg of Bradykinin at 7 days post infection: $P = 0.0007$; These experiments were repeated three times. Data are represented as mean ± SEM. A.U. Arbitrary Unit, ns no significant difference; *$P < 0.05$; **$P < 0.01$; ***$P < 0.001$ (unpaired t test).

© The Author(s)

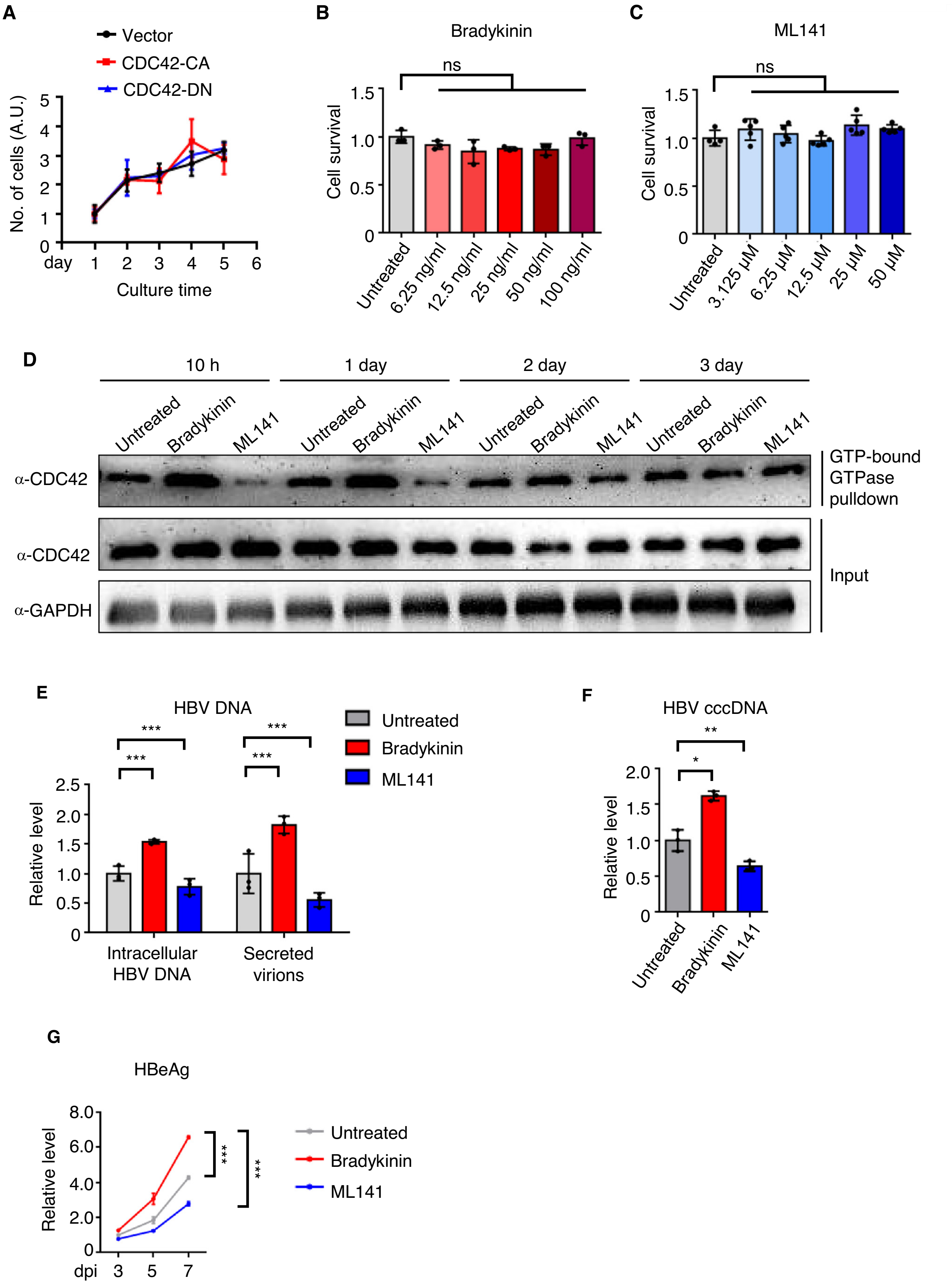
A
Vector
CDC42-CA
CDC42-DN
No. of cells (A.U.)
day
Culture time
B
Bradykinin
ns
Cell survival
Untreated
6.25 ng/ml
12.5 ng/ml
25 ng/ml
50 ng/ml
100 ng/ml
C
ML141
ns
Cell survival
Untreated
3.125 μM
6.25 μM
12.5 μM
25 μM
50 μM
D
10 h
1 day
2 day
3 day
Untreated
Bradykinin
ML141
α-CDC42
GTP-bound GTPase pulldown
α-CDC42
α-GAPDH
Input
E
HBV DNA
Untreated
Bradykinin
ML141
Relative level
Intracellular HBV DNA
Secreted virions
F
HBV cccDNA
Relative level
Untreated
Bradykinin
ML141
G
HBeAg
Relative level
dpi
Untreated
Bradykinin
ML141

 © The Author(s)

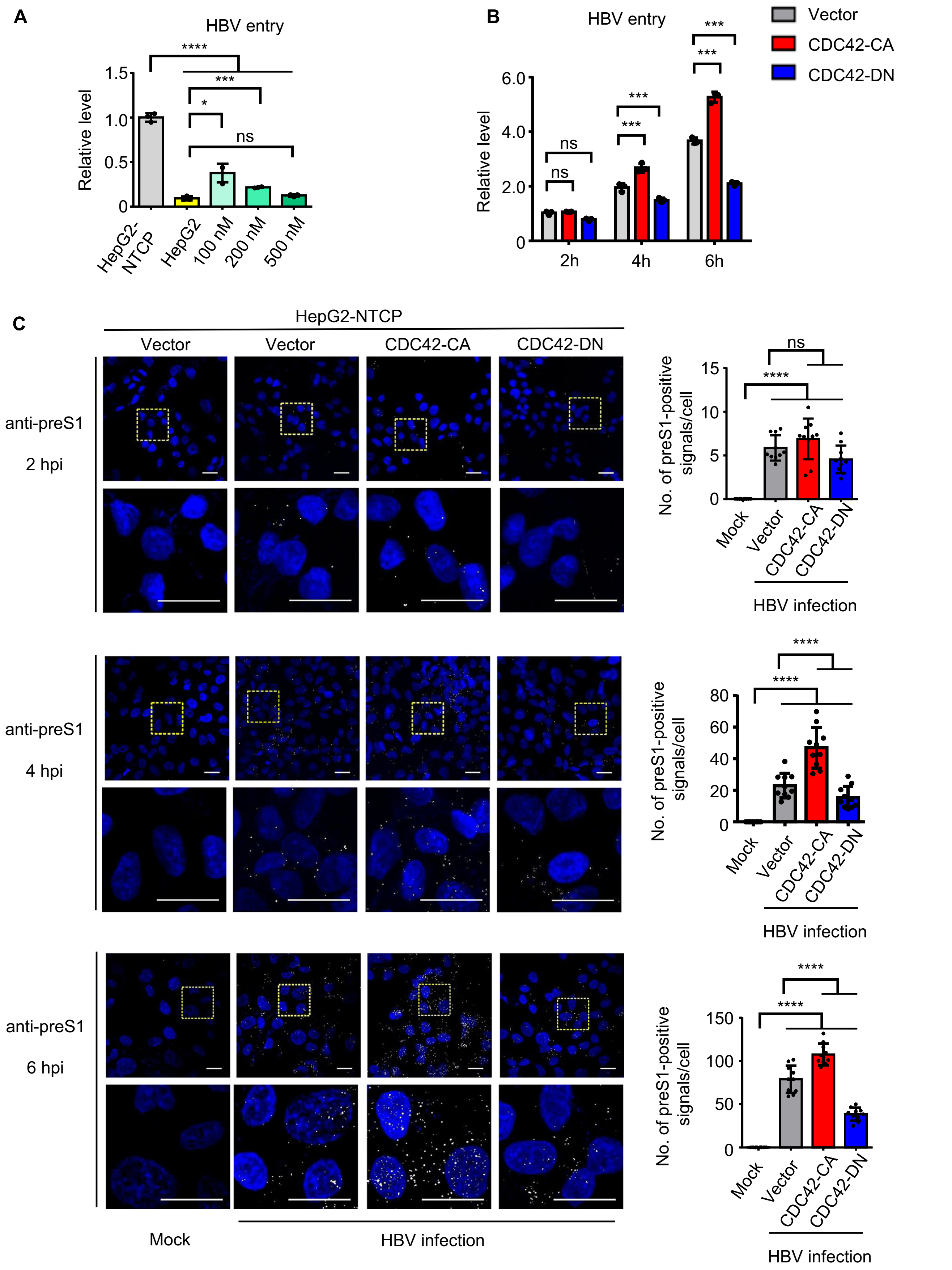
A
HBV entry
Relative level
HepG2-NTCP
HepG2
100 nM
200 nM
500 nM
B
HBV entry
Vector
CDC42-CA
CDC42-DN
Relative level
2h
4h
6h
C
HepG2-NTCP
Vector
Vector
CDC42-CA
CDC42-DN
anti-preS1
2 hpi
anti-preS1
4 hpi
anti-preS1
6 hpi
Mock
HBV infection
No. of preS1-positive signals/cell
Mock
Vector
CDC42-CA
CDC42-DN
HBV infection

Figure EV2. Effect of CDC42 on HBV entry and replication.

(**A**) HepG2-NTCP cells were infected with HBV in the presence of increasing concentrations of Myrcludex B (MyrB). HepG2 cells were used as negative control for infection. $n = 3$. Data are shown as fold changes to HepG2-NTCP group. HepG2, 100 nM, 200 nM and 500 nM MyrB versus HepG2-NTCP: $P < 0.0001$; 100 nM MyrB versus HepG2: $P = 0.0108$; 200 nM MyrB versus HepG2: $P = 0.0003$. (**B**) HepG2-NTCP vector, CDC42-CA and CDC42-DN cells were inoculated with heparin-purified HBV for 1 h at 4 °C and then transferred to 37 °C for 2 h, 4 h or 6 h. Intracellular HBV DNA was quantified by qPCR. $n = 3$. Data are shown as fold changes to vector group at 2 hpi. CDC42-CA at 4 h post infection: $P = 0.0009$; CDC42-DN at 4 h post infection: $P = 0.0007$; CDC42-CA at 6 h post infection: $P = 0.0002$; CDC42-DN at 6 h post infection: $P = 0.0001$. (**C**) Representative immunofluorescence images of HepG2-NTCP uninfected cells (Mock), HepG2-NTCP vector, CDC42-CA and CDC42-DN cells infected with HBV at 2, 4 and 6 hpi. HBV particles were stained with anti-preS1 antibody. Nucleus were stained with DAPI. Quantification of preS1-positive signals per cell is presented. $n = 10$ views (100×/1.5 oil objective). Vector, CDC42-CA and CDC42-DN versus mock at 2, 4 and 6 h post infection: $P < 0.0001$; CDC42-CA and CDC42-DN versus vector at 4 and 6 h post infection: $P < 0.0001$; Scale bar = 10 µm. Data are represented as mean ± SEM. ns, no significant difference; $^{*}P < 0.05$; $^{***}P < 0.001$; $^{****}P < 0.0001$ (unpaired t test). Source data are available online for this figure.

© The Author(s)

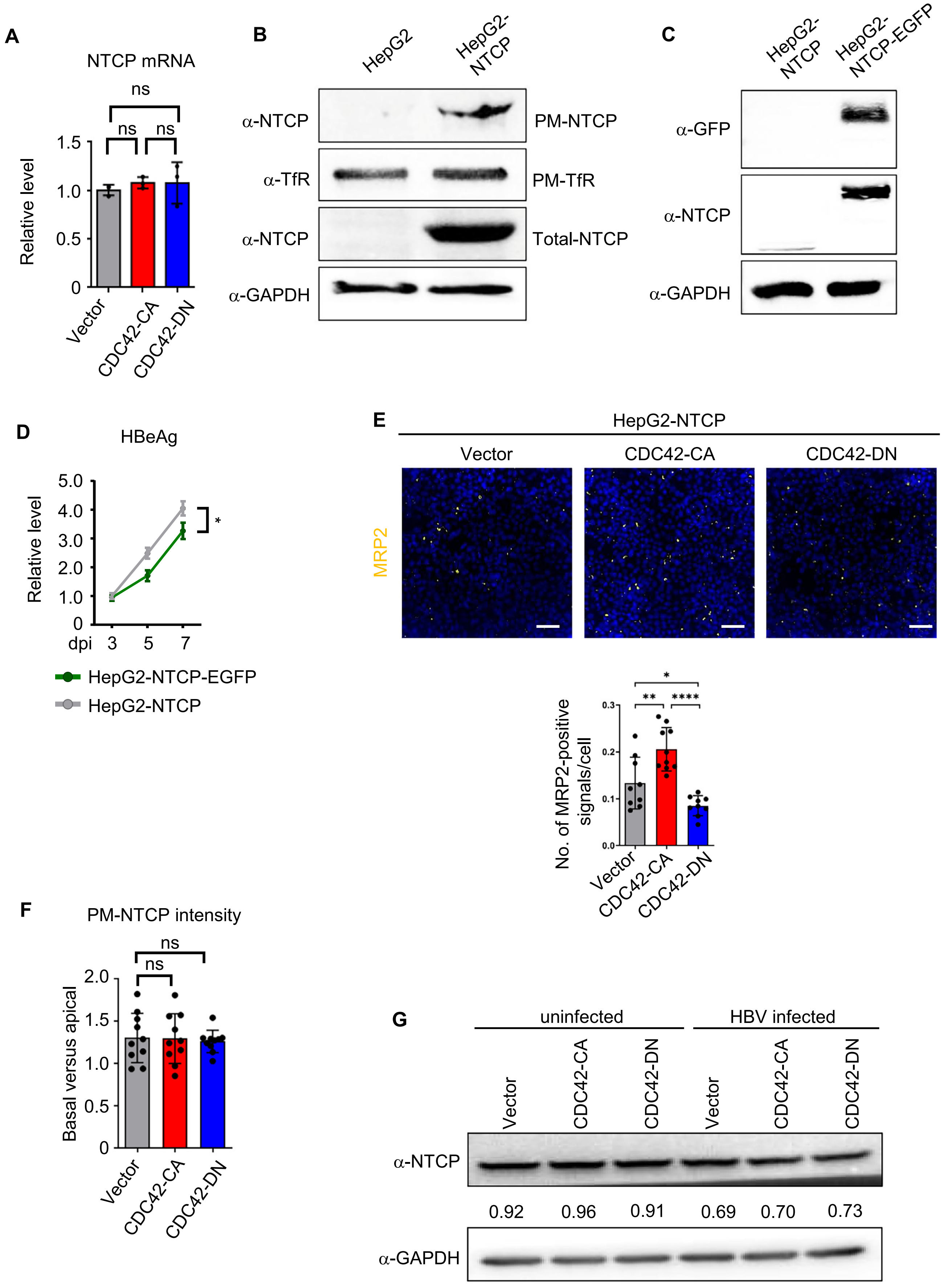
A
NTCP mRNA
ns
ns ns
Relative level
1.5
1.0
0.5
0
Vector
CDC42-CA
CDC42-DN
B
HepG2
HepG2-NTCP
α-NTCP
PM-NTCP
α-TfR
PM-TfR
α-NTCP
Total-NTCP
α-GAPDH
C
HepG2-NTCP
HepG2-NTCP-EGFP
α-GFP
α-NTCP
α-GAPDH
D
HBeAg
Relative level
5.0
4.0
3.0
2.0
1.0
0
dpi 3 5 7
*
HepG2-NTCP-EGFP
HepG2-NTCP
E
HepG2-NTCP
Vector
CDC42-CA
CDC42-DN
MRP2
No. of MRP2-positive signals/cell
0.3
0.2
0.1
0.0
*
**
****
Vector
CDC42-CA
CDC42-DN
F
PM-NTCP intensity
ns
ns
Basal versus apical
2.0
1.5
1.0
0.5
0
Vector
CDC42-CA
CDC42-DN
G
uninfected
HBV infected
Vector
CDC42-CA
CDC42-DN
Vector
CDC42-CA
CDC42-DN
α-NTCP
0.92
0.96
0.91
0.69
0.70
0.73
α-GAPDH

Figure EV3. Detection of NTCP expression and the effects of NTCP-EGFP fusion protein on HBV replication.

(**A**) Quantification of NTCP mRNA by qPCR in HepG2-NTCP vector, CDC42-CA and CDC42-DN cells. Data are presented as fold changes to the vector group. $n = 3$. (**B**) Plasma membrane (PM) proteins were purified from HepG2 and HepG2-NTCP cells by biotin labeling. PM and total proteins were subjected to Western blotting analysis. The plasma membrane transferrin receptor (PM-TfR) and GAPDH were used as loading control of plasma membrane proteins and total proteins, respectively. (**C**) Indicated proteins in HepG2-NTCP and HepG2-NTCP-EGFP cells were detected by Western blotting. (**D**) HepG2-NTCP and HepG2-NTCP-EGFP cells were infected with HBV for 8 h. Culture supernatant was collected at indicated time points for HBeAg analysis by ELISA. These experiments were repeated three times. $n = 3$. HepG2-NTCP-EGFP at 7 days post infection: $P = 0.0231$. (**E**) Immunostaining of MRP2 in HepG2-NTCP vector, CDC42-CA and CDC42-DN cells. Representative images are presented. MRP2-positive signals were quantified. $n = 10$ views (40×/1.5 air objective). Scale bar = 10 µm. CDC42-CA versus vector: $P = 0.0035$; CDC42-DN versus vector: $P = 0.0264$; CDC42-DN versus CDC42-CA: $P < 0.0001$. (**F**) NTCP-EGFP fusion protein was quantified at the basal and apical surfaces of HepG2-NTCP-EGFP vector, CDC42-CA and CDC42-DN cells. The values are presented as the ratio of EGFP intensity at the basal surface to that at the apical surface. $n = 10$. (**G**) Total NTCP expression was detected by Western blot in HepG2-NTCP vector, CDC42-CA and CDC42-DN cell lines infected or uninfected with HBV. Cells were collected at 7 dpi. GAPDH was used as loading control. The ratio of NTCP versus GAPDH is presented. Data are represented as mean ± SEM. ns no significant difference; *$P < 0.05$; **$P < 0.01$; ****$P < 0.0001$ (unpaired t test). Source data are available online for this figure.

 © The Author(s)

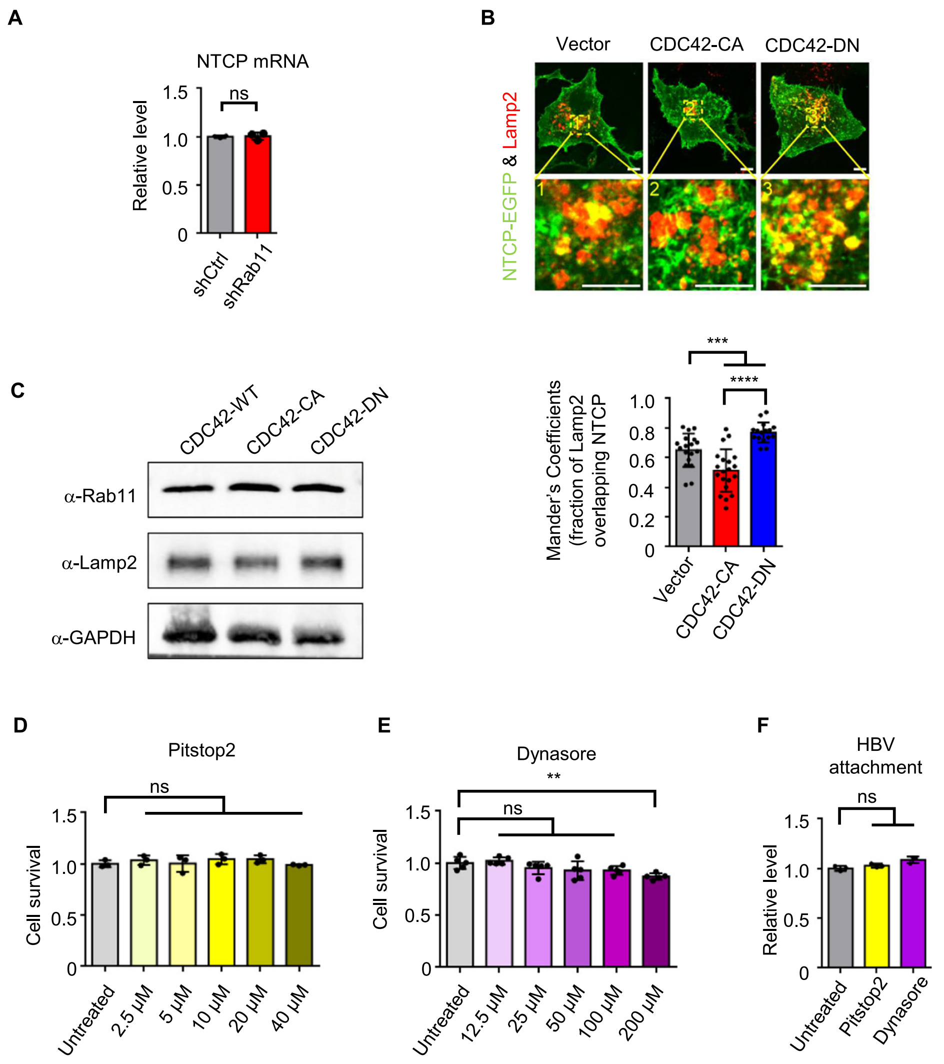

Figure EV4. Effects of knockdown of Rab11 on NTCP mRNA expression and cytotoxicity assays of Pitstop2 and Dynasore.

(**A**) Quantification of NTCP mRNA by qPCR in HepG2-NTCP shControl (shCtrl) and shRab11 cells. Data are shown as fold changes to the shCtrl group. $n = 3$.
(**B**) Representative immunofluorescence images of NTCP-EGFP co-localization with late endosomes/lysosomes in HepG2-NTCP vector, CDC42-CA and CDC42-DN cells. The yellow box indicates the magnified area shown in the corresponding bottom panel. Late endosomes/lysosmes were stained with anti-Lamp2 antibody. Quantification of Mander's coefficients of NTCP and late endosomes/lysosomes. $n = 20$ views (100×/1.5 oil objective). CDC42-CA versus vector: $P = 0.0009$; CDC42-DN versus vector: $P = 0.0008$; CDC42-DN versus CDC42-CA: $P < 0.0001$; Scale bar = 10 μm. (**C**) Western blotting analysis of indicated proteins from cellular extract of HepG2-NTCP CDC42-WT, CDC42-CA and CDC42-DN cells. GAPDH is used as loading control. (**D**, **E**) Cytotoxicity assays of pitstop2 ((**D**), $n = 3$) and dynasore ((**E**), $n = 5$) at different concentrations on HepG2-NTCP cell viability. 200 μM dynasore: $P = 0.0024$; Data are shown as fold changes to the untreated group. (**F**) Quantification of HBV DNA in HepG2-NTCP cells treated with indicated reagents at 3 hpi by qPCR. Data are shown as fold changes to the untreated group. $n = 3$. These experiments were repeated three times. Data are represented as mean ± SEM in all quantification panels. ns, no significant difference; $^{**}P < 0.01$; $^{***}P < 0.001$; $^{****}P < 0.0001$ (unpaired *t* test). Source data are available online for this figure.

© The Author(s)

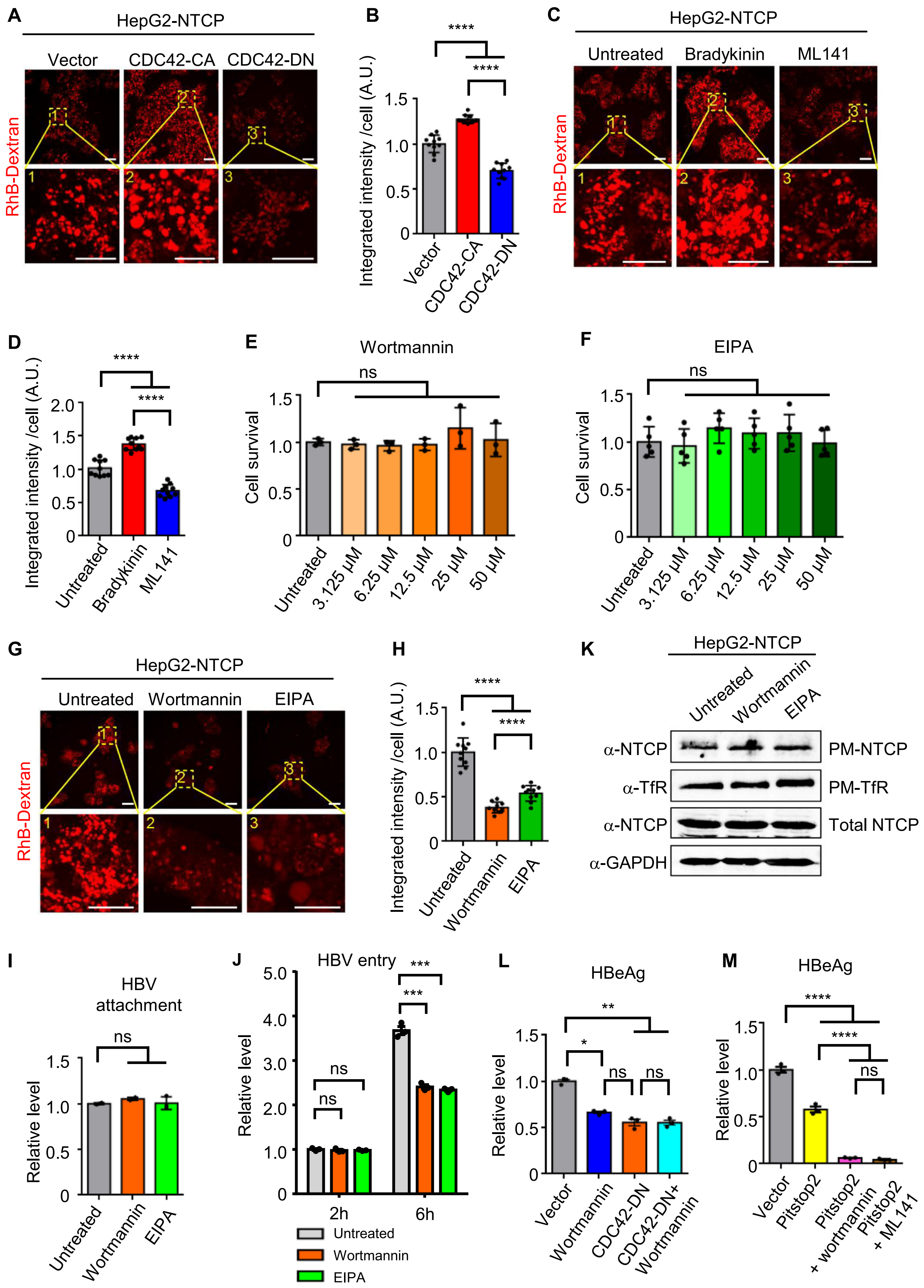
A
HepG2-NTCP
Vector
CDC42-CA
CDC42-DN
RhB-Dextran
B
Integrated intensity /cell (A.U.)
Vector
CDC42-CA
CDC42-DN
C
HepG2-NTCP
Untreated
Bradykinin
ML141
RhB-Dextran
D
Integrated intensity /cell (A.U.)
Untreated
Bradykinin
ML141
E
Wortmannin
ns
Cell survival
Untreated
3.125 μM
6.25 μM
12.5 μM
25 μM
50 μM
F
EIPA
ns
Cell survival
G
HepG2-NTCP
Untreated
Wortmannin
EIPA
RhB-Dextran
H
Integrated intensity /cell (A.U.)
K
HepG2-NTCP
Untreated
Wortmannin
EIPA
α-NTCP
PM-NTCP
α-TfR
PM-TfR
Total NTCP
α-GAPDH
I
HBV attachment
Relative level
J
HBV entry
Relative level
2h
6h
Untreated
Wortmannin
EIPA
L
HBeAg
Relative level
Vector
Wortmannin
CDC42-DN
CDC42-DN+ Wortmannin
M
HBeAg
Relative level
Vector
Pitstop2
Pitstop2 + wortmannin
Pitstop2 + ML141

Figure EV5. CDC42 regulates macropinocytosis in HBV infection.

(**A**) Dextran uptake assay in HepG2-NTCP vector, CDC42-CA and CDC42-DN cells. Cells were incubated with Rhodamine B Dextran (RhB-Dextran) for 1 h and then washed. Dextran staining is shown in red. Scale bar = 10 μm. (**B**) Quantification of fluorescence intensity of Rhodamine B-Dextran within each cell in A. Data are shown as fold changes to the vector group. $n = 15$ views (100×/1.5 oil objective). CDC42-CA and CDC42-DN versus vector, CDC42-DN versus CDC42-CA: $P < 0.0001$. (**C**) Dextran uptake assay in HepG2-NTCP cells treated with bradykinin or ML141. Dextran staining is shown in red. Scale bar = 10 μm. (**D**) Quantification of fluorescence intensity of Rhodamine B-Dextran within each cell in C. Data are shown as fold changes to the untreated group. n = 15 views (100×/1.5 oil objective). Bradykinin and ML141 versus untreated, ML141 versus Bradykinin: $p < 0.0001$. (**E**, **F**) Cytotoxic assays of wortmannin ((**E**), $n = 3$) and EIPA ((**F**), $n = 5$) at different concentrations on HepG2-NTCP cell viability. Data are shown as fold changes to the untreated group. (**G**) Dextran uptake assay in HepG2-NTCP cells treated with wortmannin (50 μM) or EIPA (50 μM). Dextran staining is shown in red. Scale bar = 10 μm. (**H**) Quantification of fluorescence intensity of Rhodamine B-Dextran within each cell in (**G**). Data are shown as fold changes to untreated group. $n = 15$ views (100×/1.5 oil objective). Wortmannin and EIPA versus untreated, EIPA versus Wortmannin: $P < 0.0001$. (**I**) Quantification of HBV DNA in HepG2-NTCP cells treated with indicated reagents at 3 hpi by qPCR. Data are shown as fold changes to the untreated group. $n = 3$. (**J**) Quantification of internalized HBV DNA in HepG2-NTCP cells untreated or treated with wortmannin (50 μM) or EIPA (50 μM) at 2 hpi and 6 hpi by qPCR. Data are shown as fold changes to the untreated group at 2 hpi. $n = 3$. Wortmannin at 6 hpi: $P = 0.0003$; EIPA at 6 hpi: $P = 0.0002$. (**K**) Plasma membrane proteins were purified from HepG2-NTCP cells treated with wortmannin (50 μM) and EIPA (50 μM) by biotin labeling. PM and total proteins were subjected to Western blotting analysis. The plasma membrane transferrin receptor (PM-TfR) and GAPDH were used as loading control of plasma membrane proteins and total proteins, respectively. (**L**, **M**) Culture supernatant from HBV-infected HepG2-NTCP vector or HepG2-NTCP CDC42-DN cells treated with indicated reagents was collected at 7 dpi. HBeAg was quantified by ELISA. Data are shown as fold changes to the vector group. These experiments were repeated three times. $n = 3$. Wortmannin versus vector: $P = 0.0175$; CDC42-DN versus vector: $P = 0.0012$; CDC42-DN + wortmannin versus vector: $P = 0.0010$; Pitstop2, Pitstop2 + Wortmannin and Pitstop2 + ML141 versus vector, Pitstop2 + Wortmannin and Pitstop2 + ML141 versus Pitstop2: $P < 0.0001$; Data are represented as mean ± SEM. A.U. Arbitrary Unit, ns no significant difference; $^{*}P < 0.05$; $^{**}P < 0.01$; $^{***}P < 0.001$, $^{****}P < 0.0001$ (unpaired t test). Source data are available online for this figure.

© The Author(s)
